# Replication fork rescue in mammalian mitochondria

**DOI:** 10.1038/s41598-019-45244-6

**Published:** 2019-06-19

**Authors:** Rubén Torregrosa-Muñumer, Anu Hangas, Steffi Goffart, Daniel Blei, Gábor Zsurka, Jack Griffith, Wolfram S. Kunz, Jaakko L. O. Pohjoismäki

**Affiliations:** 1 Department of Environmental and Biological Sciences, University of Eastern Finland, P.O. Box 111, 80101 Joensuu, Finland; 2 Research Programs Unit, Molecular Neurology, University of Helsinki, Helsinki, Finland; 3 Department of Experimental Epileptology and Cognition Research, University of Bonn, Sigmund-Freud-Str. 25, Bonn, D-53105 Germany; 4 Lineberger Comprehensive Cancer Center, University of North Carolina at, Chapel Hill, USA

**Keywords:** Stalled forks, DNA damage and repair, DNA recombination

## Abstract

Replication stalling has been associated with the formation of pathological mitochondrial DNA (mtDNA) rearrangements. Yet, almost nothing is known about the fate of stalled replication intermediates in mitochondria. We show here that replication stalling in mitochondria leads to replication fork regression and mtDNA double-strand breaks. The resulting mtDNA fragments are normally degraded by a mechanism involving the mitochondrial exonuclease MGME1, and the loss of this enzyme results in accumulation of linear and recombining mtDNA species. Additionally, replication stress promotes the initiation of alternative replication origins as an apparent means of rescue by fork convergence. Besides demonstrating an interplay between two major mechanisms rescuing stalled replication forks – mtDNA degradation and homology-dependent repair – our data provide evidence that mitochondria employ similar mechanisms to cope with replication stress as known from other genetic systems.

## Introduction

DNA replication is an inherently complex cellular event, where any fault in the processes can have dire consequences for the cell. In contrast to test tube conditions, DNA synthesis in a cell is not just a polymerization reaction with standard kinetic properties. Instead, replication forks stall frequently as the replisome encounters template damage, transcription complexes, positive superhelical torsion and protein-DNA complexes^1,2^. If replication fork arrest is not relieved in a controlled manner, the condition will eventually result in a double-strand break and the loss of the non-replicated part of the genome in the absence of repair. Consequently, both prokaryotic and eukaryotic cells have evolved a vast arsenal of tools and strategies to remove the various obstacles blocking replisome progression as well as to deal with the DNA damage caused by replication failure.

Compared to nuclear genomes, the mitochondrial DNA (mtDNA) is tiny, 16–19 kb in most metazoans, and exists in hundreds to thousands of copies per cell^3^. For a long time it has been thought that mitochondria lack most of the DNA repair pathways existing in the nucleus, therefore preferring mtDNA maintenance quantity over quality, and damaged molecules would be targeted for degradation rather than investing in their repair^4^. While the latter could be true for post-mitotic or ephemeral cells in some organisms^5^, exhaustive mutation pattern analyses from mouse have concluded that replication errors – not damage – are the main sources of mtDNA mutations^6,7^. Similar to nuclear replication forks, mtDNA replication arrests at sites of template damage^8,9^, on naturally occurring replication pause sites controlling transcription complex bypass^10,11^ or due impaired enzyme function caused by pathological mutations in the replicative helicase TWNK or mitochondrial DNA polymerase Pol γ^12^^–^^14^. Double-strand breaks resulting from replication stress have been suggested to be the main culprits behind the pathological mtDNA rearrangements observed in many human conditions^15,16^.

In contrast to nuclear genome replication, mtDNA replication in mammals is mostly strand-asynchronous, with the lagging strand synthetized with a considerable delay compared to the leading strand^17,18^. Replication of the leading strand (heavy or H-strand, due to its higher G to C ratio) initiates at one major origin (O_H_), resulting in a displaced single-stranded DNA (ssDNA) intermediate, protected by mtSSB^18^ and/or active hybridization with preformed RNA^19^. Compared to the H-strand, the initiation of the lagging strand (light or L-strand) is considerably more flexible. The major L-strand origin (O_L_)^16,20^ is complemented by more-or-less frequent lagging strand initiation at undefined sites^21,22^. The uncoupled leading and lagging strand synthesis means that potential template damage has very different consequences for the synthesis of the two strands. Both in prokaryotes and in the eukaryotic nucleus, damage in the lagging strand template does not significantly hamper replication progression^1,2^, as expected from the discontinuous nature of its DNA synthesis – new Okazaki-fragments are constantly primed independently from the preceding ones. If the replisome stalls at a damaged site during leading strand synthesis, a dedicated translesion polymerase can be recruited to the replication fork to synthesize across the damage^1^. Alternatively, replication of the two strands can be uncoupled with the replicative helicase continuing to unwind while the lagging strand is synthetized. This will result in lesion skipping by upstream priming or fork regression and reset by origin-independent restart^1,2^. Both, regressed forks as well as broken, partially replicated molecules resulting from the cleavage of stalled replication fork, can be repaired by recombination-dependent replication^23^.

We have previously shown that the archaic primase-polymerase PrimPol is required for lesion skipping and promiscuous lagging strand priming in mammalian mitochondria^22^. The involvement of PrimPol in lagging strand priming is highly interesting as it helps to understand some aspects of basic mtDNA maintenance mechanisms, such as the occurrence of fully double-strand DNA (dsDNA) mtDNA replication intermediates that have been previously associated with strand-coupled replication^24–26^. While it is impossible to differentiate between frequent lagging strand priming and true strand-coupled replication, this might not be mechanistically important. Recent insight into the genome replication of bacteria has demonstrated that DNA replication is often dynamic, with frequent uncoupling and recoupling of leading and lagging strand synthesis^27^. In essence, replication coupling is mechanistically arbitrary, as fully dsDNA mtDNA replication intermediates can arise when L-strand synthesis catches up with the H-strand synthesis.

Regardless of their origin, fully dsDNA (i. e. RNaseH and ssDNA cleaving enzyme resistant) replication intermediates of mtDNA exist in all tissues, being predominant in metabolically active post-mitotic tissues^28,29^. Interestingly, mtDNA damage in rapidly dividing cultured cells results in dramatic accumulation of partially ssDNA replication intermediates, but also increases the abundance of fully dsDNA intermediates^8^. In the presented study, we reveal that these fully dsDNA replication forks can regress upon replication stalling. Although fork regression-dependent replication rescue has been well characterized in other systems^30–32^, our results are the first demonstration of this mechanism occurring in mitochondria, revealing biologically relevant differences between dsDNA and ssDNA replication intermediates. Furthermore, we are able to demonstrate that mitochondria have additional genome safeguarding mechanisms, such as the ability to initiate replication twice on the same template to converge replication forks. Important for human pathological conditions, our data also show that the stalled forks frequently break, resulting in linear fragments that contribute to the formation of mtDNA recombination intermediates unless degraded by an MGME1-dependent pathway.

## Results

**Figure 1:**
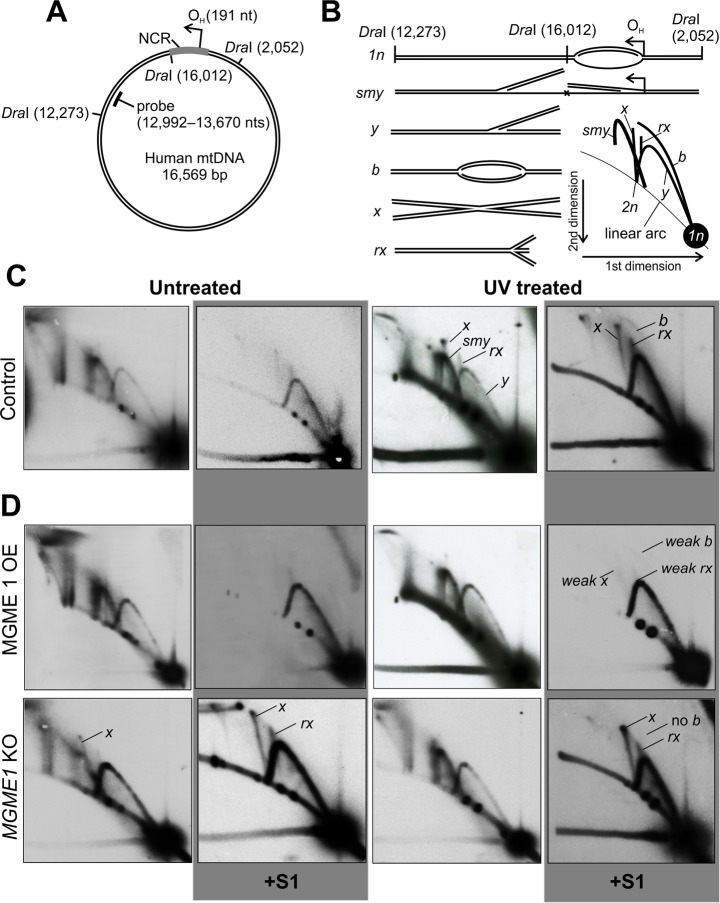
Effects of UV induced replication stalling in HEK293T cells. (**A**) A schematic map of human mitochondrial DNA (mtDNA) showing the *Dra*I restriction sites adjacent to the non-coding region (NCR); other *Dra*I sites are omitted for clarity. Different forms of the *Dra*I fragment downstream of the origin of H-strand replication (O_H_) were analyzed using two-dimensional agarose gel electrophoresis (2D-AGE). Location of the probe used for Southern hybridization as indicated. (**B**) Illustration of the different mtDNA forms detected by the probe. Linear forms spanning one restriction fragment length (*1n*; nts 12,273–16,012) are the most abundant. As O_H_ is not included in the analyzed region, asynchronous (or so-called strand-displacement) replication forks initiating from this origin enter the fragment as they proceed, resulting in slow-moving *y*-forms (*smy*’s) due to the inability of the restriction enzyme to cut the displaced strand. If replication intermediates are fully double-stranded DNA, both leading and lagging strand are cut, resulting in regular *y*-forms. Replication bubbles originating from replication initiation within the *Dra*I fragment will migrate on the bubble arc (*b*). Recombination junctions consist of two restriction fragment-sized linear molecules and form an *x*-spike that starts from the *2n* position of the linear arc. Regressed replication forks (*rx*) form specific chicken foot structures growing from the *y*-arc^30^. (**C**) 2D-AGE of the *Dra*I 12,273–16,012 fragment of mtDNA from HEK293T cells before (untreated) and 4 h after 30 s exposure to 1.34 mJ/cm^2^, 302 nm wavelength UVB (UV treated). S1 nuclease treatment (panels with gray background) was applied to reveal fully double-stranded DNA (dsDNA) forms. Note the appearance of the different *x*-forms, replication bubble (*b*) and the overall increase in dsDNA intermediates. (**D**) MGME1 overexpression (OE) reduces all *x*-forms, whereas knockout (KO) of *MGME1* has an opposite effect along with disappearance of the bubble arc.

We have previously reported that UV and oxidative damage as well as inhibition of mitochondrial DNA polymerase Pol γ by ddC result in an increase in dsDNA intermediates concomitantly with the appearance of cruciform (*x*-form) mtDNA^8^. To distinguish these *x*-forms from resembling replication termination intermediates with two meeting replication forks that occur at the non-coding region (NCR) of mtDNA^33^, we focused on a region spanning nts 12,273–16,012 of mtDNA, generated by *Dra*I restriction (Fig. 1A). The NCR, including the main leading strand origin (O_H_) and the site of termination, lies outside of this fragment and any strand-asynchronous replication entering the fragment will give rise to so-called slow moving *y*-arc intermediates (*smy*) due to the inability of *DraI* to cleave the displaced ssDNA strand (Fig. 1A,B). Fully dsDNA replication intermediates in contrast are cleaved by *Dra*I to generate *y*-forms (*y*). Replication initiating within the analyzed fragment will give rise to replication bubbles (*b*). All cruciform DNA will be double-stranded and consist of recombination junctions with equally long arms (*x*) or trident-shaped DNA (*rx*), the latter generated by regression of the replication fork. Both *x*-forms can be enhanced by UV treatment (Fig. 1C, Supplementary Fig. S2) and are resistant to the single-stranded DNA-digesting S1 nuclease, which also removes the RNA:DNA hybrid containing intermediates^34^. Regressed forks and recombination junctions identical to these *x*-forms are known to accumulate during replication stalling also in other genomes^30–32^. Note also the relative increase in the dsDNA *y*-forms and the appearance of replication bubbles (*b*) after UV exposure (Fig. 1C, Supplementary Fig. S2).

Homology-dependent rescue of broken and reversed forks in prokaryotes as well as the eukaryotic nucleus requires the action of a recombinase^1,2^. Although no dedicated recombinases have been reported from mammalian mitochondria, the replicative helicase TWNK has substantial strand-exchange activity *in vitro* and is involved in *x*-form mtDNA induction in a transgenic animal model^29,35^ as well as in the human heart^36^. Overexpression of TWNK in cells under normal culture conditions results in an increase of dsDNA replication intermediates (*y*) and reduced levels of all *x*-forms (Supplementary Fig. S3A). Remarkably, TWNK overexpression almost completely prevents fork regression following UV exposure (no *rx*). As knockout of TWNK results in loss of mtDNA, we decided to use cells expressing the dominant-negative TWNK-LD mutation to see if fork regression and recombination junction formation are dependent on the helicase activity. TWNK-LD represents a clinically relevant patient mutation with a 13 aa duplication in the so-called linker region (352–364), resulting in severe impairment of the helicase activity, mtDNA replication stalling and depletion of copy number^12^. Interestingly, expression of the mutant TWNK did not prevent the formation of either *x*-form, but dramatically increased the amount of bubble structures (*b*) (Supplementary Fig. S3B).

The mitochondrial DNA exonuclease MGME1 has been shown to be involved in the turnover of linear mtDNA^37^. Therefore, we aimed to address its role in the processing of stalled dsDNA replication intermediates, which can generate double-strand breaks when broken. Overexpression of MGME1 in HEK293cells increased the abundance of dsDNA replication intermediates (*y*) at the expense of strand-displacement intermediates (*smy*) and reduced levels of all x-forms, similar to TWNK overexpression. MGME1 overexpression also strongly reduced the UV-induced formation of cruciform (*x* and *rx*) mtDNA species (Fig. 1D). The knockout (KO) of *MGME1* had high basal levels of regressed forks and recombination junctions compared to controls. Upon UV treatment the knockout cells showed a clear increase of mtDNA *x*-forms, but still showed a strong shift towards dsDNA (*y*) mitochondrial replication intermediates (Fig. 1D). While MGME1 overexpression and knockout as well as TWNK overexpression prevented replication initiation within the studied fragment, none of the manipulations influenced the UV-dependent increase in *smy*-forms upon UV exposure as reported for PrimPol^22^.

As *MGME1* KO cells showed the most prominent recombination intermediates (*x*-forms) we have ever observed in cultured cells, we hypothesized these to be linked with the high levels of linear mtDNA forms that appear as a consequence of MGME1 loss^37–40^. These 11 kb subgenomic linear fragments map rather precisely to the region between between O_H_ and O_L_^40^ and are similar to the ones observed in mice expressing exonuclease-deficient Pol γ (the so-called mutator mice)^41^. The O_H_–O_L_ fragments have been suggested to originate from replication fork breakage at a nick left of the replication origin due to a ligation defect caused by incomplete processing of flap structures at these loci^42^. The persistence of these subgenomic linear forms both in the mutator mouse as well as in the *MGME1* knockout is explained by the fact that both the 5´-exonuclease activity of MGME1 and the 3´-exonuclease activity of Pol γ are required for the turnover of broken mtDNA^37^. We reasoned that increased replication stress results in more frequent double-strand breaks, which are enriched in *MGME1* knockout cells due their inability to turn over broken molecules. The accumulation of linear mtDNA forces the cells to deploy other means to their disposal, such as recombination-dependent repair, which is detectable by an increase of *x*-forms.

**Figure 2:**
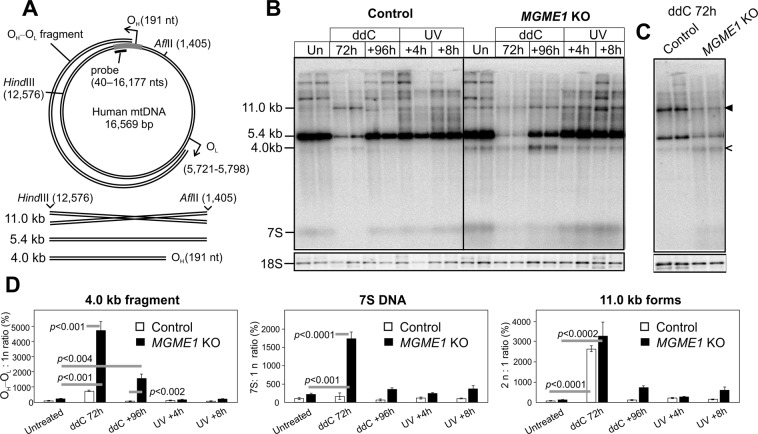
Replication stalling induces mtDNA double-strand breaks. (**A**) A schematic illustration of human mtDNA showing the linear deletion spanning between O_H_ and O_L_, restriction sites, the probe location and the resulting restriction fragments detected by the probe. In *Afl*II-*Hind*III digested samples, the probe will detect a 5.4 kb full-length mtDNA fragment, a 4.0 kb fragment originating from the O_H_–O_L_ species and 7 S DNA. As *x*-forms are twice the size of the full-length *Afl*II-*Hind*III fragment, they will migrate at 11.0 kb on an agarose gel in the absence of ethidium bromide. (**B**) Southern blot of *Afl*II-*Hind*III digested total DNA from ddC or UV treated control and *MGME1* knockout cells, probed for mtDNA as indicated in (**A**). In the case of ddC experiments, the cells were first exposed to ddC for 72 h to achieve maximal replication inhibition and then left to recover for 96 h. UV-treated cells were harvested 4 h or 8 h after exposure. *MGME1* knockout (KO) cells have high levels of the 4.0 kb subgenomic fragment, representing the O_H_–O_L_ species, and the 7 S DNA, but also a 11.0 kb molecular species corresponding to the abundant *x*-forms present in the samples (Fig. 1D). This 11.0 kb band appears also in the ddC -treated parental HEK293T cells (black arrowhead). While UV exposure increases the heterogeneous high molecular weight forms, it does not influence the levels of 4.0 kb fragments, 11.0 kb molecules or 7 S DNA. Additional bands larger than 11.0 kb represent partially cut mtDNA due to the ribonucleotide blockage of restriction sites^63^. The 18 S signal of nuclear DNA is given as a loading control. (**C**) Digest of parental HEK293T and *MGME1* knockout samples after 72 h ddC treatment as in (**B**), but with four-fold more DNA compared to (**B**). 4.0 kb fragments (open arrowhead) are present at low levels also in the parental control cells. The black arrowhead indicates 11.0 kb forms. (**D**) Quantifications of the various forms normalized against the full-length mtDNA in each sample and standardized against the corresponding levels in the untreated control (100%). Results are presented as mean ± SD and *p*-values are obtained from one-way ANOVA with post-hoc Tukey HSD Test. Note that the 47-fold increase in the 4.0 kb fragment at 72 h ddC samples of MGME1 KO masks the difference between the knockouts and controls in the untreated samples. In principle, the 4.0 kb fragment is below detection level in untreated control cells and its increase in the quantification is relative to the background in the corresponding lanes. Therefore no *p*-value is given for the comparison of untreated and 72 h ddC treated control samples.

As expected, the *MGME1* KO HEK293 cells had high levels of the linear O_H_–O_L_ fragment and 7 S DNA (Fig. 2), similar to the MGME1 patients^39^ and knockout mice^38^. While the level of the subgenomic fragment did not rise upon mild stalling caused by UV treatment, enforced replication after ddC treatment increased it 40-fold compared to untreated cells (Fig. 2B: +96 h, 4 kb fragment in *Afl*II + *Hind*III digest). Interestingly, a similar fragment as observed in the *MGME1* KO cells also appeared in parental HEK293T cells upon ddC treatment, together with an additional band corresponding to an mtDNA dimer in size (Fig. 2C). Whereas the O_H_–O_L_ fragment was lost in normal HEK293T cells during the recovery after the ddC treatment, high levels of the fragment remained in *MGME1* KO cells.

**Figure 3:**
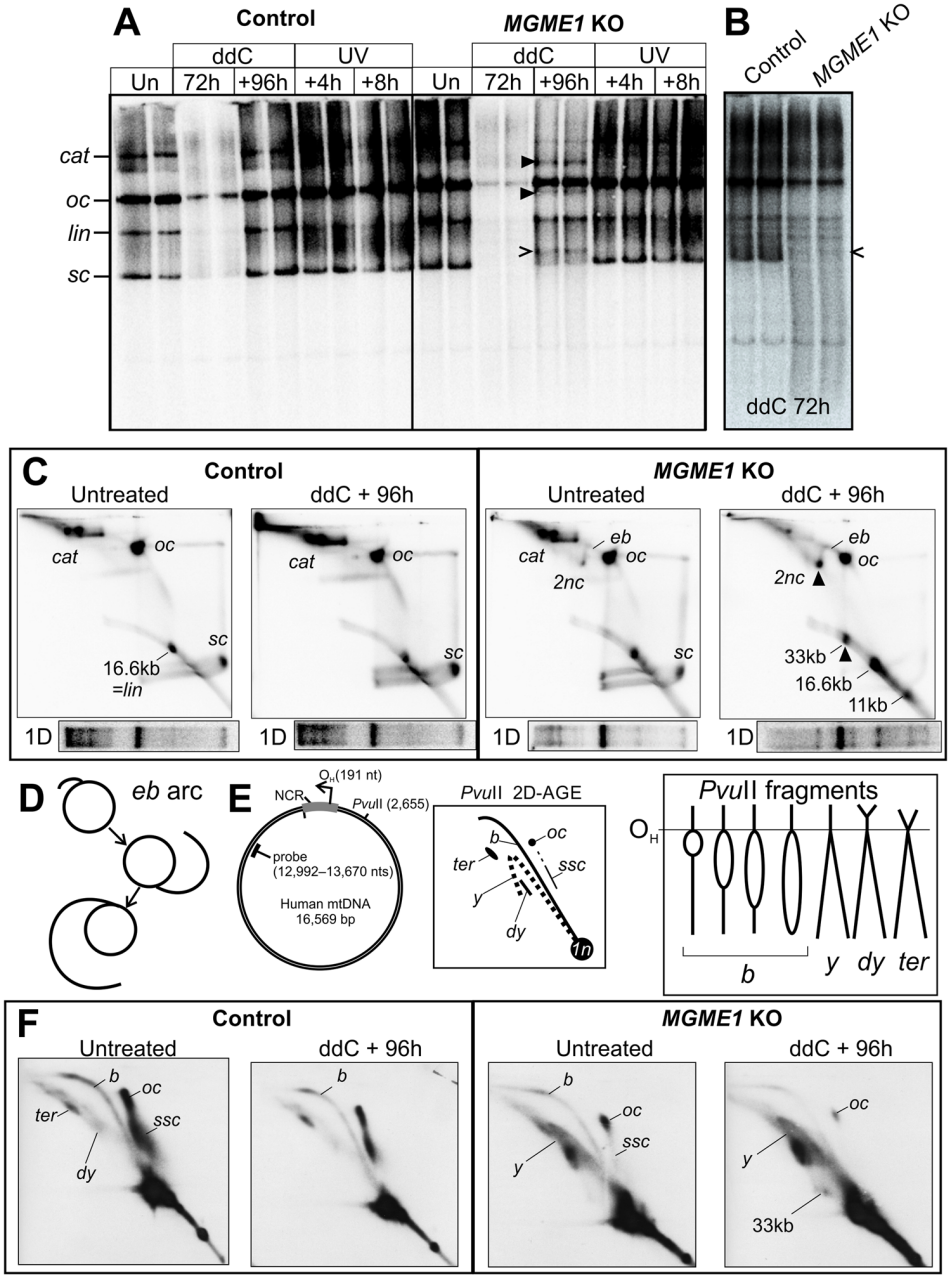
Replication stress causes an excess of linear forms and results in topological modification of mtDNA. (**A**) Topology gel of undigested mtDNA from control and *MGME1* knockout (KO) cells treated with ddC or UV; treatments as in Fig. 2. Under normal conditions, mtDNA in HEK293T cells exists as high molecular weight forms, including catenanes of two interlocked circles (*cat*), open circular monomers (*oc*) and supercoiled monomers (*sc*). Some mtDNA is linearized (*lin*) either naturally or due to extraction procedures. For the assignment of the different molecular forms see Pohjoismäki *et al*.^34^ and Supplementary Fig. S4. While ddC or UV treatment do not have a major influence on the different forms in control cells, *MGME1* knockout (KO) cells recovering from ddC exposure have novel molecular forms (black arrowheads) in addition to the 11-kb linear fragment (open arrowhead). (**B**) Long exposure of a separate re-run of the 72 h ddC samples. Note the excess smearing in the *MGME1* knockout cells, probably due to mtDNA breakage. (**C**) 2D-AGE of undigested mtDNA from untreated and ddC-recovering parental and *MGME1* knockout cells. The one-dimensional gel pattern from (**A**) is given below each panel to provide an approximate landmark. Notice the marked increase in linear forms in *MGME1* knockout cells recovering from the ddC treatment. The additional bands marked with black arrowheads in (**A**) represent dimeric circular forms (*2nc*) and 33 kb dimeric linear mtDNA. An unusual eyebrow-arc (*eb*) is present in the *MGME1* knockout cells, being more prominent in the cells recovering their mtDNA levels. (**D**) As the *eb* begins from the open circles and grows as an arc to the *2nc*, it most likely represents replication intermediates broken at one end. (**E**) These broken intermediates are visible on 2D-AGE of mtDNA fragments cut once upstream of O_H_, such as a *Pvu*II digestion produces. Most replicating forms in *Pvu*II -digested mtDNA should consist of replication bubbles (*b*), converting to double-*y* (*dy*) and termination (*ter*) intermediates close to the end of the molecule. If the replication bubble is broken at one end, the resulting intermediates will migrate on the y-arc (*y*). Note that some recently replicated circular forms are not cut by *Pvu*II, because they either are partially single-stranded (*ssc*) or have RNA incorporation at the restriction site (*oc*). For further details regarding these forms and their connection to mtDNA replication, see Torregrosa-Muñumer *et al*.^22^. Again, the majority of the molecules are non-replicative and linearized (*1n*) by the restriction enzyme. (**F**) While the control cells have almost solely full-length replication bubbles, *MGME1* knockout cells have mainly *y*-form replication intermediates, consistent with frequent breakage of the replication bubbles (see also Supplementary Fig. S5). The difference in the abundance of the circular forms (*oc*, *ssc*) between the wild-type and MGME1 knockout is likely caused by fewer molecules completing replication due to the premature arrest and breakage of the replication intermediates, resulting in depletion of the newly replicated molecules.

**Figure 4:**
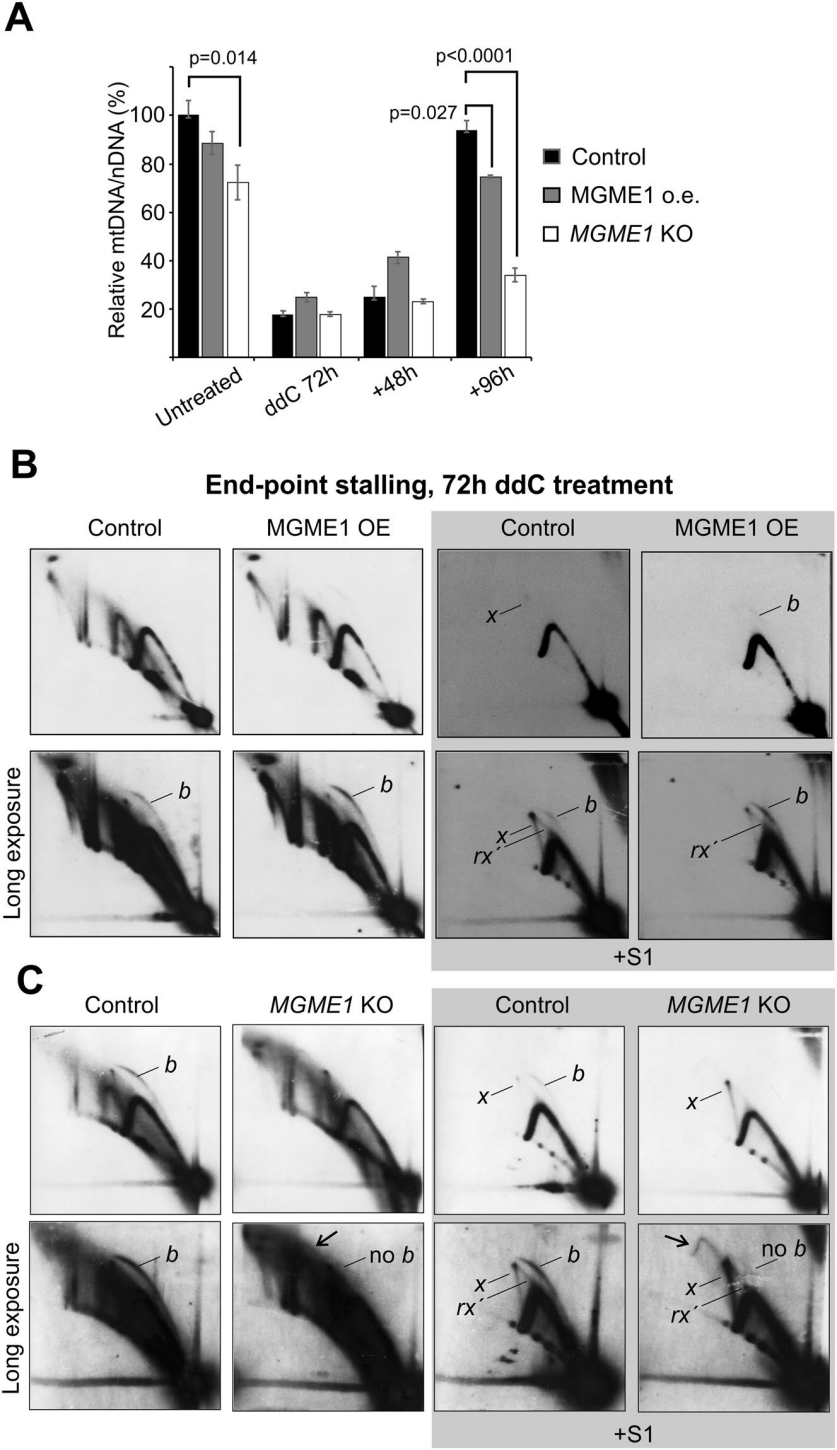
mtDNA copy number recovery is impaired by the loss of MGME1. (**A**) 72 h ddC treatment of HEK293T, MGME1 overexpressor and *MGME1* knockout cells, followed by recovery for 48 and 96 h. As evident from the severe copy number depletion after 72 h, ddC treatment results in an almost complete block of mtDNA synthesis. Parental HEK293T cells recover their mtDNA copy number to normal levels within 96 h after the removal of the drug, whereas the recovery is severely impaired in the *MGME1* knockout (KO) cells. All samples are normalized to the untreated HEK293T mtDNA copy number (100%) to show the differences in basal mtDNA levels. Results are presented as mean ± SD (*n* = 3, from at least two independent experiments) and *p* values based on one-way ANOVA with post-hoc Tukey HSD Test. (**B**) 2D-AGE analysis of the *Dra*I 12,273–16,012 fragment from ddC treated (200 µM ddC for 72 h) parental 293 T-REx cells (control) and MGME1 T-REx cells induced with 5 ng/ml doxycycline. (**C**) Identical analysis for regular HEK293 cells (control) and *MGME1* knockout (KO) cells. Treatment with S1 nuclease (right panels) reveals the fully double-stranded intermediates. The lower panels represent longer exposures of the upper ones and the exposures between adjacent panels are comparative. The treatment stalls all mtDNA replication intermediates and induces replication initiation outside of O_H_ (*b*, see Fig. 1) in all cells except *MGME1* KO. Regressed forks (*rx´*) appear different in the ddC treated cells compared to the UV (*rx* in Figs 1 and 2) and merge with the *x*-arc. Note the lack of a bubble arc, the prominent *x*-forms and an unusual arc (arrow) arising from *x*´s in the *MGME1* knockout cells.

To see if the *MGME1* knockout had any effect on mtDNA topology, we analyzed native undigested mtDNA using 1D- and 2D-AGE (Fig. 3, Supplementary Fig. S4). Again, the most prominent differences between parental HEK293T and knockout cells were observed in the cells recovering from ddC. *MGME1* KO cells had high levels of linear mtDNA forms, including a 33 kb species corresponding to dimeric mtDNA in size (Fig. 3C). Additionally, novel “eyebrow” forms (*eb* in Fig. 3C) were also observed (Fig. 3D). These forms start from the open circular species (*oc*) and form an arc that is growing in size in the first dimension, thus behaving similarly as replication intermediates. Further analysis of the non-linear forms revealed that in contrast to the control cells showing bubbles and circles, the *MGME1* KO cells had mainly *y*-shaped replicative molecules (Fig. 3E,F), which arise from broken replication forks with one end attached to O_H_ or O_L_ (Supplementary Fig. S5). Although the *MGME1* knockout had a relatively small (25% reduction) effect on steady-state mtDNA levels under normal conditions, the cells were nearly unable to regain normal mtDNA copy number levels after a ddC treatment (Fig. 4A). Concomitant with the failure to recover mtDNA copy number, *MGME1* knockout cells were unable to increase replication initiation from O_H_ in a similar manner as the wild type cells, as seen by the absence of a bubble arc in the observed fragment (Supplementary Fig. S6). In addition, replication initiation from alternative origins as a response to stalling was abolished in *MGME1* KO cells (Fig. 4B,C), as also seen after UV treatment (Fig. 1D).

**Figure 5:**
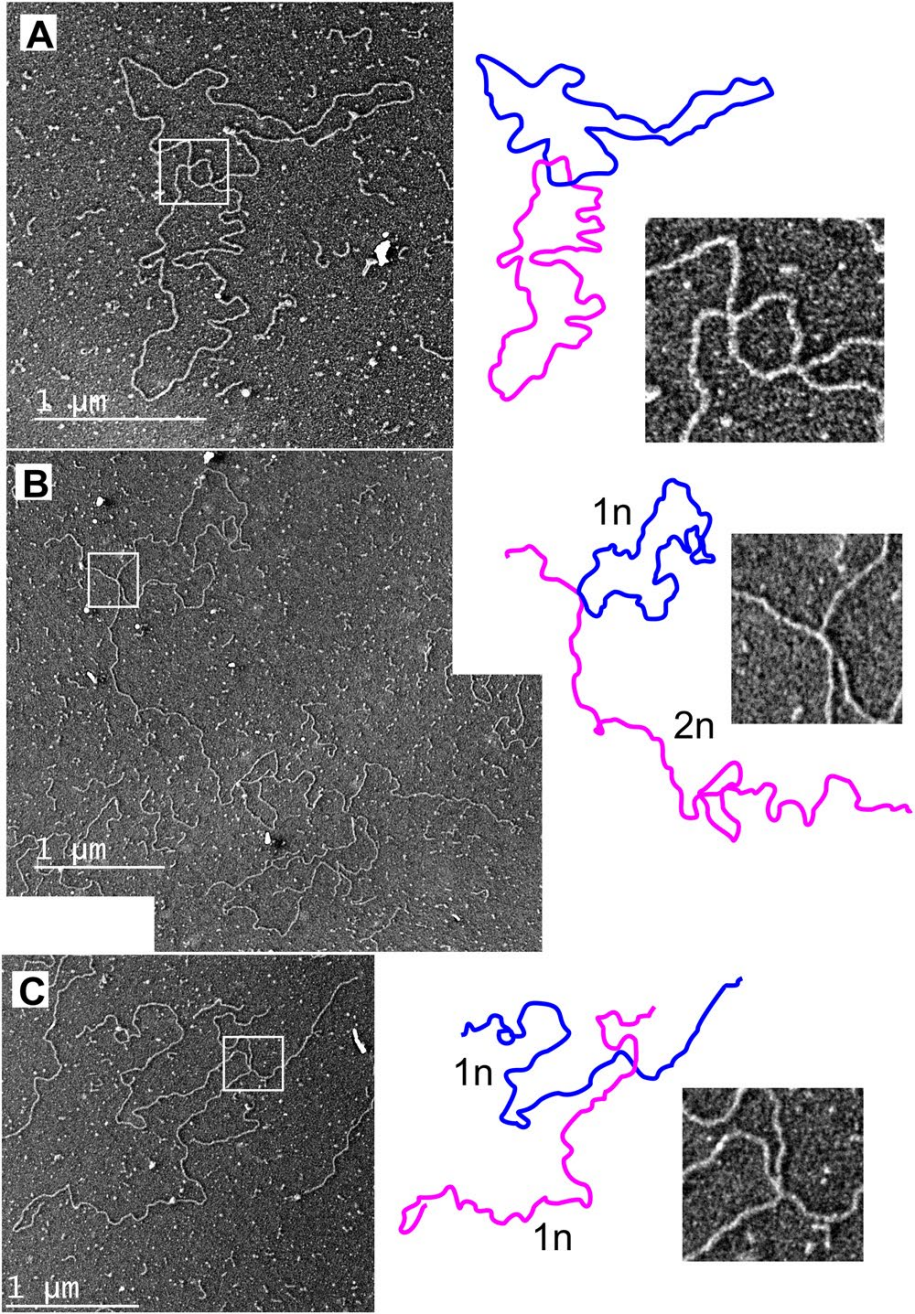
Examples of putative recombining molecules in *MGME1* knockout cells. Image interpretation and detail of the boxed area given in panels on the right. (**A**) 72 h ddC treatment of *MGME1* KO cells resulted in marked increase in complexes including two or more mtDNA molecules (see Table 1 for quantifications). (**B**) Dimeric (2n) linear molecule attaching to a monomeric (1n) circle with a cruciform junction. The picture is a montage of two separate TEM images because of the size of the molecule. (**C**) Two 16.6 kb linear molecules attached to each other with a cruciform junction. The adjacent arms of the molecule are equally long, indicating that the junction is at a homologous locus.

**Figure 6:**
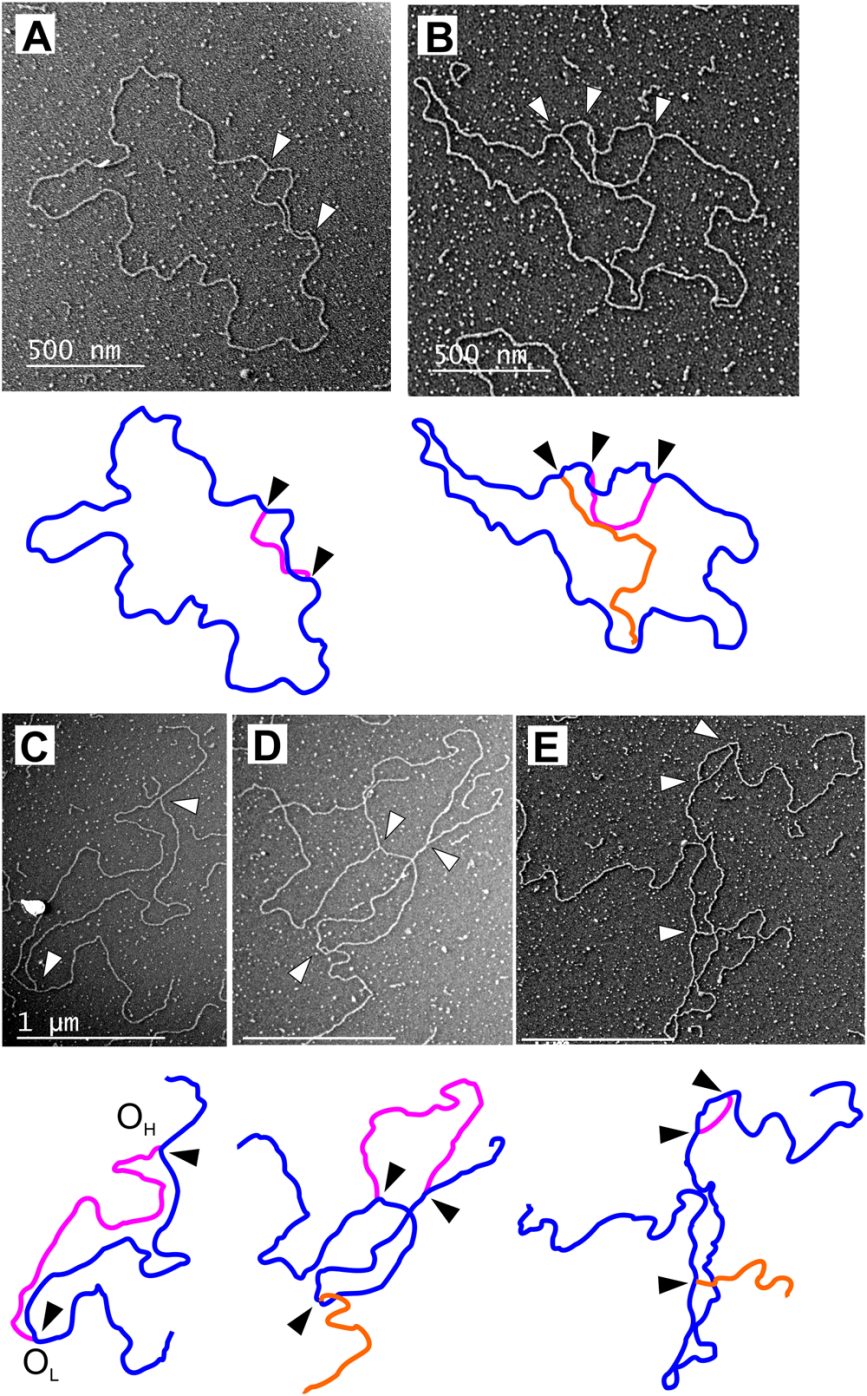
Evidence for replication fork rescue by converging replication in mammalian mitochondria. Interpretation of the molecule below each image. Arrowheads indicate replication forks. (**A**) The 72 h ddC treatment results in accumulation of replicating molecules in regular HEK293T cells, with the majority appearing as conventional replication bubbles. (**B**) A molecule containing a broken replication intermediate (orange) in addition to a replication bubble. (**C**) *Pvu*II -digested mtDNA from the same cells showing a linearized molecule with a replication bubble. Assuming the ends to match the *Pvu*II cut sites (see Fig. 3D), the replication forks map to O_H_ and O_L_ respectively. (**D**) Replication bubble containing an additional broken replication intermediate. Scale bar 1 µm. (**E**) Replication bubble from replication initiating outside of O_H_ on the same molecule with a broken intermediate. Scale bar 1 µm.

2D-AGE is a powerful tool to achieve a snapshot of all molecular forms present in a sample, but is dependent on the correct interpretation of the observed patterns. To consolidate our findings, we analyzed the mtDNA from untreated and 72 h ddC treated wild type HEK293T as well as *MGME1* knockout cells using transmission electron microscopy (Table 1). As expected, forced replication during recovery after ddC treatment caused a marked increase in replicative forms resulting from replication stalling, with a much stronger influence on *MGME1* knockout cells (8% vs. 19% replicative forms, Table 1). The majority of these molecules were broken at one end of the replication bubble (Supplementary Fig. S7), as already evident in the 2D-AGE analysis (Fig. 3C–F, Supplementary Fig. S5). Although these molecules might have been broken during sample preparation, it is highly unlikely that the breakage would mainly affect the *MGME1* KO cells (compare also the difference in the linear arc between control and knockouts in Fig. 3C). Concomitant with our observations using 2D-AGE, the *MGME1* KO cells also had higher levels of complex mtDNA forms containing two or more molecules (Fig. 5A, Supplementary Fig. S8), representing either recombining or catenated DNA, which also increased under ddC treatment (Table 1, Supplementary Fig. S4). Although linear mtDNA fragments are impossible to differentiate from contaminating nuclear DNA, we observed linear molecules corresponding to the size of dimeric mtDNA complexed with circular molecules in ddC treated *MGME1* knockout cells (Fig. 5B, see also Fig. 3C) as well as cruciform molecules (Fig. 5C). Other odd molecules observed from *MGME1* KO cells included several circular forms of about 6 kb in size (Supplementary Fig. S8). Interestingly, in parental HEK293T cells ddC treatment induced a specific molecular class with two replication events in one molecule (Fig. 6), likely corresponding to the observed increase in non-conventional replication bubbles under replicative stress (Figs 1, 4, Supplementary Figs S2 and 3).

**Table 1:** Mitochondrial DNA molecule types observed in the transmission electron microscopic (TEM) analysis of parental HEK293T and MGME1 knockout (KO) cells, without or with 72 h ddC treatment.

	HEK untreated	HEK ddC	*MGME1* KO untreated	*MGME1* KO ddC
*N*	400	400	400	100
Catenated or recombining	9%	15%	11%	29%
Dimeric circles	0.1%	0.2%	1.3%	ND
Replicating	5%	8%	5%	19%
of which broken	10%	40%	57%	32%

*N* = number of counted molecules. Note that catenated and recombining circles cannot be distinguished from the TEM imagery. Only 100 molecules were counted from the ddC treated *MGME1* KO due to high levels of linear mtDNA in these samples and therefore rare dimeric molecules were not detected (ND).

## Discussion

**Figure 7:**
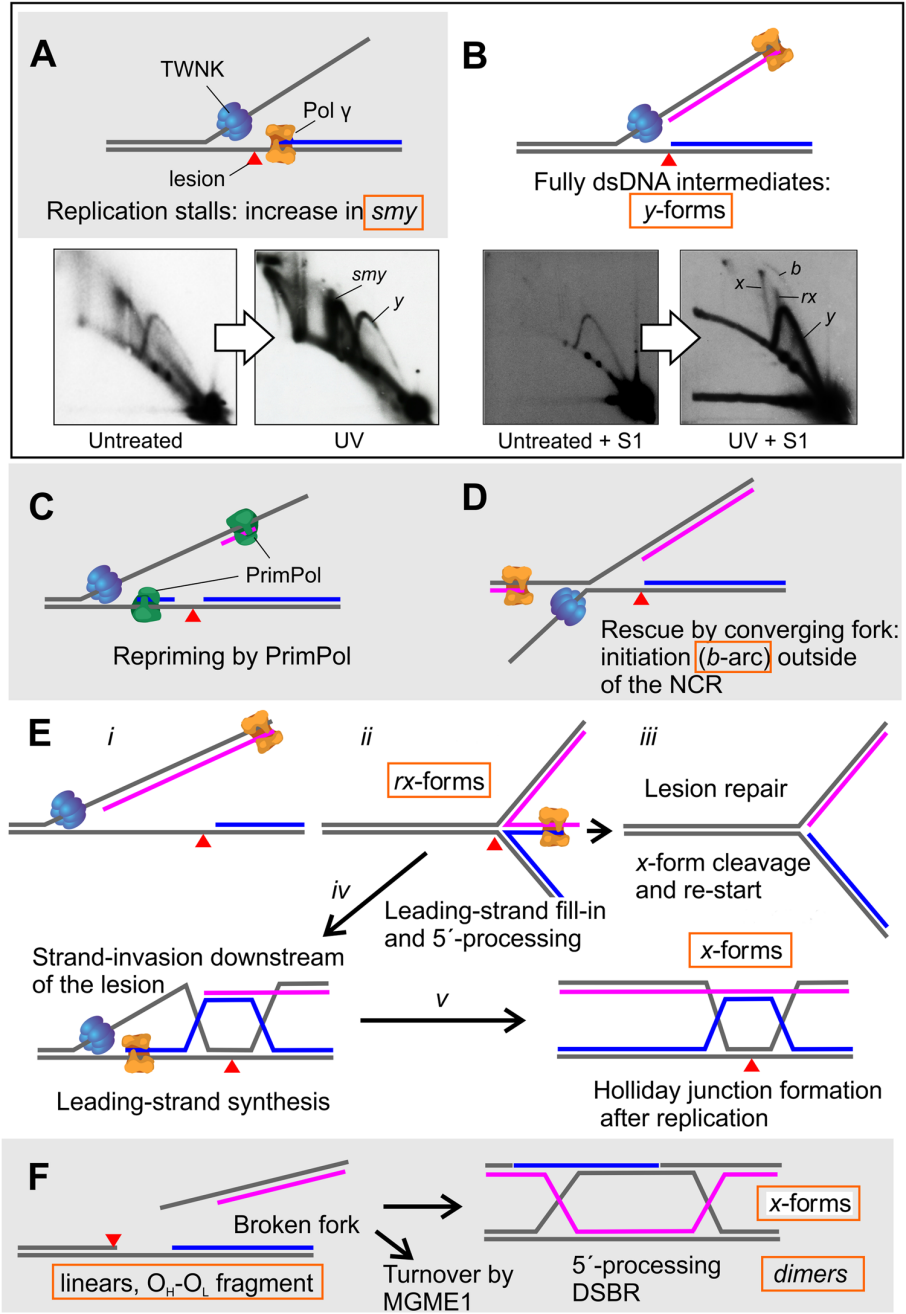
Possible mtDNA fork rescue mechanisms as interpreted from existing data. (**A**) Pol γ stalls at a lesion (red triangle) on the leading strand template. In cultured cells, stalling of the leading strand (marked in blue) results in accumulation of partially single-stranded (ss)DNA replication intermediates, increasing the abundance of the *smy*-forms seen as in the UV- treated control cells. (**B**) Stalling of the leading strand enables the lagging strand synthesis (pink) to catch up with the replication fork, resulting also in increase of fully double-stranded, S1 nuclease resistent replication forks (*y*-forms). (**C**) TWNK continues unwinding and PrimPol re-primes the leading strand synthesis downstream of the lesion^22^. PrimPol also frequently primes the lagging strand synthesis, contributing to the fully dsDNA replication intermediates. Consequently, the accumulation of dsDNA *y*-forms at the expense of *smy* is especially pronounced when TWNK helicase is stalled (Supplementary Fig. S3), as also the lagging strand synthesis is halted. (**D**) Replication initiation from unconventional origins could initiate the rescue of the stalled forks by convergence, giving rise to replication bubbles (*b* in B, Fig. 6) outside of the canonical origins. (**E**) If the leading H-strand replication remains stalled for longer periods, L-strand replication can bypass it. In other systems, fork regression is initiated by peeling back the lagging strand (*i–ii*), enabling the filling in of the stalled leading strand using the nascent lagging strand as a template and resulting in fork stabilization to a chicken-foot structure (*ii*). If the blocking DNA lesion is repaired, the regressed part of the fork is cleaved and the fork re-started from the leading strand 3′-end (*iii*). Alternatively, recession of the 5′-end of the filled-in fork will generate a free 3′-tail that can be used for homology-driven strand-invasion upstream of the lesion (*iv*), eventually resulting in Holliday-junction formation once replication is completed (*v*). (**F**) In cultured cells broken forks are mostly degraded in an MGME1-dependent manner. However, double-strand breaks can also be repaired by homology-dependent replication after 5′-end recession. The 3′-overhang at each end of the broken molecule will strand-invade an intact template and replicate the missing piece (break-induced replication)^23^. A Holliday-junction (*x*-form) will form once the replication is completed. Cleavage of Holliday-junctions can generate both monomeric as well as dimeric molecules (Figs 3, 5 and S4).

Replication stress or global impairment of DNA replication^2^ results in the transient slowing down or stalling of replication forks. In mitochondria, this has been suggested to be a major contributor for pathological mtDNA rearrangements^12,15,16,43^. Perturbations in replication processes have an obvious impact on mitochondrial genome maintenance, especially due to the mainly strand-asynchronous manner of mtDNA synthesis^17^. Damage on the leading strand template will stall the DNA polymerase^9^, resulting in the accumulation of strand-asynchronous replication intermediates (*smy*, Figs 1 and 7A)^8^. If leading strand stalling persists or also the helicase progression is halted^12,13,34^, lagging strand synthesis can catch up with the replication fork (Fig. 7B), converting mtDNA strand-asynchronous replication intermediates to fully duplex DNA intermediates. If the replication block only affects the DNA polymerase, the replicative helicase can continue to unwind the DNA duplex beyond the lesion, allowing the by-pass by a re-priming event (Fig. 7C)^22^.

If the replicative helicase (Supplementary Fig. S3)^12,13^ or the synthesis of both strands is stalled, as in the case of ddC treatment (Figs 2–4), re-start is no longer an option. In the nucleus such stalled forks can be rescued by converging forks from replication origins nearby (Fig. 7D), where a subset of replication origins are activated only during replication stress conditions^44^. Interestingly, also in mitochondria replication stress results in the appearance of replication bubbles outside of the canonical replication origins (*b* in e.g. Figs 1, 4 and 7B, Supplementary Figs S2 and 3)^8^ and in mtDNA molecules having two replication events (Fig. 6). As replication stress seems to specifically cause replication initiation outside of NCR, mitochondrial must possess mechanisms to license and prime alternative replication origins, which enable the completion of replication if the stalled fork cannot be resolved by other means. In bacterial systems, replication initiation outside of the canonical origins is primarily controlled by a RecBCD complex -mediated strand-invasion event^45^. As MGME1 is a RecB-type exonuclease^39^ and the loss of MGME1 activity abolishes stress-induced replication initiation (Figs 1D and 4D), existence of similar mechanisms in mitochondria should be investigated. This type of mechanism would also have considerable flexibility as it would not be dependent on the existence of specific origin sequences. Interestingly, the first step of the RecBCD -dependent replication initiation is the formation of a displacement loop (D-loop)^46^, a prominent but enigmatic feature of also mammalian mtDNA, that is modified in MGME1-deficient cells^40^.

Our observations on stress-induced replication could also explain the biological significance of replication initiation outside of the canonical origins, which have been known for over a decade^24,26,47^, but whose significance has been heavily disputed^16,21,48^. In contrast to replication initiating at O_H_, replication from the stress-induced origins is bidirectional and strand-coupled, as evident from the S1 nuclease-resistant bubble arcs that span the entire restriction fragment length in 2D-AGE analysis (Figs 1, 4 and 7B, Supplementary Figs S2 and 3). As pointed out before, this type of replication can be confused with frequent lagging-strand priming^49^, which also occurs under stress conditions^22^. Due to this similarity, separation of the two mechanisms is technically challenging. Similarly, nothing is known about the replication origin licensing or initiation in mitochondria and the experimental interrogation of the different origins is currently impossible. The fact that overexpression of TWNK can effectively repress this alternative origin initiation in mitochondria (Supplementary Fig. S3A) could simply indicate that excess helicase availability can forcibly push the stalled fork forward, allowing re-priming to take place.

If a stalled fork is fully duplex DNA, as expected once the lagging strand synthesis has caught up with the leading strand, the fork can be rescued by a mechanism involving fork regression and restart^1,2^. Fork regression results in a four-way junction – chicken foot – where the newly synthetized strands are annealed at the free end (Fig. 7E: *ii*). On 2D-AGE these structures migrate as typical spikes or cones originating from the *y*-arc^31,32^, and these spikes/features (*rx*) appear also in mtDNA after damage (Figs 1, 4 and 7D). As of note, *x*-forms are sensitive to branch migration and more likely lost than generated as an artefact during the DNA extraction (Supplementary Fig. S9). In the nucleus, the RAD51 recombinase is required for the first step of fork regression^50^, while bacterial systems use helicases such as RuvAB and RecG^51^.

TWNK helicase has been shown to possess strand-exchange activity *in vitro*^52,53^ and to be able to induce mtDNA recombination *in vivo*^35^. Interestingly, TWNK overexpression reduced the regressed forks (*rx*) and recombination junctions (*x*) during replication stalling (Supplementary Fig. S3A). This is in contrast to the effects in some solid tissues, where *x*-forms are specifically induced by TWNK overexpression^29,35^. It should be noted that this phenotype in transgenic mice is restricted only to tissues having mainly dsDNA mtDNA replication intermediates such as skeletal muscle and heart, indicating a fundamental difference in the mtDNA maintenance strategies in different tissues^28^. The effect observed in cultured cells is quite likely due to a more efficient lesion bypass instead of initiation of alternative origins (note the lack of *b* in Supplementary Fig. S3A). Alternatively, excess TWNK might increase the turnover of the regressed forks. In fact, TWNK has been shown to possess potent 5′ to 3′ DNA branch migration activity^54^, which might be required for the turnover of the *rx*-forms. In contrast, the expression of the catalytically impaired dominant-negative TWNK-LD did not suppress the appearance of the regressed forks, and the cells maintained replication initiation from alternative origins (Supplementary Fig. S3A). The experiment demonstrates that the helicase activity of TWNK is not required for fork regression (*rx*-arc), formation of recombination junctions (*x-*arc) or replication initiation from alternative origins.

In the nucleus, fork rescue continues with the repair and restart of the stalled fork by a homologous recombination-mediated mechanism^1,2^ (Fig. 7E). Fork regression will initially enable the nascent lagging strand to be used as a template to continue the leading strand synthesis, resulting in a chicken foot structure that stabilizes the stalled fork (Fig. 7E: *i* – *ii*). If the cell is unable to repair the damage on the leading strand template, the 5´-end at the chicken foot structure is enzymatically degraded, resulting in a free 3´-end that can strand invade upstream of the lesion (Fig. 7E: *iii* vs. *iv*). The initial strand-invasion event will eventually form true Holliday-junctions (*x*-forms) after the replication has finished (Fig. 7E: *v*). As of note, the required strand-invasion step is in principle identical to the replication template switching observed to occur upon double-strand breaks in human mitochondria and highly similar to the replication-dependent break repair seen in T7 phage^16^. In T7 phage, the gp2.5 single-strand DNA binding protein is responsible for the strand-invasion step^55^, whereas in mammalian mitochondria this reaction might be catalyzed by TWNK^52,53^, independently of its helicase activity. Molecular recombination is also the main rescue mechanism of broken replication intermediates and an important genome maintenance mechanism from bacteria to vertebrates^56^ (Fig. 7F).

The accumulation of linear subgenomic fragments, corresponding to broken replication intermediates, in patients with an MGME1 loss-of-function mutation^39,40^ or knockout mice^38^ are likely a consequence of two independent events. They are probably generated by defective ligation of nascent strands at the origin due to faulty processing of DNA flap structures^42^ and then accumulate due to the inability of MGME1-deficient cells to degrade linear mtDNA fragments^37^. We show these molecules to arise from site-specific stalling and breakage at either one of the main replication origins (Supplementary Fig. S5), resulting in tailed intermediates (Fig. 3D–F, Supplementary Fig. S7) that eventually break to form the linear 11 kb fragment.

The fact that MGME1 overexpression resulted in a decrease of all *x*-forms after mtDNA damage (Figs 1D and 3–5) could be linked to an increased turnover of linear mtDNA. The free end of a regressed fork is essentially a double-strand break and its rapid turnover in MGME1 overexpressor cells might result in a repair defect, which could explain the delayed recovery from ddC treatment (Fig. 4A). Conversely, impairment of linear DNA disposal in the *MGME1* knockout cells increases the *x*-forms (Figs 1D, 4 and 5, Supplementary Fig. S4, Table 1), likely indicating an active double-strand break repair. This observation suggests that cells primarily try to discard broken mtDNA, but if this does not work, homology dependent repair is used as a reserve mechanism. The importance of the two mechanisms likely varies between cell types, explaining the abundance of *x*-forms in some tissues but not in others^28,29^. As of note, even untreated *MGME1* KO cells have a specific class of dimeric molecules, which are sensitive to the cruciform-cutting endonuclease T7gp3, but not to the decatenating Topoisomerase IV enzyme (Supplementary Figs S4 and S9). Interestingly, both T7gp3 as well as S1 nuclease can convert more complex *MGME1* KO mtDNA forms into a 33 kb linear species, further demonstrating that these genomic dimers are generated by recombination in mitochondria, as previously suggested for the human heart mtDNA forms^29,35^.

While it is impossible to pinpoint to which extent these recombining forms arise from fork rescue (Fig. 7E) or double-strand break repair (Fig. 7F), it is reasonable to assume that both mechanisms work in mitochondria. First, replication stalling *per se* results in fork regression and *x*-formation in UV treated cells (Fig. 1) without increasing the levels of the O_H_–O_L_ fragment (Fig. 2B). Secondly, *MGME1* KO cells have high basal levels of *x*-forms (Fig. 1D), correlating with linear fragments (Fig. 2), dimeric mtDNA (Fig. 3C) and junctional molecules (Fig. 5), all consistent with double-strand break repair by recombination. The dimeric mtDNA forms are mostly head-to-tail dimers, as their cleavage with a single-cut restriction enzyme results in 16.6 kb linears (Fig. 3C vs. 3F). Similar rearrangements, including unicircular dimers to pentamers and strand-invasion intermediates, have been reported from cells expressing catalytically impaired TWNK helicase^43^, suggesting a common mechanism for their formation through replication stalling-induced double-strand breaks.

The fact that the O_H_–O_L_ fragment appears transiently also in control cells as a result of replication stalling or when mtDNA replication is boosted to recover the copy number (Fig. 2), is interesting. First, the type and persistence of the damage seems to be important. Although UV treatment causes a distinct replication stalling phenotype, it does not influence mtDNA copy number (Supplementary Fig. S10) or the abundance of the O_H_–O_L_ fragment (Fig. 2B), probably due to the transient nature of the replication stalling compared to the ddC treatment, which leaves blocked 3′-ends that require an exonuclease for repair. Secondly, the increase in the sub-genomic fragment during mtDNA copy number recovery after ddC treatment demonstrates that O_H_ and O_L_ are frequent breakpoints also under normal conditions and that even normal mtDNA replication could contribute to double-strand breaks and subsequent deletion formation. In fact, rearrangement hotspots around O_H_ are well known both from mice^57,58^ and humans^40,59^, further indicating that this replication origin is an important fragile site on mtDNA. Our experiments might also give cues how mtDNA deletions rise during post-natal development in some tissues. Recovery from ddC-induced mtDNA depletion, characterized by rapid amplification of mtDNA to return to the steady-state copy number^25,34^, emulates physiological conditions where mtDNA levels increase as a response to mitochondrial biogenesis. A boost in mitochondrial biogenesis, such as during muscle development^60^, could generate a first generation of pathological deletion mutants, which get clonally expanded over time^15^. Furthermore, antiretroviral therapy using ddC or other similar nucleotide analogues has been shown to result in expansion of mutant mtDNA forms with deletions spanning between O_H_ and O_L_^61^, providing a potential link between replication fork breakage observed in this study and the generation of pathological deletions.

As a conclusion, our study demonstrates that mitochondria show similar responses to replication stress as the nuclear genome, including initiation of alternative replication origins and fork regression. Interestingly, linear mtDNA resulting from breakage of replication intermediates seems to be highly recombinogenic. An efficient MGME1-dependent pathway to dispose broken mtDNA molecules could therefore be a first line of genomic defense against pathological rearrangements. On the other hand, our results also suggest an important, perhaps tissue-specific role for recombination-dependent DNA repair in mitochondria. While the molecular mechanisms to overcome replication stalling are well known from other genetic entities, their elucidation in mitochondria, including the interdependence of *x*-forms and replication bubbles, remains a challenge for future research.

## Methods

An overview of the reagents, cell lines and key equipment used in the study is presented in Supplementary Table S1.

### Cell culture, ddC and UV light treatments

HEK293T and HEK *MGME1*-KO cell lines were cultured in DMEM containing 4.5 g/l glucose, 2 mM L-glutamine, 1 mM sodium pyruvate, 50 μg/ml uridine and 10% fetal bovine serum at 37 °C in a humidified atmosphere with 8.5% CO_2_ atmosphere. Flp-In^TM^ T-REx^TM^ 293 MGME1 wild type (MGME1 OE)^62^ and TWNK wild-type (TWNK OE) and linker-duplication (TWNK LD) mutant^13^ cell lines were cultured similarly, and 5 ng/ml of doxycycline was used to induce transgene expression (Supplementary Fig. S1). After the indicated times, cells were harvested and DNA samples were extracted. To induce mitochondrial DNA replication stalling, cells were treated for 3 days with 200 μM of 2′,3′-dideoxycytidine (ddC) (Sigma-Aldrich)^8^. For UV damage, cells were exposed on the tissue culture dish with DMEM to a single dose of 30 seconds of 1.34 mJ/cm2 x s UVB (302 nm), using a Benchtop 2UVTM transilluminator 302 nm (UVP) instrument^8^. UV light doses were controlled using a handheld UV radiometer (MINOLTA, UM-25).

### Generation of HEK293T MGME1 KO cell line

MGME1 knockout (KO) HEK293 cells were generated using the Crispr-Cas9 approach, as described previously^37^. The plasmid vector carrying a single guide RNA (sgRNA) sequence for MGME1 (5′-AGACCATTTGCAGGCAGCTC-3′) and coding for Cas9 were purchased from Origene. HEK293T cells were plated at a density of 10^4^ cells per 96-well and transfected with the plasmid using the GeneJuice transfection reagent (Novagen) on the following day. 48 hours after transfection, cells were resuspended and plated on 96-well plates at 0.5 cells/well concentration. After 2 weeks of growth, cells from single colonies were resuspended in 200 μl medium. Half the volume was transferred to a new well for further growth, while cells from the other half were centrifuged and resuspended in 20 μl lysis buffer (1 mM CaCl_2_, 3 mM MgCl_2_, 1 mM EDTA, 1% Triton X-100, 10 mM Tris pH 7.5) containing proteinase K (0.2 mg/mL, Qiagen). Incubation at 65 °C for 10 min was followed by heat inactivation at 95 °C for 15 min. Lysates were directly used for PCR amplification and subsequent genotyping by Sanger sequencing. The obtained MGME1 knockout clone was homozygous for the c.37_41del5 frameshift deletion and carried a 197-bp insertion from the cloning vector on one allele.

### Protein isolation and western blotting

To check protein expression of TWNK-WT-myc^13^, TWNK-LD-myc^12^ and MGME1-WT-flag^62^ constructs in the respective cell lines (TWNK OE, TWNK LD and MGME1 OE), cellular proteins were extracted as described^28^. 30–40 μg of total protein were separated by 8–12% SDS-PAGE and blotted onto nitrocellulose membrane (AmershamTM ProtranTM 0.2 μm, GE Healthcare). Detection of transgenic proteins was performed using anti-flag or anti-myc primary antibodies (Supplementary Table S1), HRP-coupled secondary antibodies and chemiluminescent detection with the BioSpectrum ® 810 imaging System (UVP).

### Total DNA extraction, mtDNA topology and mtDNA species determination by Southern blotting

Total cellular DNA was isolated using proteinase K and SDS lysis followed by phenol:chloroform extraction and ethanol precipitation^29^. DNA pellets were re-suspended overnight at 37 °C in the presence of FastDigest *Bgl*II (ThermoFisher), which does not cut mtDNA, and the DNA concentration was determined with a NanoDropTM1000 spectrophotometer. For mtDNA topology analysis, 2 μg of *Bgl*II digested total DNA were separated on a 0.4% agarose gel in 1 x TBE at 1.2 V/cm for 16 h. Southern blotting and DNA hybridization were carried out as previously^29^, using a PCR probe spanning nucleotides 12,992–13,670 (ND5). For mtDNA species (intact mtDNA, 11 kb linear fragment, duplication and 7 S DNA) determination, 2 μg of total DNA were digested with FastDigest *Afl*II and *Hind*III (ThermoFisher) for 6 hours at 37 °C, and then separated on a 0.8% agarose gel in 1x TAE buffer at 25 volts for at least 16 hours. For the visualization of 7 S DNA, DNA samples were partially denatured for 10 minutes at 65 °C before loading. Southern blotting was performed as before and the blot was probed with a 7 S probe spanning nts 12,992–13,670. Probes were labeled using the Rediprime™ II random prime labeling kit (GE Healthcare) and [α-32P]-dCTP (PerkinElmer; 3,000 Ci/mmol). The autoradiographs were captured on a KODAK storage phosphor screen SO230, detected using Molecular Imager FX (BioRad) and quantified using the associated QuantityOne software.

### mtDNA copy number determination by real-time quantitative PCR (qPCR)

mtDNA copynumber per cell was determined from total DNA using duplex Taqman quantitative PCR as described^28^ on an AriaMx Real-Time system (Agilent Technologies).

### Mitochondrial DNA isolation, two-dimensional agarose gel electrophoresis (2D-AGE) and Southern blotting

Mitochondrial DNA was isolated using 20 µg/ml cytochalasin (Sigma-Aldrich) treatment for 30 minutes prior to cell breakage, followed by differential centrifugation and sucrose gradient purification^63^. The 2D-AGE analysis was performed essentially as in Pohjoismäki *et al*. 2006^34^. Briefly, 10 μg of mitochondrial nucleic acid were digested according to the manufacturer’s recommendation with FastDigest *Dra*I (Thermo Scientific) and run on a 0.4% agarose gel in 1xTBE until the fragments of interest had migrated 10 cm into the gel. The gel slab was rotated 90° and a 0.95% agarose gel with 0.5 µg/ml ethidium bromide (EtBr) was cast around it. The second dimension was run in the presence of 0.5 µg/mL EtBr until the fragment of interest had migrated ~10 cm. Southern blotting and hybridization were performed as described before using the ND5 probe. Where indicated, partially single-stranded DNA was degraded by adding 50 units of S1 nuclease (Thermo Scientific) to the digested DNA samples and incubated for 5 minutes at room temperature prior loading the samples onto the gel. The long-range *Pvu*II 2D-AGE analysis was performed as in Torregrosa-Muñumer *et al*.^22^ Uncut long-range 2D-AGE analysis was performed with 10 µg of undigested total DNA, run as a normal long-range 2D-AGE and probed against ND5. The autoradiographs were exposed either on Kodak MS film (Sigma-Aldrich) or captured on a KODAK storage phosphor screen SO230 and detected using Molecular Imager FX (BioRad).

### Transmission electron microscopy (TEM)

Mitochondrial DNA was isolated as for 2D-AGE, treated with 50 units of RNase I (Thermo Scientific) at 37 °C for 30 min, subsequently purified using phenol-chloroform extraction and ethanol precipitation and dissolved in TEM grade TE buffer (10 mm Tris-HCl, 0.1 mm ETDA, pH 7.6). mtDNA preparation was visualized by EM following the Kleinschmidt procedure^64,65^. Briefly, 25–50 ng of mtDNA were diluted in 50 µl aqueous solution of 250 mM ammonium acetate and 100 µg/µl cytochrome c, mixed gently, and the drop allowed to develop for 15–20 min on parafilm. Cytochrome-coated DNA floating on the drop surface was captured by gently touching the drop with a parlodion-coated copper grid. The grids were rinsed in 70% and 85% EtOH solutions, 20 s. each. Following quick air-drying, the grids were placed in a high vacuum evaporator and a thin layer of platinum (80%): palladium (20%) was evaporated on the sample at an angle of 8 degrees at 2 × 10^−6^ torr. Finally, the grids were covered with a thin layer of carbon to stabilize the parlodion film. Samples were examined in an FEI T12 TEM equipped with a Gatan 2kx2k Orius CCD camera at 40 kV. Contour lengths of the molecules were measured using Digital Micrograph software. Monomeric mtDNA (16,569 bp) equals about 5,500 nm.

## Supplementary information

Supplementary data
