# Supplementary material for: Replication fork rescue in mammalian mitochondria
Source: Sci Rep. 2019 Jun 19;9:8785. doi: 10.1038/s41598-019-45244-6 (PMC6584726; doi:10.1038/s41598-019-45244-6)

Replication fork rescue in mammalian mitochondria

Rubén Torregrosa-Muñumer[1,2], Anu Hangas[1], Steffi Goffart[1], Daniel Blei[3], Gábor Zsurka[3], Jack Griffith[4], Wolfram S. Kunz[3] & Jaakko L. O. Pohjoismäki[1§]

[1] Department of Environmental and Biological Sciences, University of Eastern Finland, P.O. Box 111, 80101 Joensuu, Finland.

[2] Research Programs Unit, Molecular Neurology, University of Helsinki, Helsinki, Finland

[3] Department of Experimental Epileptology and Cognition Research, University of Bonn, Sigmund-Freud-Str. 25, Bonn, D-53105, Germany.

[4] Lineberger Comprehensive Cancer Center, University of North Carolina at Chapel Hill, USA.

[§]Corresponding author: Tel. +358-505744745; e-mail: Jaakko.Pohjoismaki@uef.fi

SUPPLEMENTARY DATA

Supplementary Materials and Methods

In-gel branch migration

To promote branch migration of mtDNA replication and recombination intermediates, gel lanes from the first-dimension electrophoresis were incubated for 4 h at 65°C in 1 x TBE electrophoresis buffer. Control lanes were incubated for 4 h at room temperature. Second dimension gel was cast and run as for the regular 2D-AGE.

Enzymatic treatments to reveal topological identities of different mtDNA forms

One µg of isolated mtDNA was treated with either 50U of S1 (Thermo Scientific) for 2 min at room temperature or with 0.5U of E. coli Topoisomerase I (New England Biolabs), 5U of *E. coli* Topoisomerase IV (Inspiralis), 10U of T7gp3 (T7 endonuclease I, New England Biolabs) endonuclease or with 5U of *E. coli* RNaseI (Thermo Scientific) for 30 min at 37˚C, using the supplied buffer and following the manufacturers' recommendation. The reaction was stopped by addition of phenol:chlorophorm:IAA (25:24:1, pH 8.0). The water phase was extracted by centrifugation, transferred into a new tube, mixed with loading dye and separated by agarose gel electrophoresis as described in the main text.

Supplementary Figures

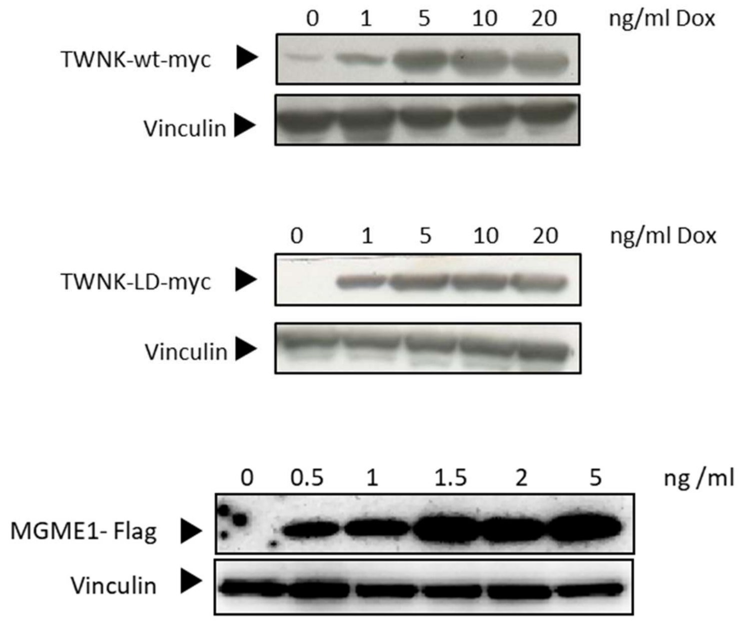

Supplementary Figure S1. Doxycycline (Dox) inducible expression of wild-type (wt) and linker-duplication (LD) TWNK as well as MGME1 in 293 T-REx cells.

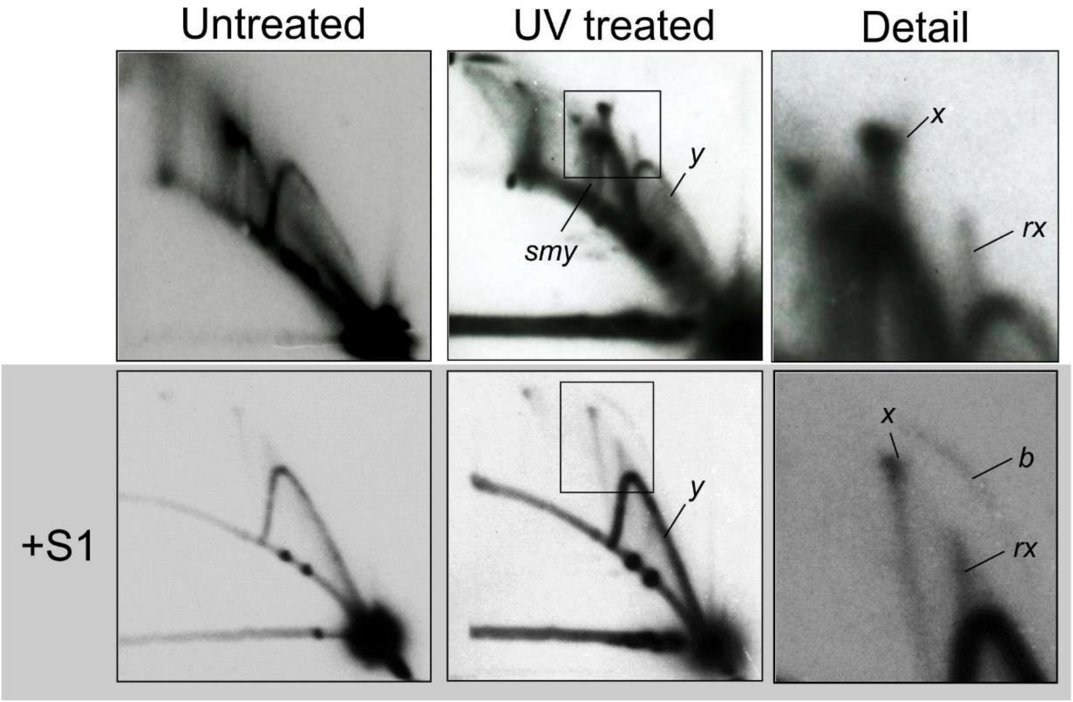

Supplementary Figure S2. Representative 2D-AGE panels of control HEK293T mtDNA before (untreated) and 4h after 30s exposure to 1.34 mJ/cm^2, 302 nm wavelength UVB (UV treated). Besides inducing an increase in *smy*-arc, UV exposure causes a specific increase in *x*- and *rx*-forms (boxed area in detail), as well as in replication bubbles (*b*) in this region of the genome. The simple *y*-forms, *x*'s, *rx*'s and *b*'s are all double-stranded DNA as they are insensitive to S1 nuclease digestion (lower panels).

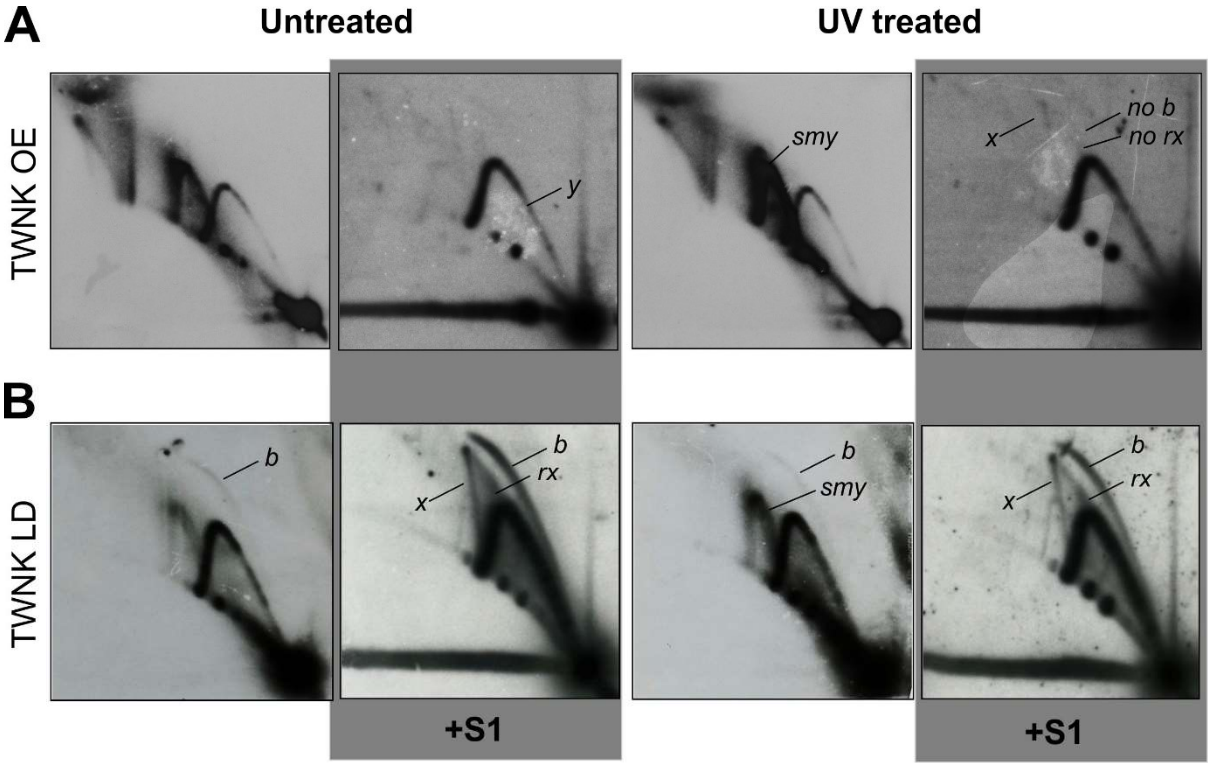

Supplementary Figure S3. 2D-AGE analysis of *Dra*I 12,273–16,012 fragment of mtDNA from TWNK overexpressing or LD mutant cells, untreated or UV treated as in Figure 1. Panels represent comparative exposures for each row. (A) Compared to the parental T-REx-293 cell line, TWNK overexpressing (OE) cells have more double-stranded DNA replication intermediates (*y*) than *smy*-forms. TWNK overexpression also completely abolishes fork regression (*rx*-forms) following UV treatment. Note the similar increase in *smy*-forms following UV treatment as in the control cells. (B) The dominant negative TWNK LD mutant exhibited a strong replication stalling phenotype, including regressed forks (*rx*), recombination intermediates (*x*) and a marked increase in replication initiation (*b*). While UV treatment did increase the *smy*-arc, its influence on dsDNA intermediates was negligible.

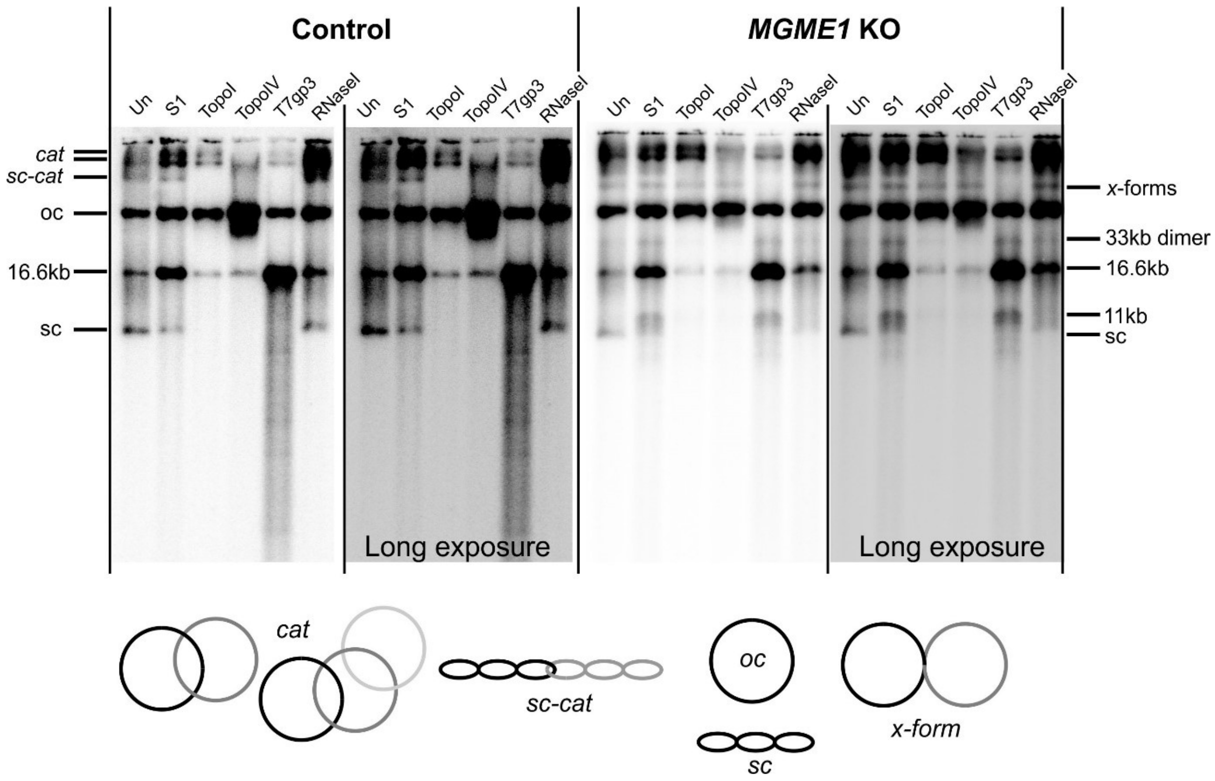

Supplementary Figure S4. Different topological forms of mtDNA in control (left lanes) and *MGME1* KO (right lanes) HEK293T cells. There are three distinct catenated (*cat*) forms, with the lowest (*sc-cat*) being Topoisomerase I (TopoI) sensitive and therefore supercoiled. Note also the disappearance of monomeric supercoils (*sc*) upon TopoI treatment. All catenanes as well as supercoils are sensitive to the decatenating Topoisomerase IV (TopoIV), but not to the cruciform-cutting T7gp3 endonuclease. T7gp3 does, however, nick and relax both supercoiled (*sc, sc-cat*) forms. Both the promiscuous single-strand cutting nuclease S1 as well as RNaseI cause linearization of mtDNA (16.6kb band), but do not have a specific impact on any of the molecular forms in control cells. *MGME1* knockout (KO) cells have an additional high-molecular weight mtDNA species (*x*-form), which is sensitive to T7gp3 but not to S1. As most mitochondrial replication intermediates have extensive areas of single-stranded DNA sensitive to S1 nuclease treatment, the *x*-forms must correspond to the abundant recombining forms seen the 2D-AGEs (Figures 1 and 4). Apart for generating 16.6kb linear fragments, both S1 as well T7gp3 also release the dimeric linear (33kb) as well as the O_H–O_L fragment (11kb), indicating that these molecular species are mostly incorporated into larger forms by replication or recombination junctions. Note that the gel run differs slightly from the gel in Figure 3, causing the high molecular forms being more compressed while having better resolution for the lower forms.

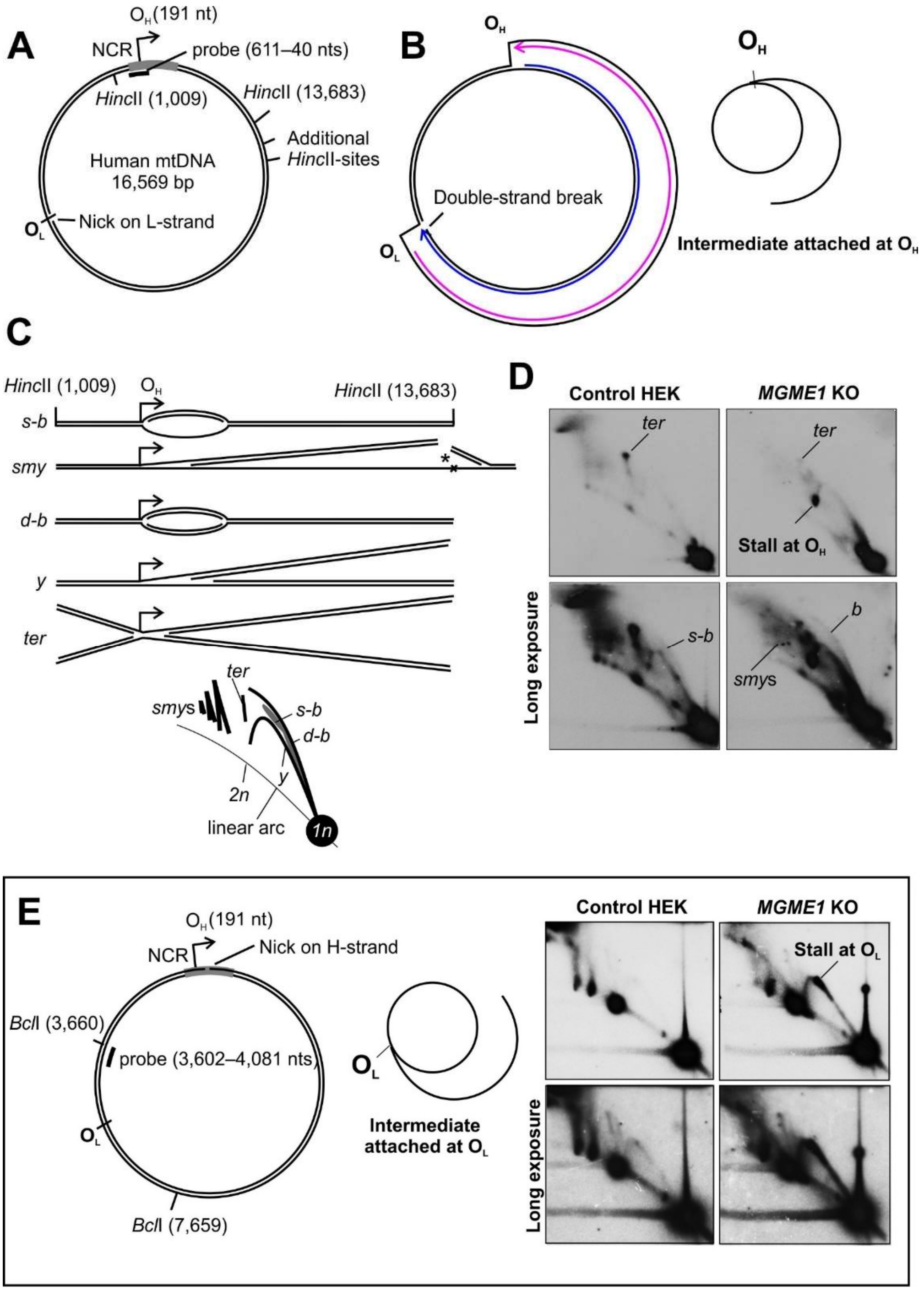

Supplementary Figure S5. Strong replication stalling at O_H indicates replication intermediates broken at O_L. (A) A schematic illustration of human mtDNA showing *Hinc*II cut sites near the non-coding region (NCR) and the probe location. (B) A nick at O_L due to ligation deficiency in *MGME1* knockout (KO) cells will generate a double-strand break when H-strand replication reaches it. If the broken replication intermediate remains attached at O_H for longer periods, this will appear on 2D-AGE as tailed (Figure 4C) or *y*-shaped (Figure 4F) molecules. (C) Replicating molecules associated with NCR containing *Hinc*II fragment of human mtDNA. Strand-displacement replication initiating at OH will give rise to partially single-stranded

replication bubbles (s-*b*), which convert to various slow-moving y's (*smy*) in the case of strand-displacement replication due to incomplete digestion (*) of the displaced strand (other downstream *Hinc*II sites omitted). The *s-b* bubbles differ from the fully double-stranded replication bubbles (*d-b*), being shorter and blunter. Double-stranded DNA replication intermediates originating from replication initiated outside of the NCR form *y*-shaped molecules terminating at O_H. Note that the y-arc is not complete (does not reach the linear arc) as O_H is located 800 bp from the end of the fragment. Once replication has progressed throughout the genome, it will terminate at O_H, resulting in x-shaped termination intermediates (*ter*). (D) *MGME1* KO cells show punctate accumulation of replication intermediates at the tip of the descending y-arc, corresponding to broken replication intermediates attached at O_H (see B and Figure 4). Termination intermediates (*ter*) are depleted, probably due to fewer replication events progressing through the entire genome. Interestingly, unlike elsewhere in the genome (Figure 2B, 5D), replication initiation at the NCR region is increased in the *MGME1* KO cells. Curiously, the replication bubbles seem to be fully double-stranded (*d-b*) in contrast to strand-displacement bubbles (*s-b*) seen in the controls.

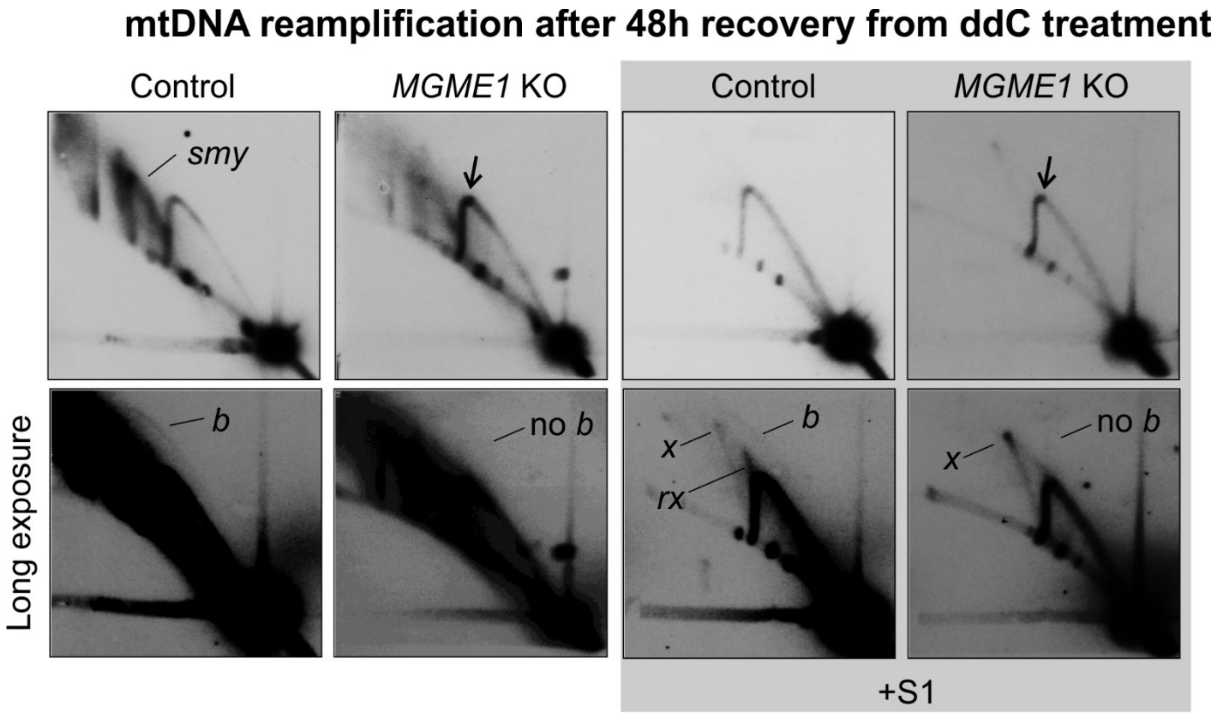

Supplementary Figure S6. 2D-AGE analysis of *Dra*I 12,273–16,012 fragment after 48h recovery from 72h ddC treatment (see Figures 1 and 4 for explanations). The control cells are replicating mtDNA mainly via the strand-asynchronous mechanism, as evident from the prominent *smy*-arc (see Figure 1 for details). Replication bubbles (*b*) are present in the control cells, but less prominent than after 72h ddC treatment. In contrast, the *MGME1* knockout cells have reduced *smy*-arc but an accumulation of the descending *y*-arc intermediates (arrow), corresponding to mild replication stalling. As shown before (Figure 4), the knockout cells lack a bubble arc but show high levels of *x*-forms.

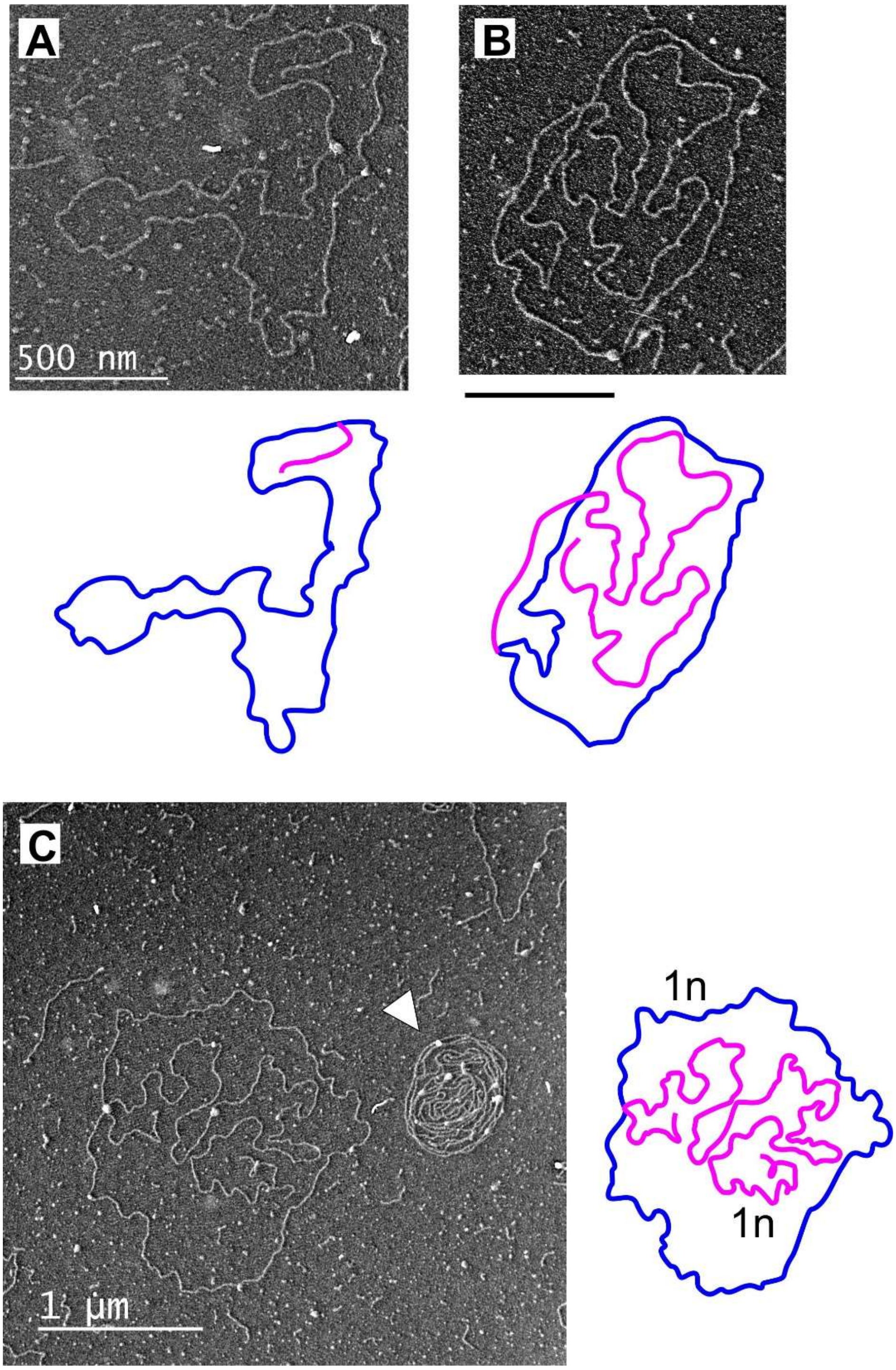

Supplementary Figure S7. Examples of broken replication intermediates from *MGME1* KO cells. Interpretations below/next to each image. (A) mtDNA molecule with a short tail. (B) mtDNA molecule with a long tail. Scale bar 500 nm. (C) mtDNA circle connected to a genome-length linear, likely presenting a recently replicated molecule broken at a distance from the replication forks. Arrowhead points to a DNA coil, sometimes observed in the TEM preparations.

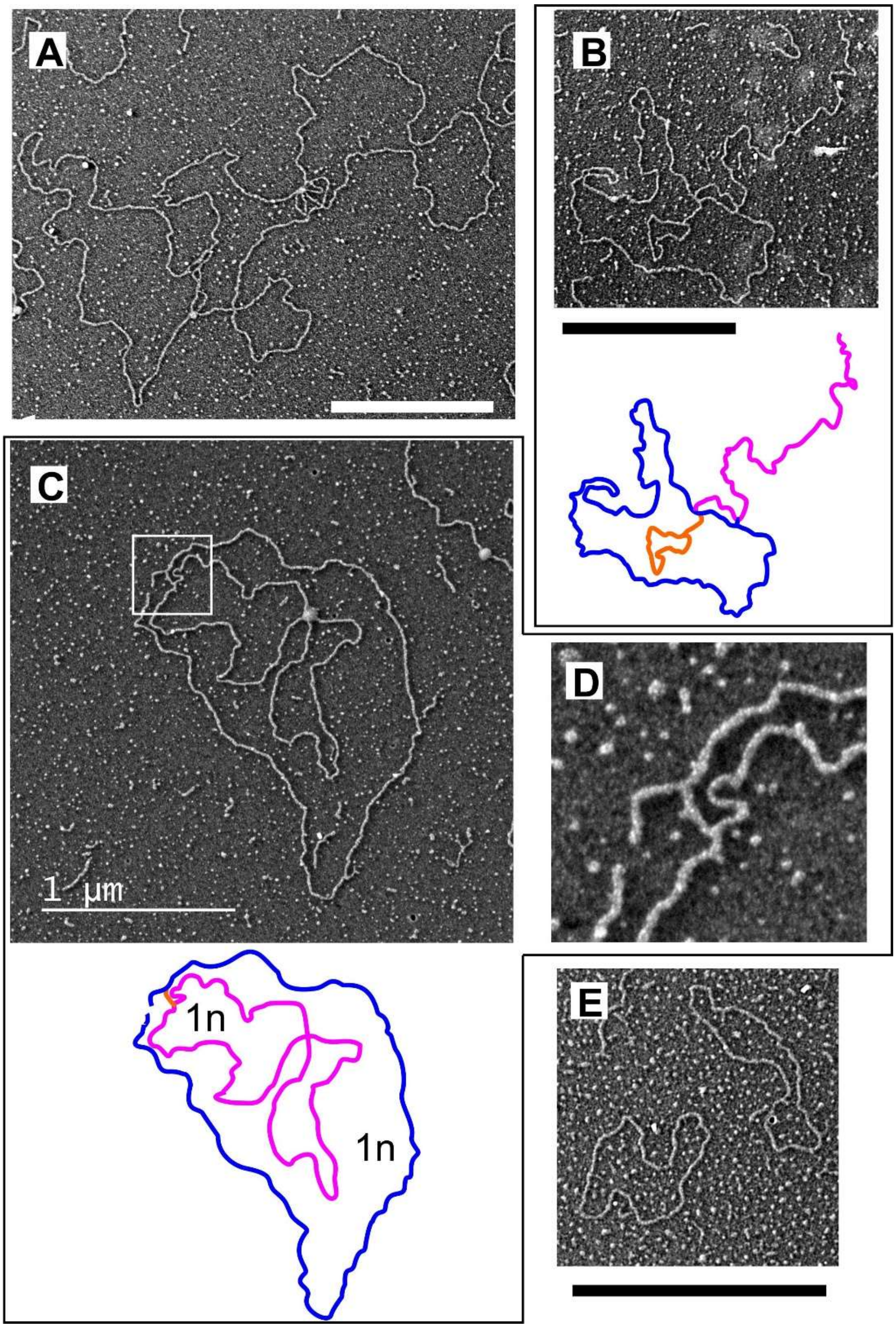

Supplementary Figure S8. Examples of non-conventional mtDNA forms. Interpretations adjacent to each image. (A) Three mtDNA circles connected with complex junctions from ddC treated HEK293T cells. Scale bar 2 µm. (B) A mtDNA circle with a tailed bubble structure and a tailed small circle from *MGME1* KO cells. Scale bar 2 µm. (C) Looped circular mtDNA (drawn in pink) surrounded by a 16.6 kb linear molecule (blue) from ddC treated HEK293T cells. Note the connecting short DNA strand between the two molecules (orange). (D) A close-up of the boxed area in (C) showing the connecting strand in detail. (E) Examples of 6 kb small circles found from *MGME1* KO cells. Scale bar 1 µm.

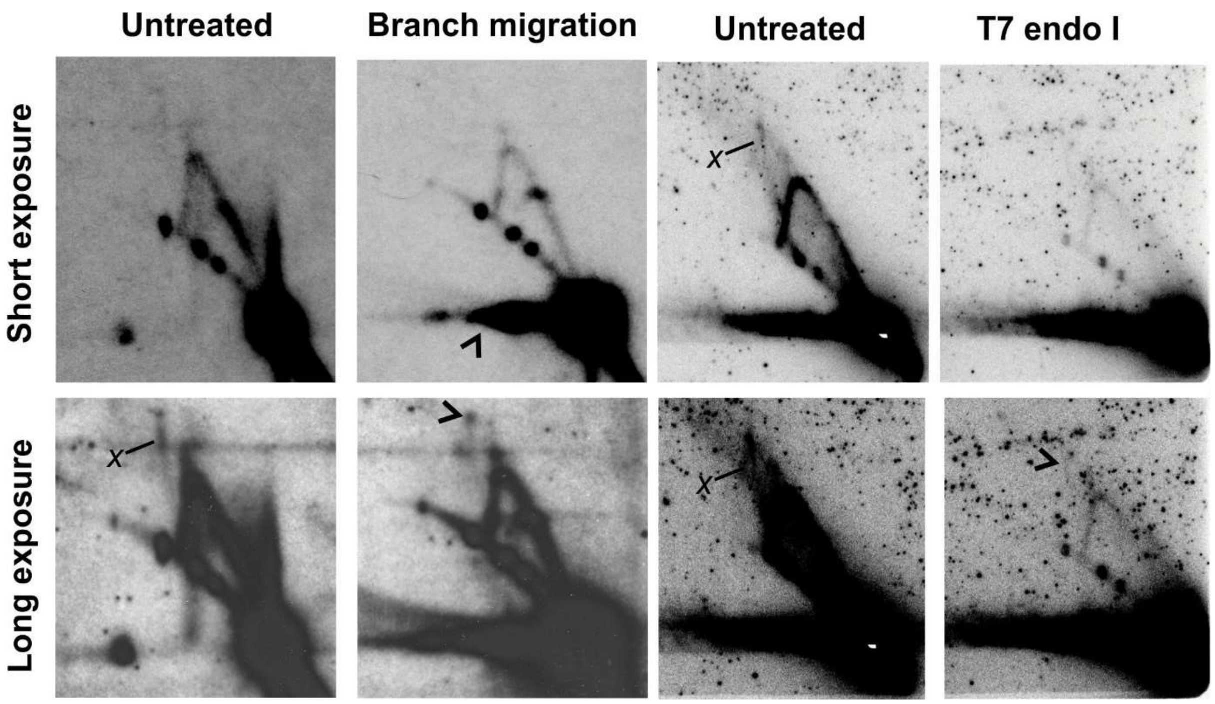

Supplementary Figure S9. Mitochondrial DNA *x*-forms are sensitive to branch migration and cruciform-cutting endonuclease. *Dra*I+S1 digest of regular, untreated HEK293T cell mtDNA, probed for the 12,273–15,012 fragment as in Figure 1. Untreated HEK293T cell mitochondria have low levels of x-forms (*x*), better visible in the long exposure of the blot (bottom panels). In-gel branch migration will resolve most of the replication intermediates, generating a streak of 1n sized fragments (arrowhead in the upper panel). Branch migration also resolves recombination junctions, leaving only the tip of the *x*-arc left. The tip of the *x*-arc consists of DNA fragments with the Holliday-junction close to the middle of the molecule and therefore most resistant to branch migration. The *x*-forms are also sensitive to cruciform-DNA cleaving T7 endonuclease I (right panels).

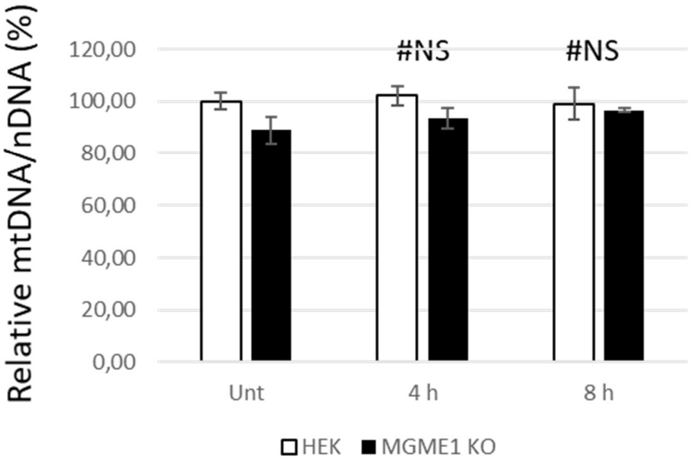

Supplementary Figure S10. UV treatment does not influence mtDNA copy number in MGME1 KO cells.

Supplementary Table

Supplementary Table S1. Reagents, enzymes, cell lines and key equipment used in the study.

**Reagent or Resource**	**Source**	**Identifier**
**Antibodies**		
Mouse anti-myc	Sigma-Aldrich	SAB4700447
Rabbit anti-flag	Antibodies online	ABIN871107
Goat-anti-mouse IgG HRP	Antibodies online	ABIN101744
Novex goat-anti-rabbit IgG HRP	LifeTechnologies	A16104
**Essential chemicals and enzymes**		
2´,3´-dideoxycytidine	Sigma-Aldrich	7481-89-2
Ethidium bromide	Sigma-Aldrich	E7637
Rediprime™ II	Sigma-Aldrich	GERPN1634
[α-32P]-dCTP, 3,000 Ci/mmol	PerkinElmer	NEG013H100UC
FastDigest *Afl*II	Thermo Scientific	FD0834
FastDigest *Bam*HI	Thermo Scientific	FD0055
FastDigest *Bgl*II	Thermo Scientific	FD0084
FastDigest *Dra*I	Thermo Scientific	FD0224
FastDigest *Hinc*II	Thermo Scientific	FD0494
FastDigest *Hind*III	Thermo Scientific	FD0504
FastDigest *Pvu*II	Thermo Scientific	FD0634
T7 endonuclease I	New England Biolabs	M0302
S1 nuclease	Thermo Scientific	18001016
**Cell lines**		
HEK293T	ECACC	85120602
Flp-InTM T-RExTM 293 parental cell line	Thermo-Fischer Scientific	R71007
Flp-InTM T-RExTM 293 TWNK wild type	Wanrooij et al., 2007	TWNK OE
Flp-InTM T-RExTM 293 TWNK linker duplication	Goffart et al., 2009	TWNK-LD
Flp-InTM T-RExTM 293 MGME1 wild type	Szczesny et al., 2013	MGME1 OE
MGME1 KO	This paper	*MGME1* KO
**Oligonucleotides**		
Probe primers (see methods for sequences)	Thermo-Fischer Scientific	NA
RT-PCR oligos (see methods for sequences)	Thermo-Fischer Scientific	NA
**Recombinant DNA**		
pcDNA5/FRT/TO-TWNK-WT	Wanrooij et al., 2007	TWNK-WT-myc
pcDNA5/FRT/TO-TWNK-LD	Goffart et al., 2009	TWNK-LD-myc
pcDNA5/FRT/TO-MGME1-WT	Szczesny et al., 2013	MGME1-WT-flag
**Other**		
BioSpectrum ® 810 imaging System	UVP	BioSpectrum 810
Molecular Imager ® FX	Bio-Rad	Molecular Imager FX
FEI Tecnai T12 TEM with a Gatan 2kx2k Orius CCD	FEI	NA
AriaMx Real-Time PCR system	Agilent Technologies	G8830A
BAS-IP MS screen	GE Healthcare	GE28-9564-76
Carestream Kodak MS	Sigma-Aldrich	Z363006

Torregrosa *et al.*

Original scans of blots

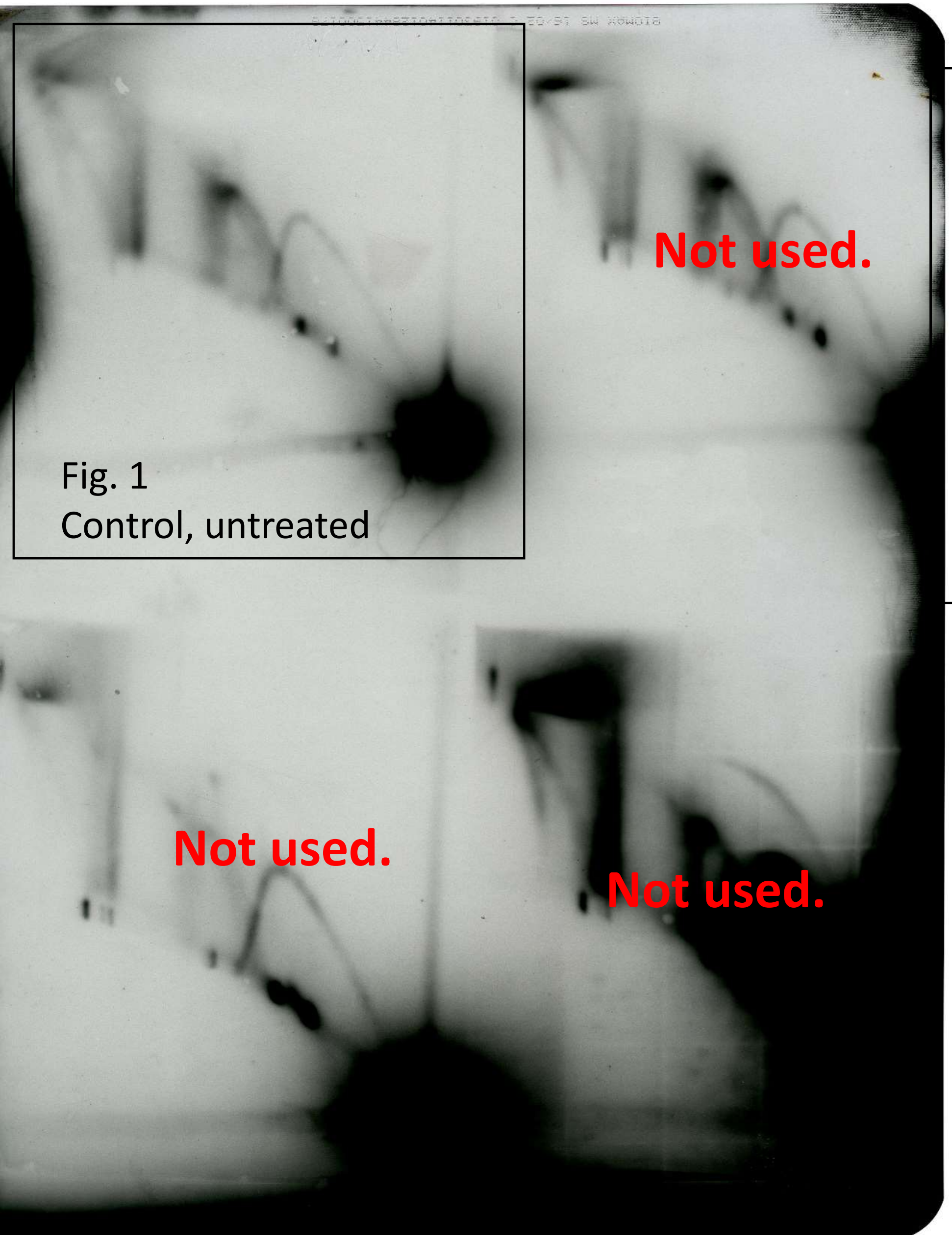
Fig. 1
Control, untreated
Not used.
Not used.
Not used.

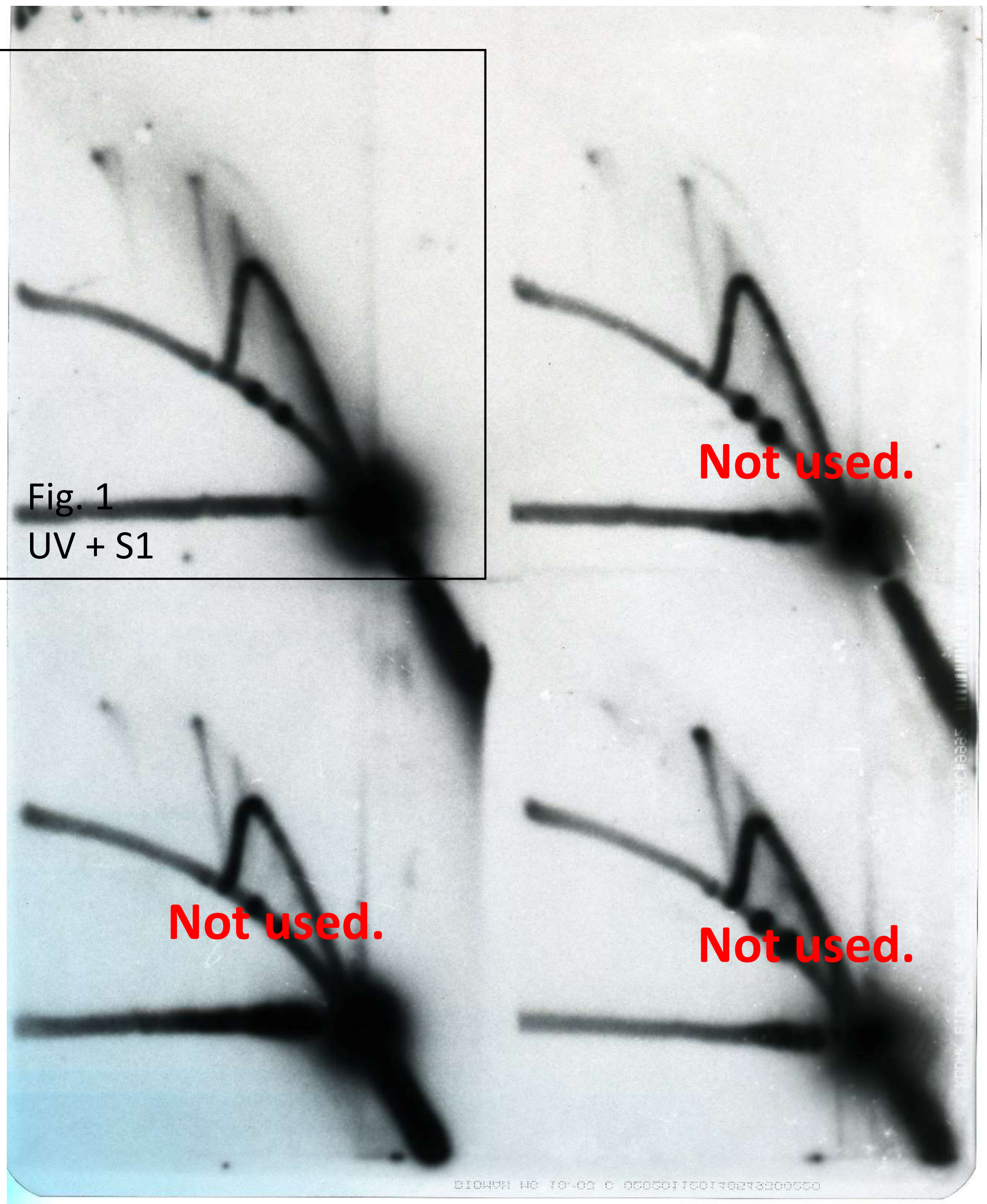
Fig. 1
UV + S1
Not used.
Not used.
Not used.

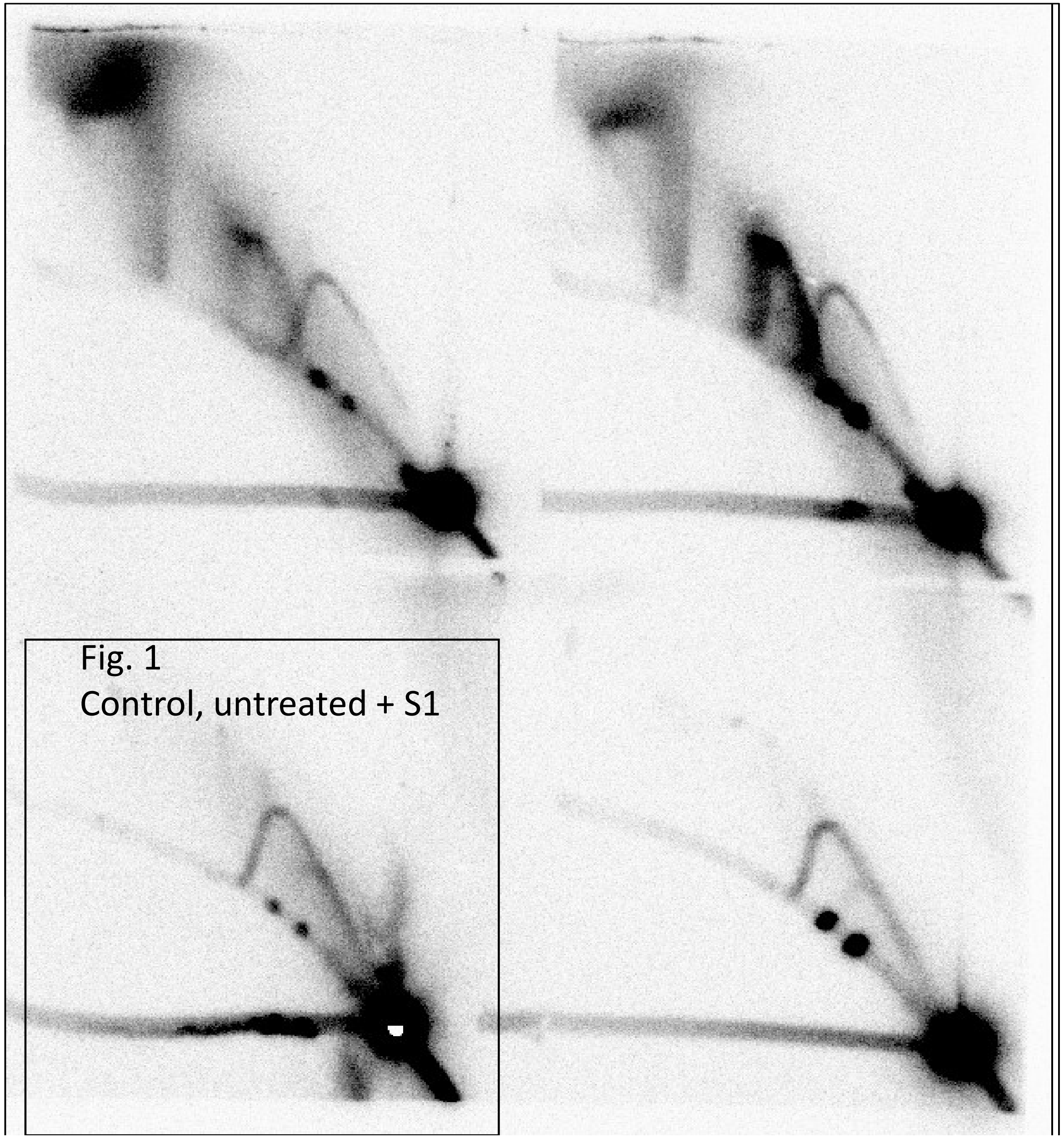

Fig. 1
Control, untreated + S1

Fig. 1D, short and long exposures

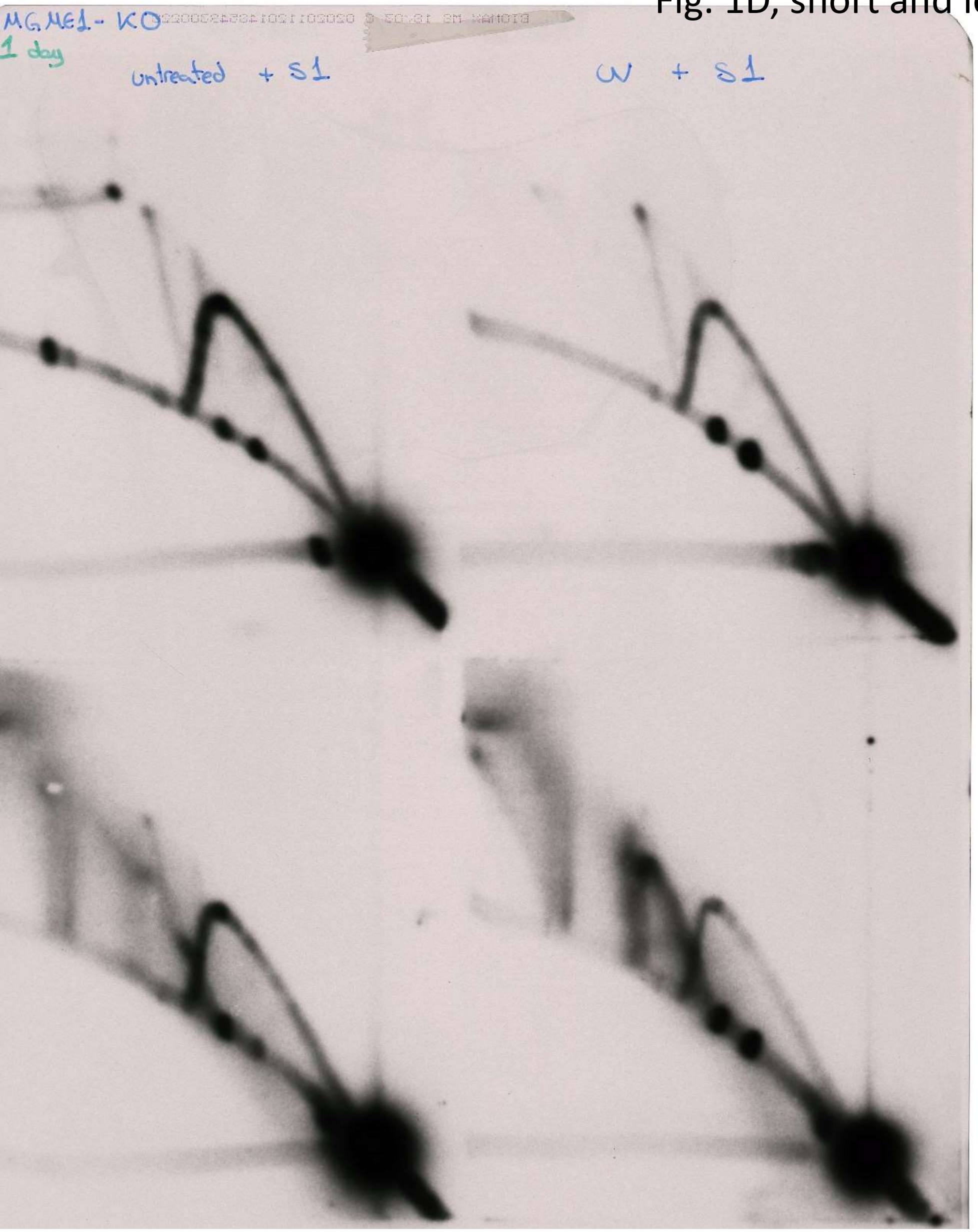

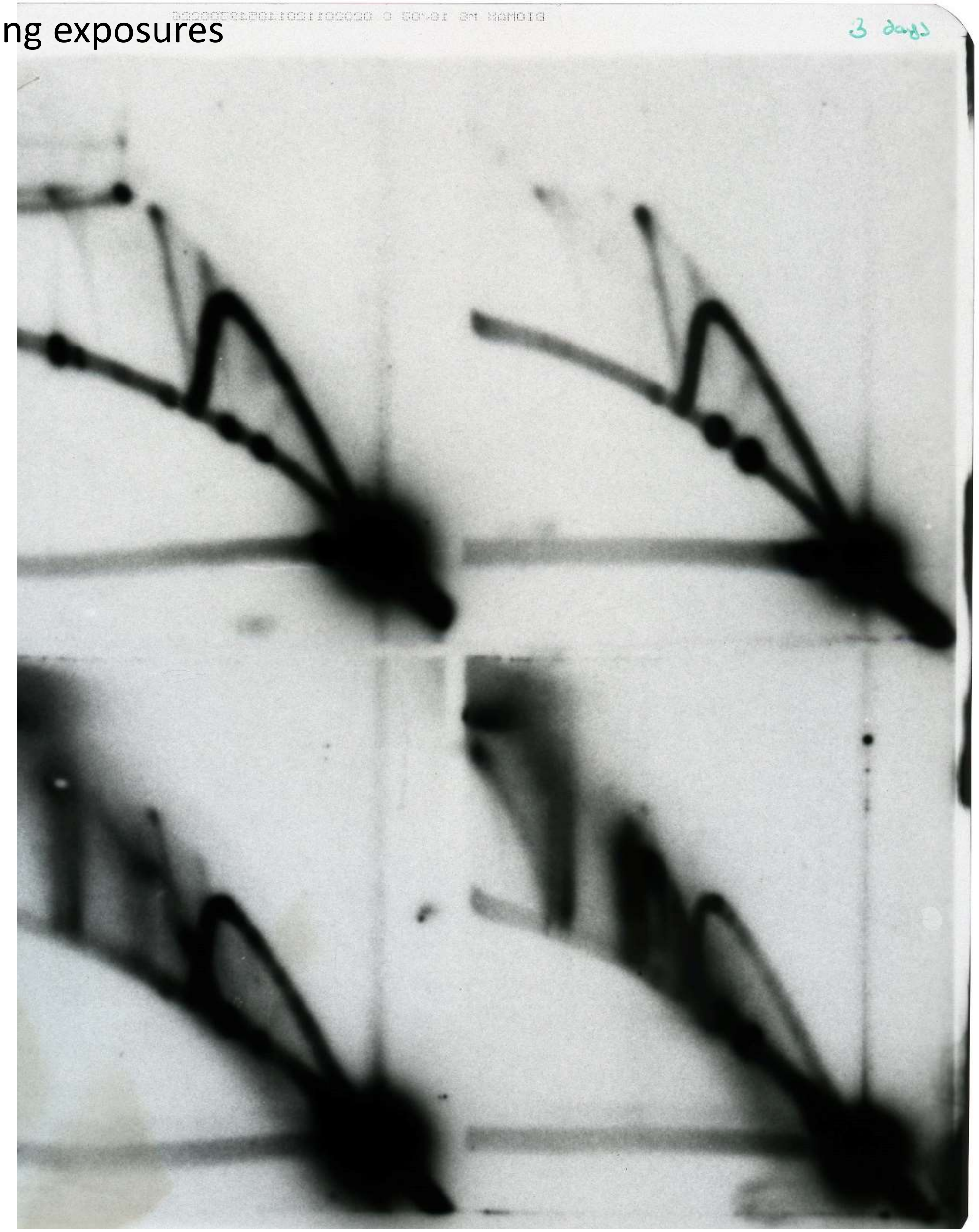

Two blots from 25.05.18

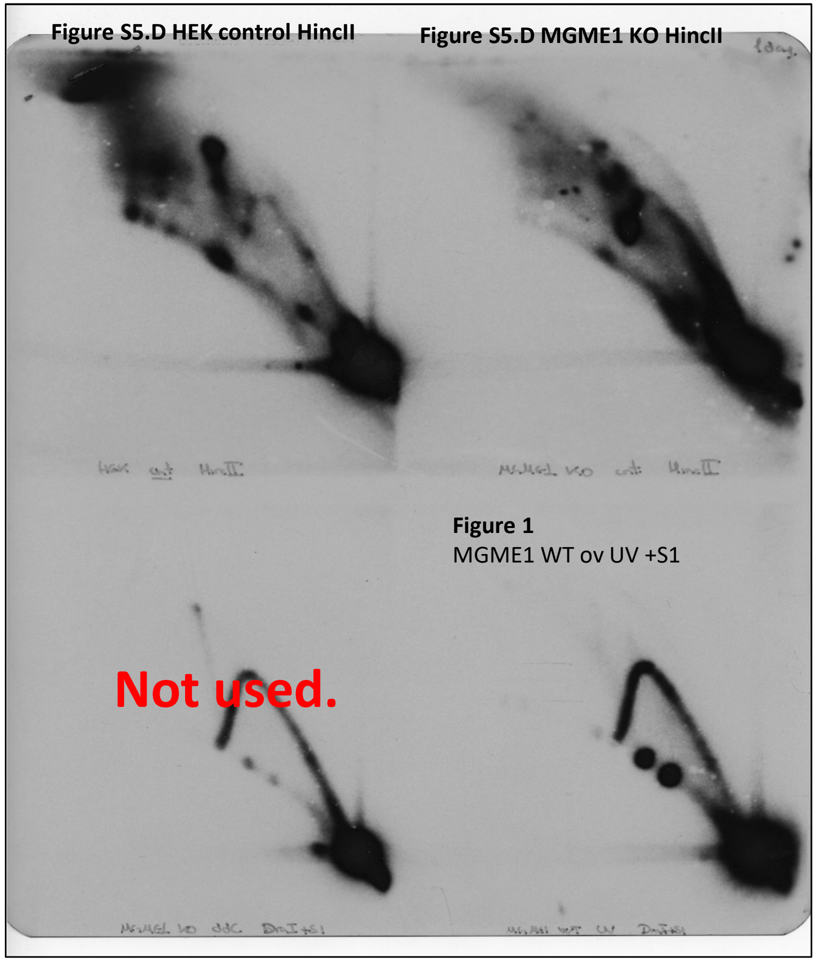

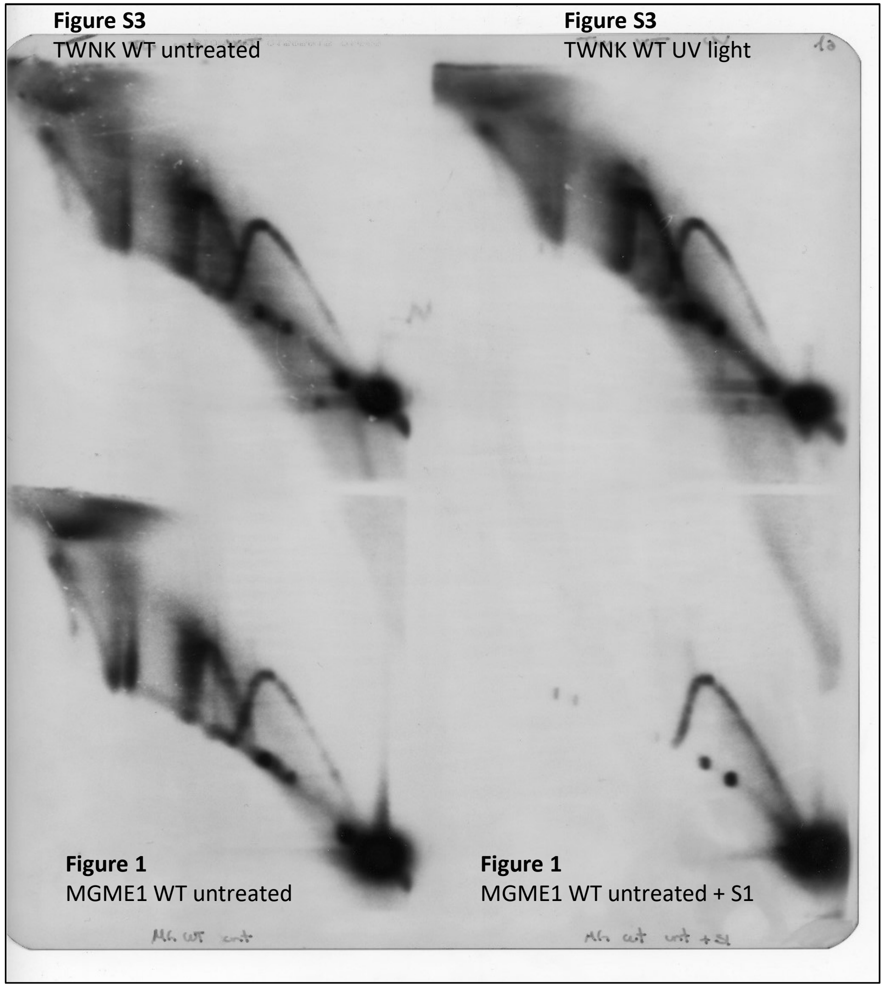

Figure 2B and C,

Long and short exposures

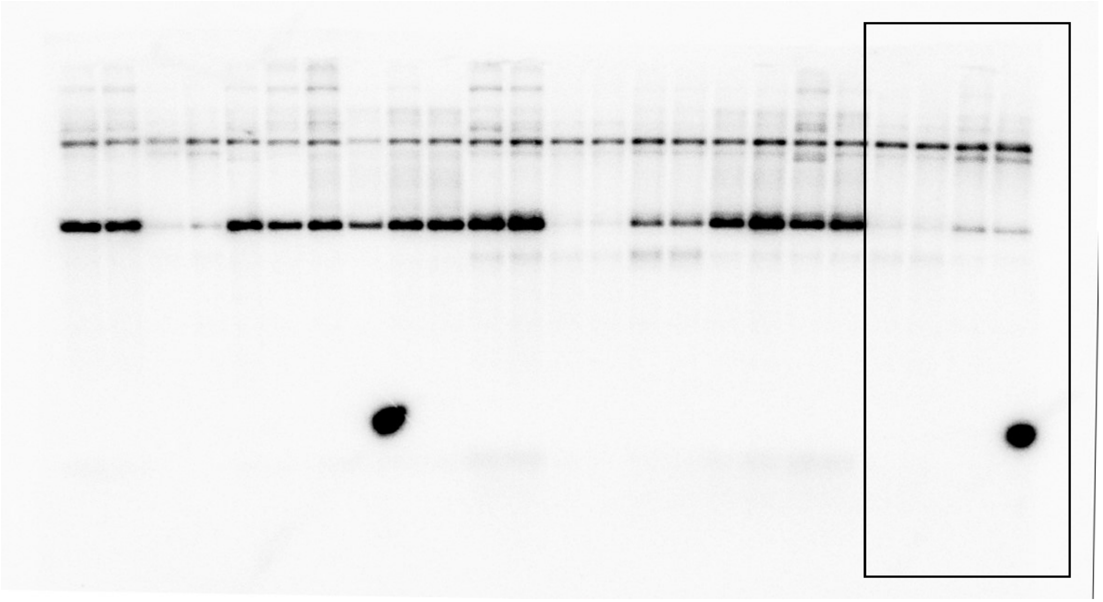

Cropped and flipped in Fig 2C

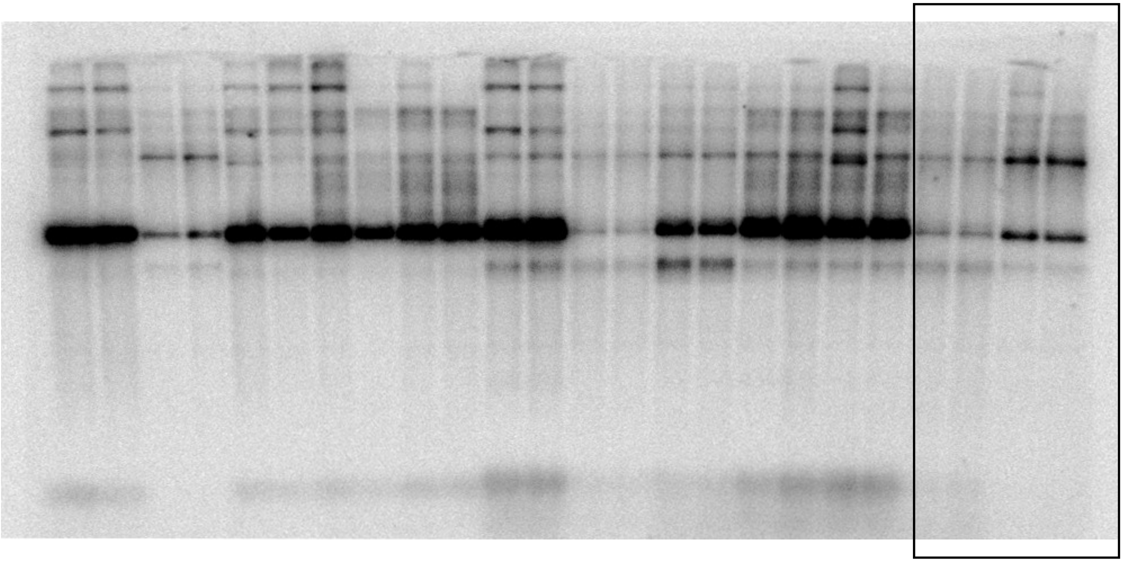

Cropped and flipped in Fig 2C

Fig. 3A-B

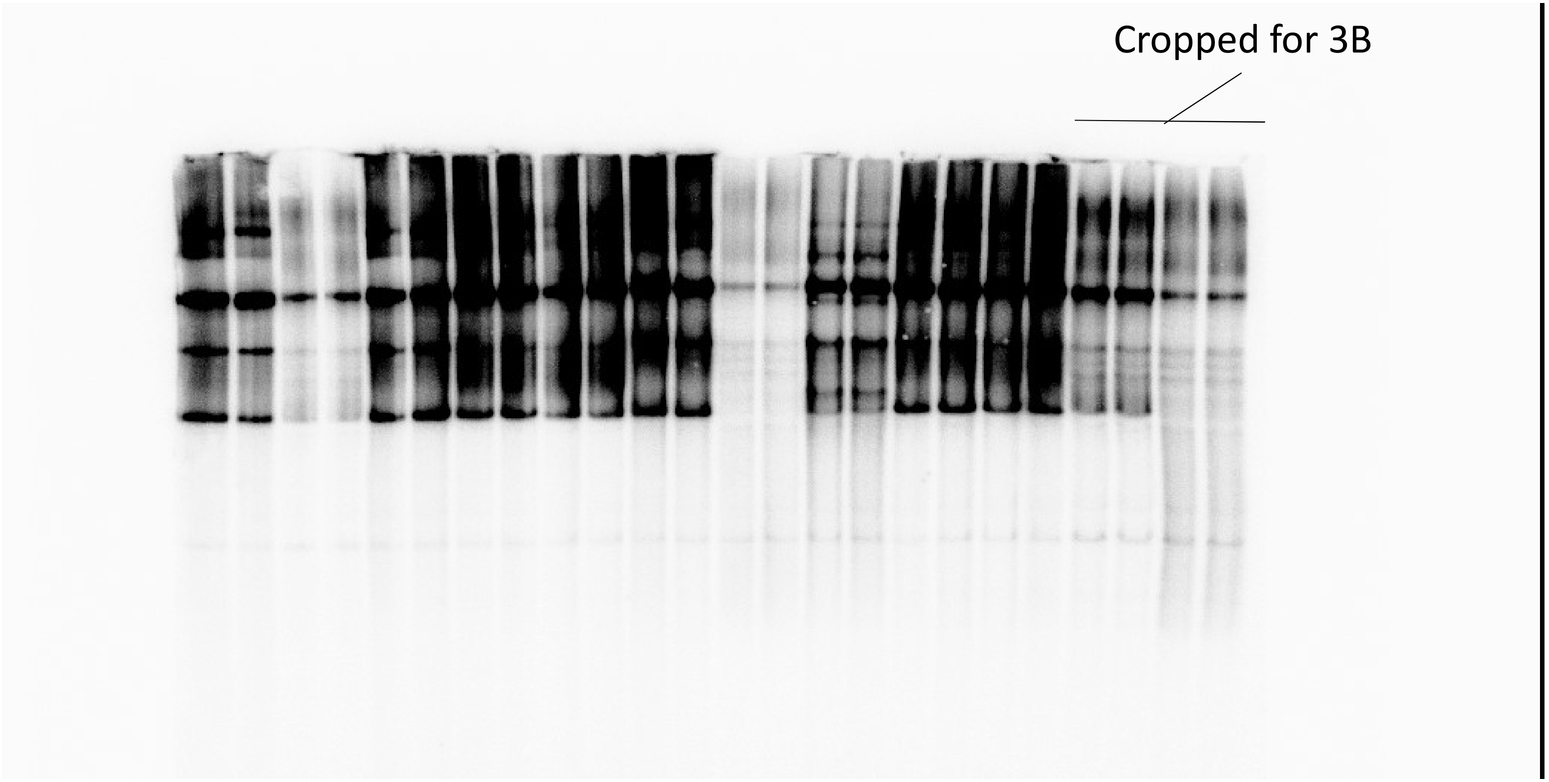

Fig. 3C, F

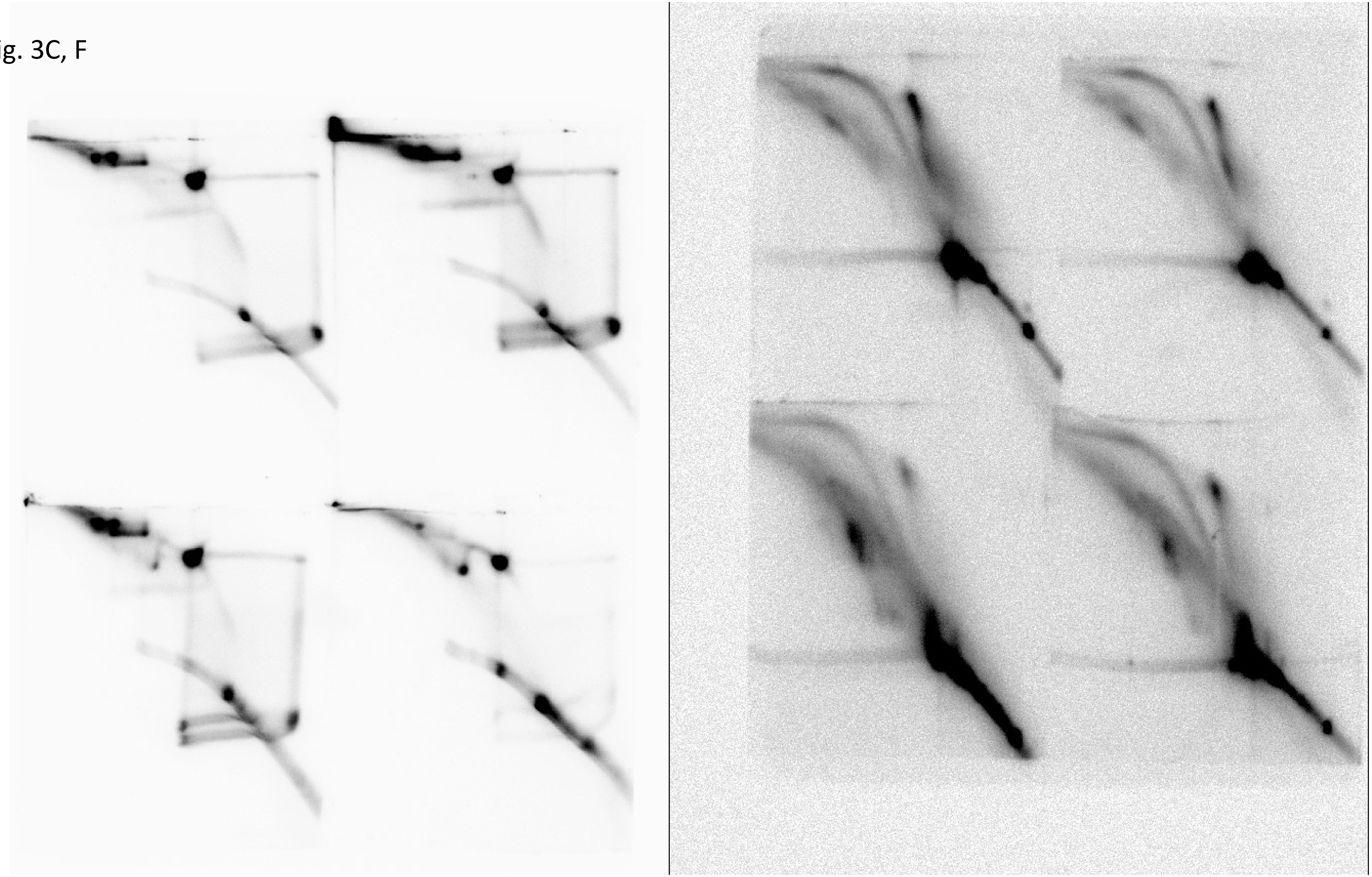

Idem longer exposure

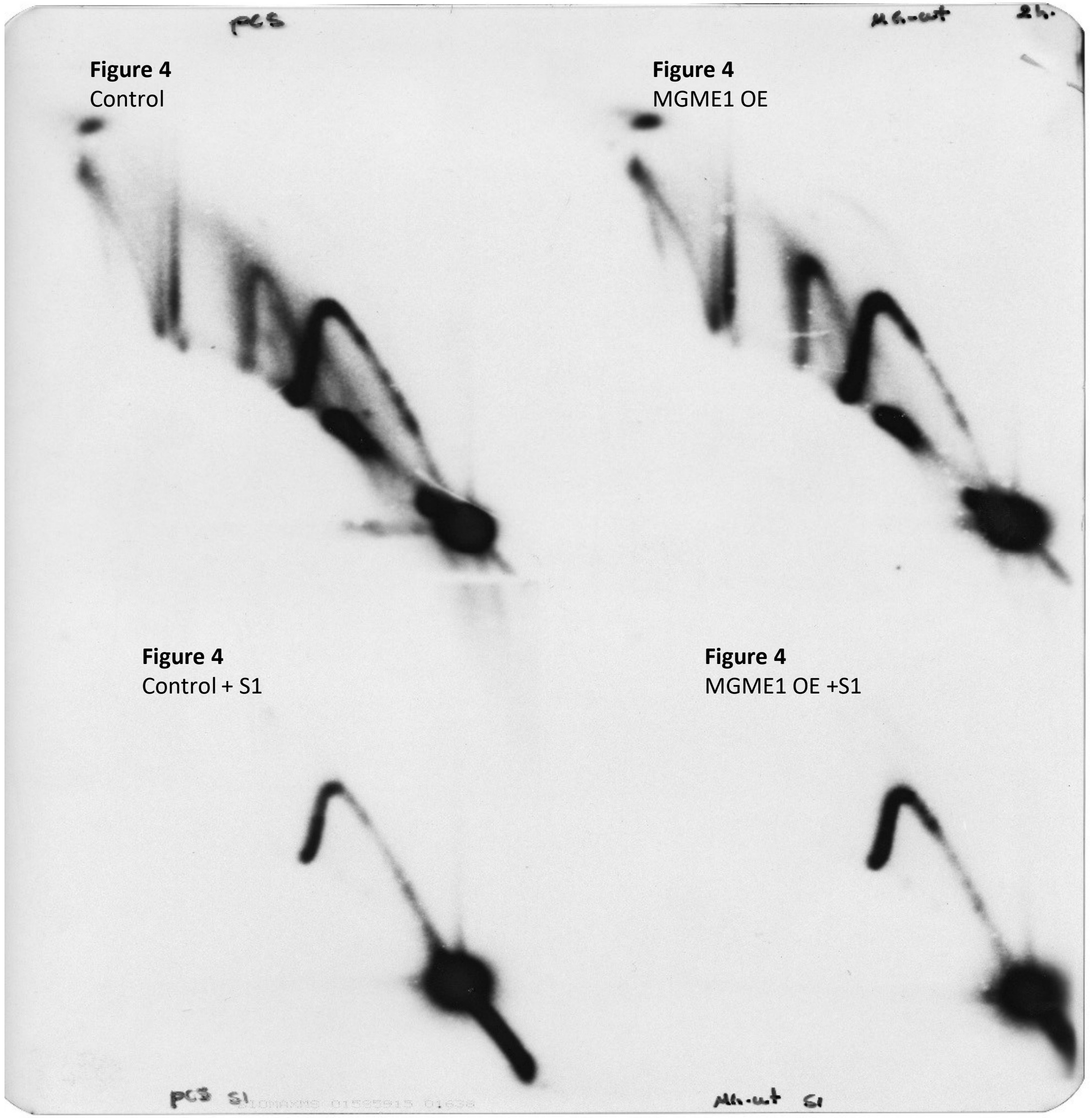

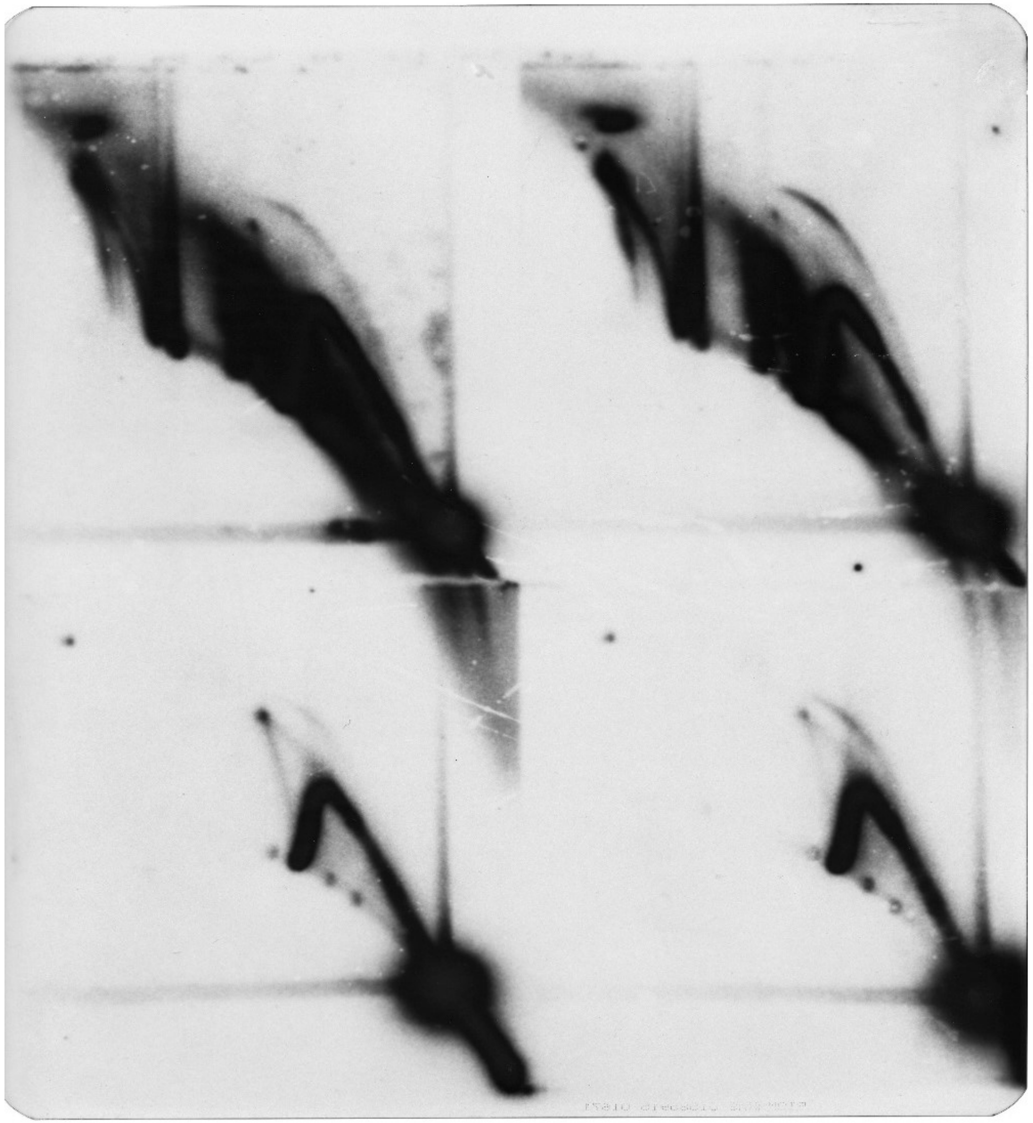

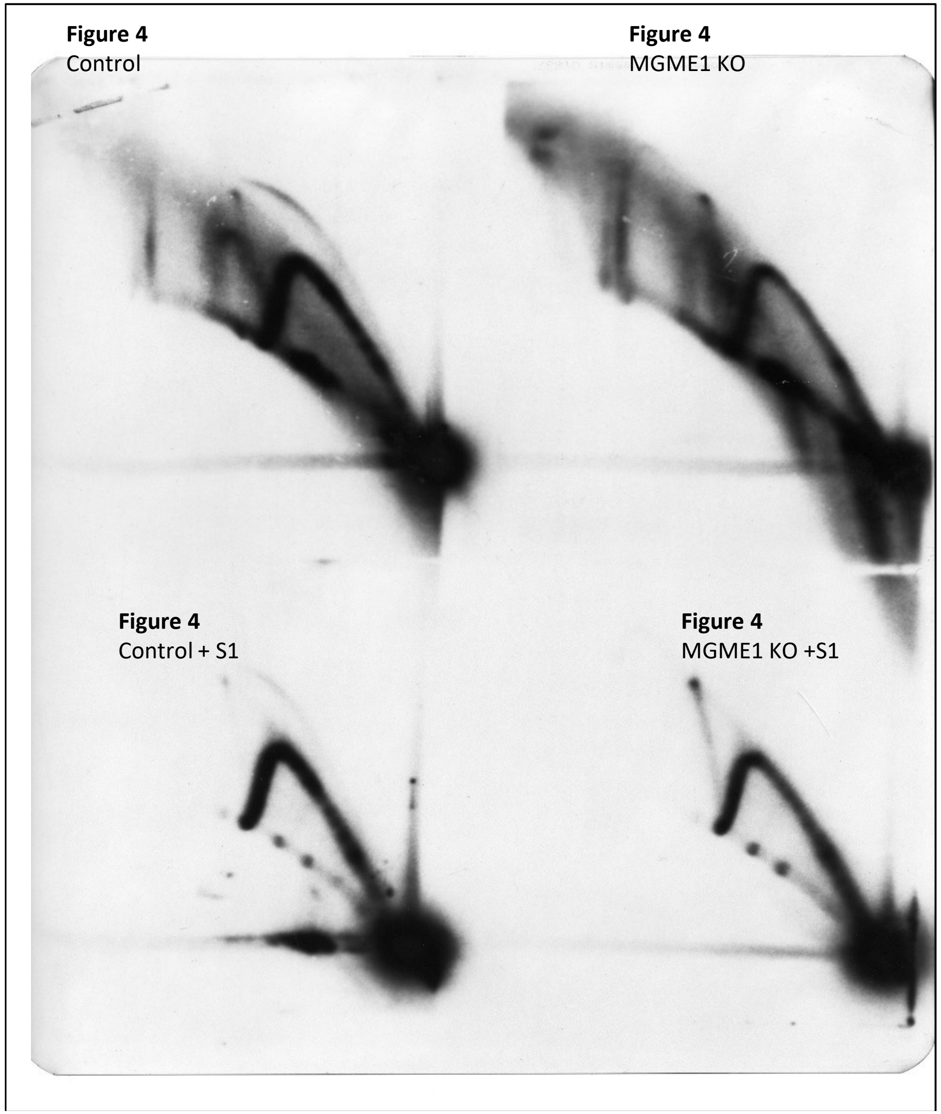

Idem longer exposure

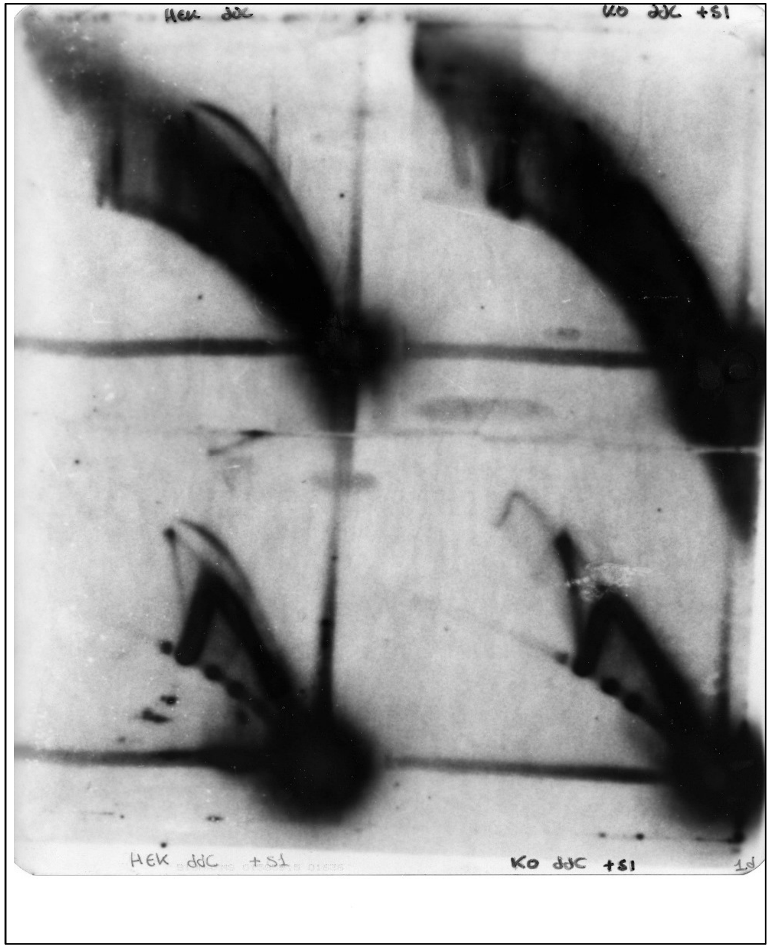

Fig. 7

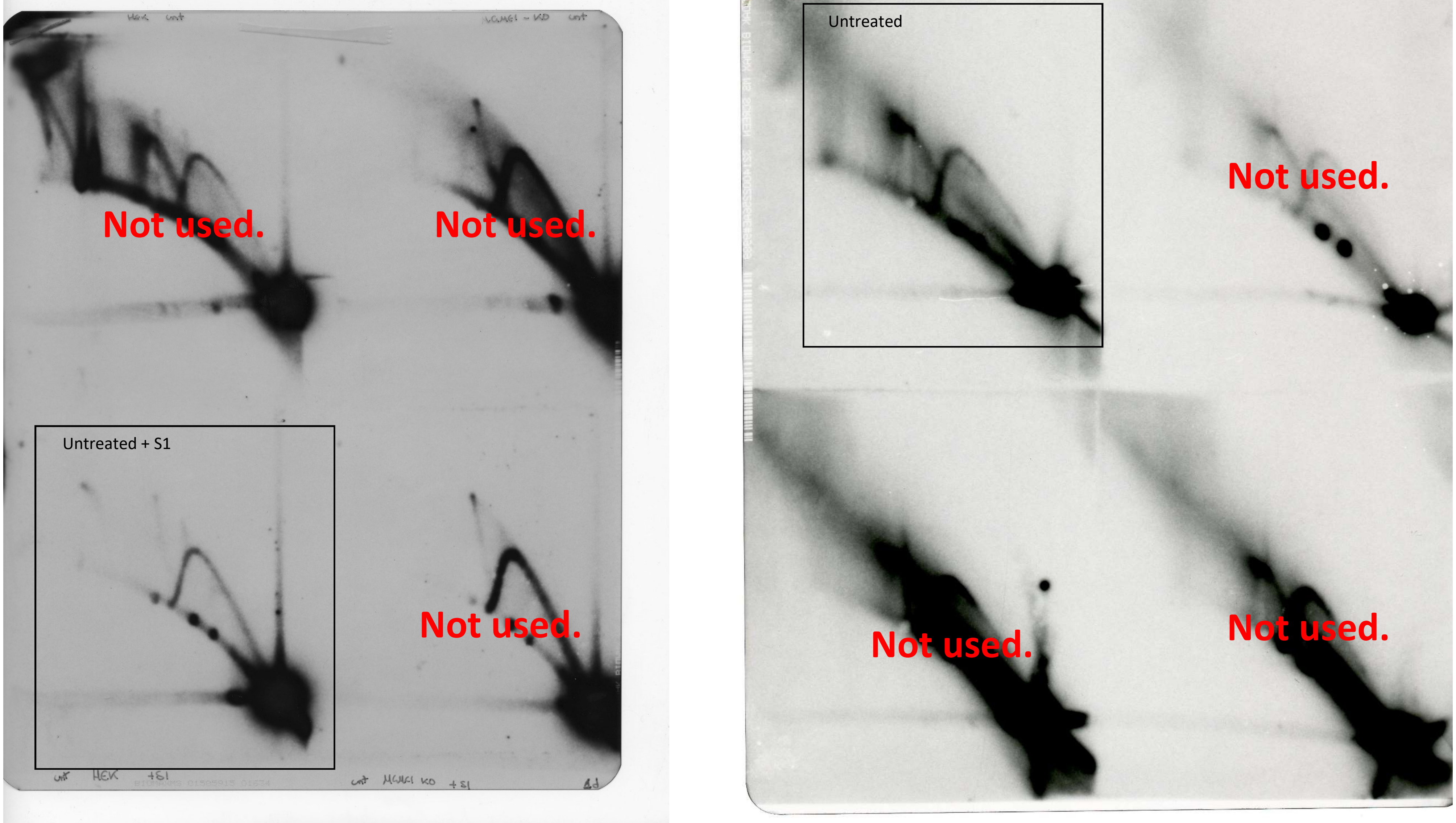
Not used.
Not used.
Untreated + S1
Not used.
Untreated
Not used.
Not used.
Not used.

Fig. 7

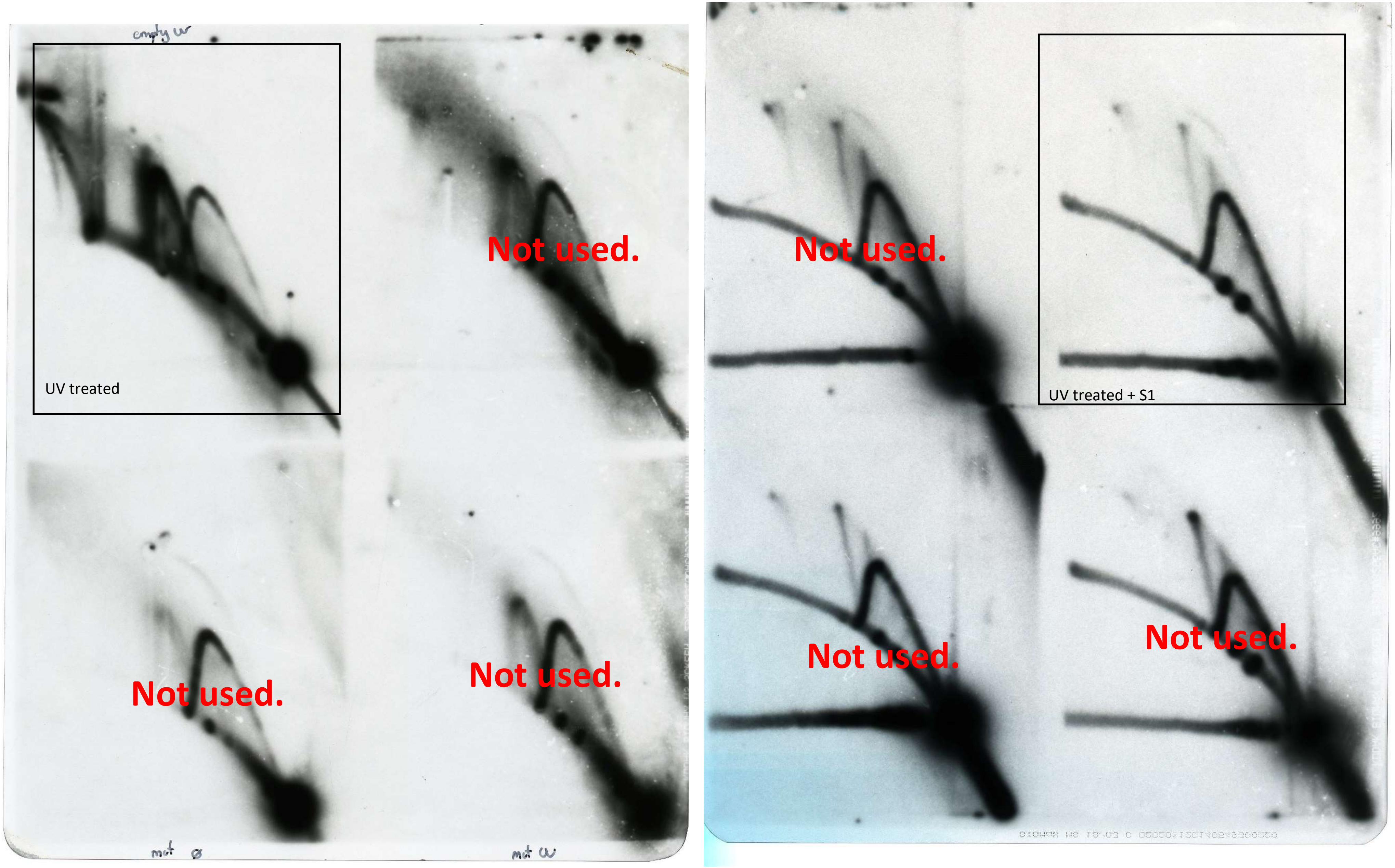

empty uv
UV treated
Not used.
Not used.
Not used.
Not used.
Not used.
Not used.
UV treated + S1
mut ø
mut uv

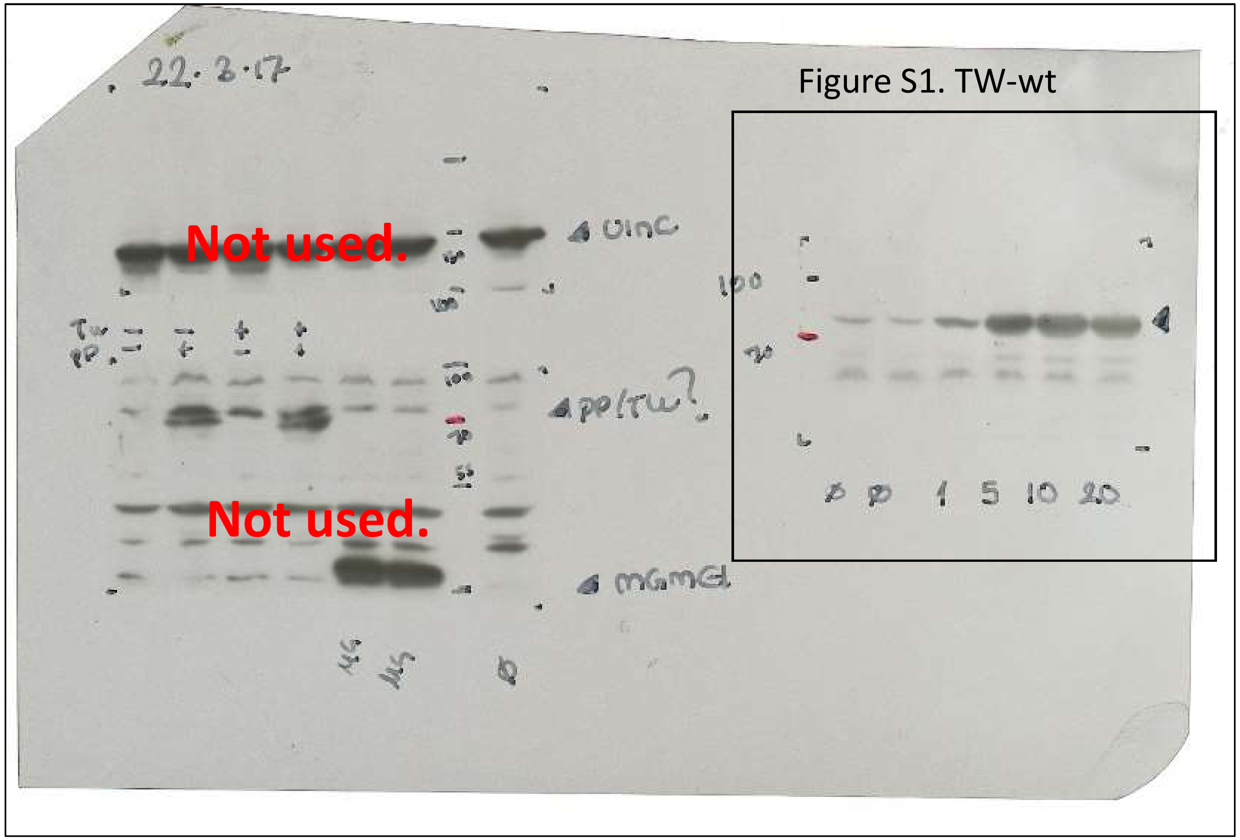
Figure S1. TW-wt
Not used.
Not used.

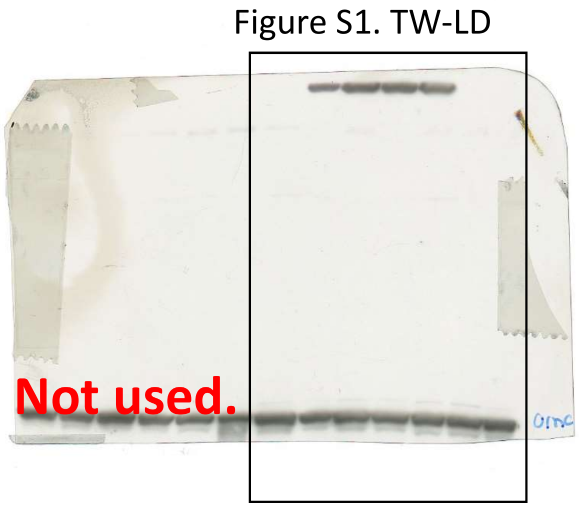
Figure S1. TW-LD
Not used.

Figure S1

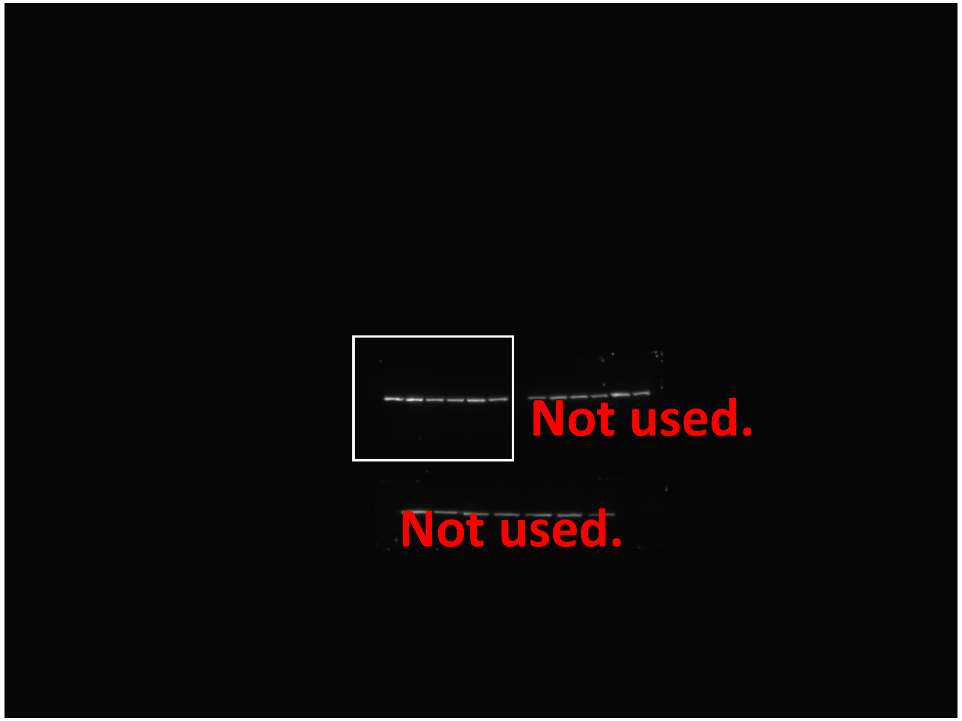

Vinculin

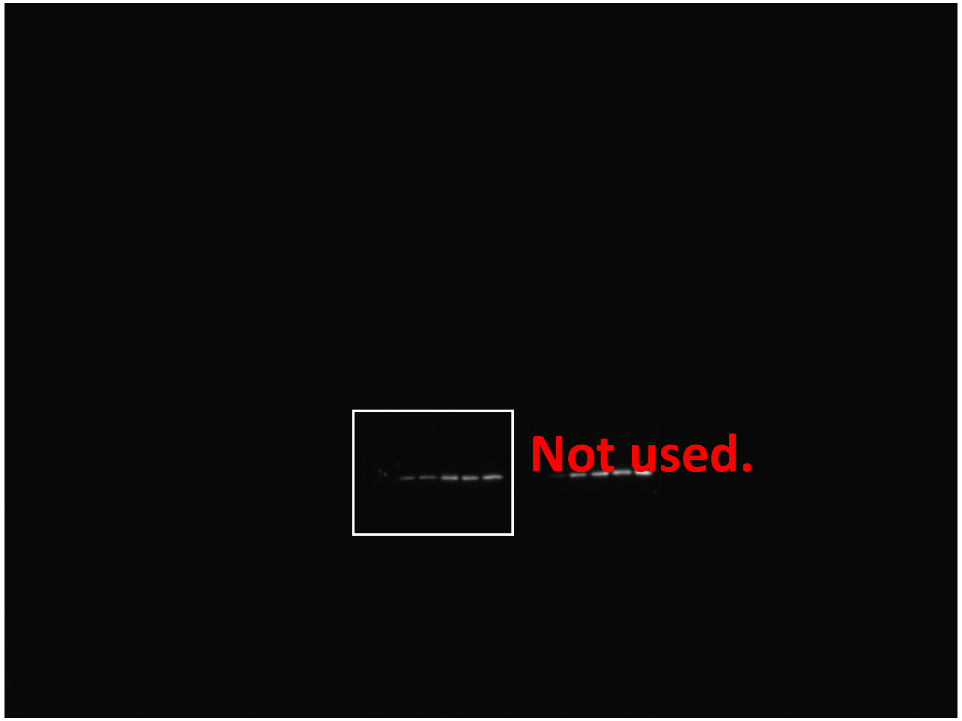

MGME1-Flag

Figure S2

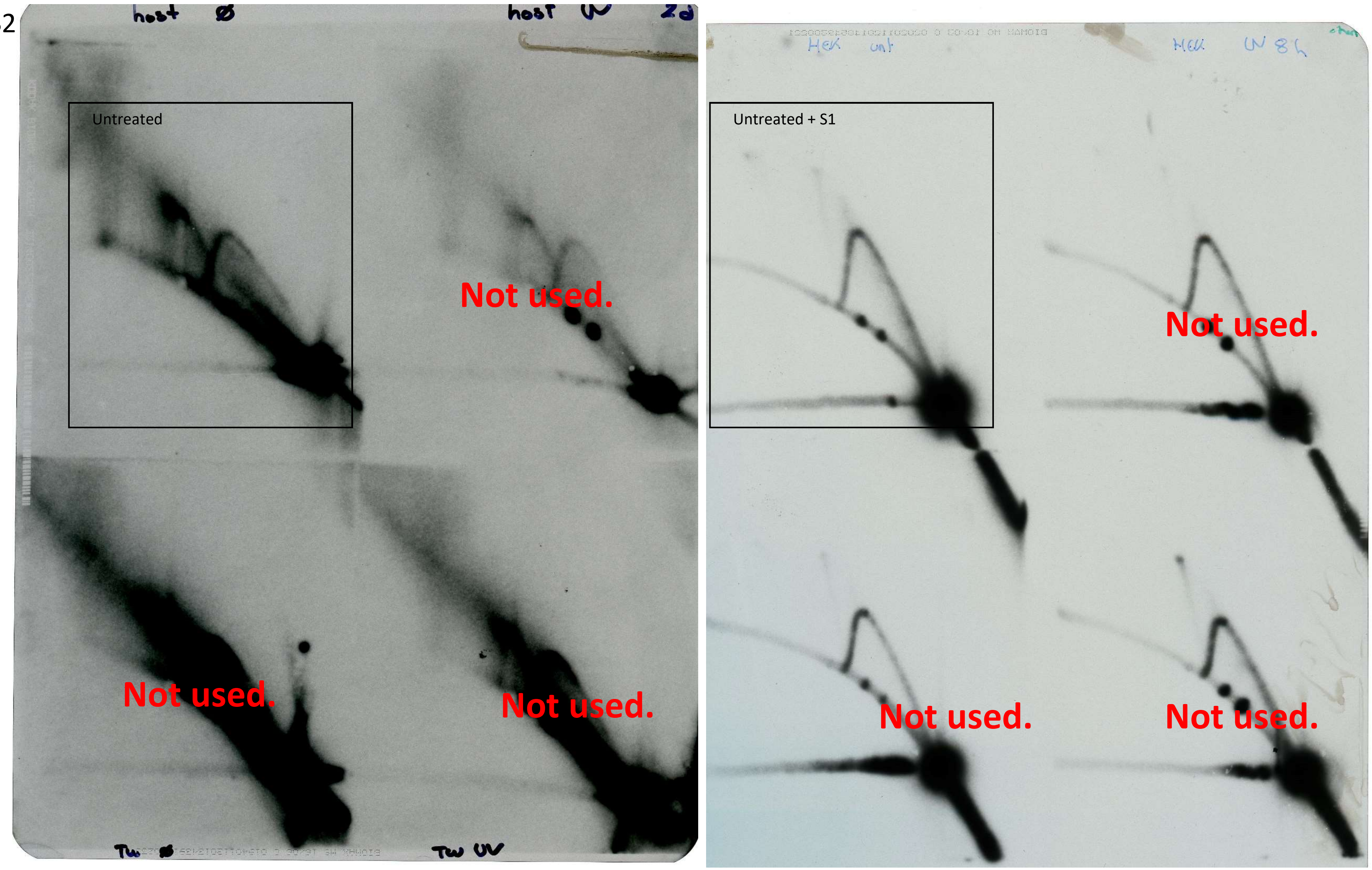
host
host
Untreated
Not used.
Not used.
Not used.
Untreated + S1
Not used.
Not used.
Not used.

Figure S2

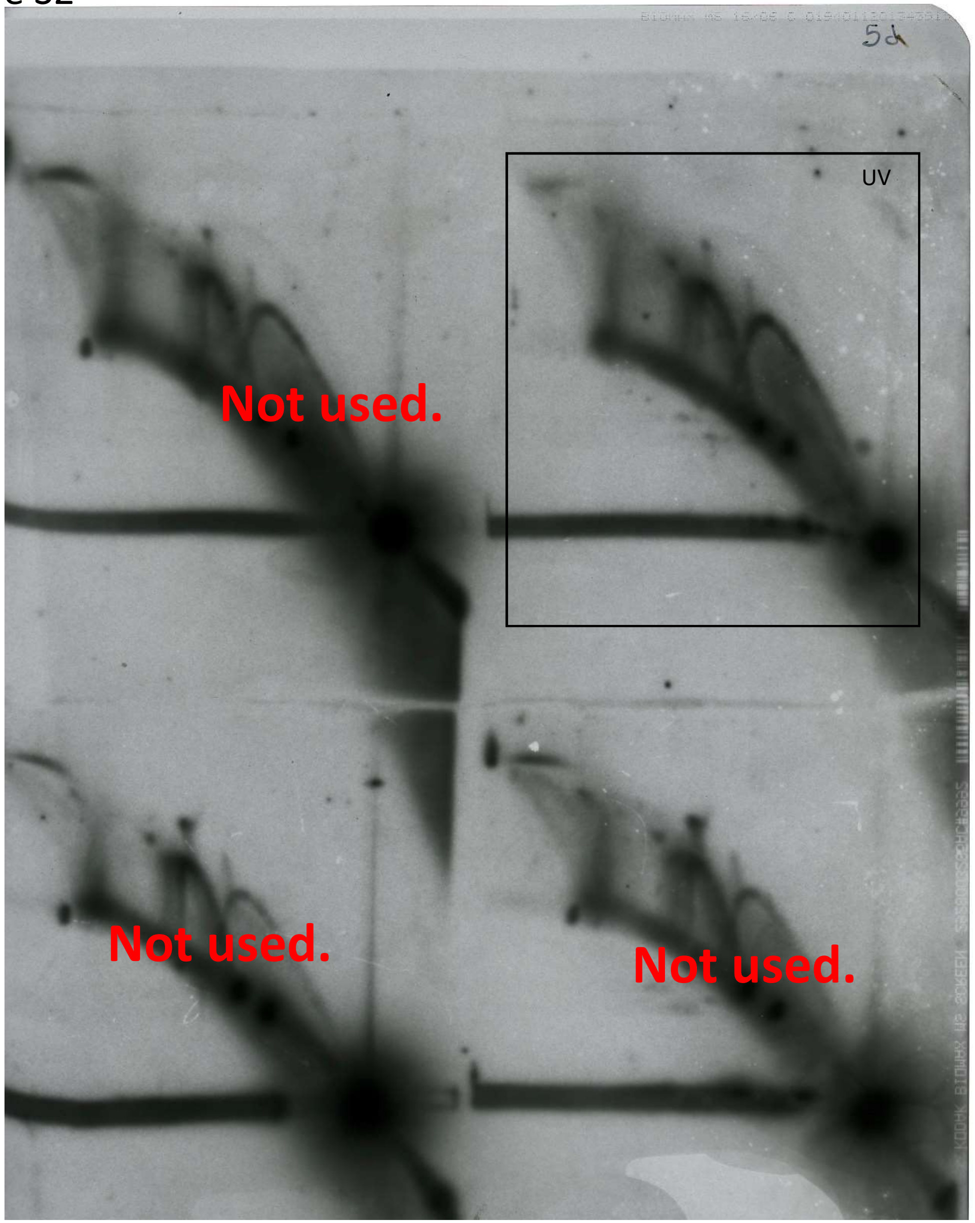
5d
UV
Not used.
Not used.
Not used.

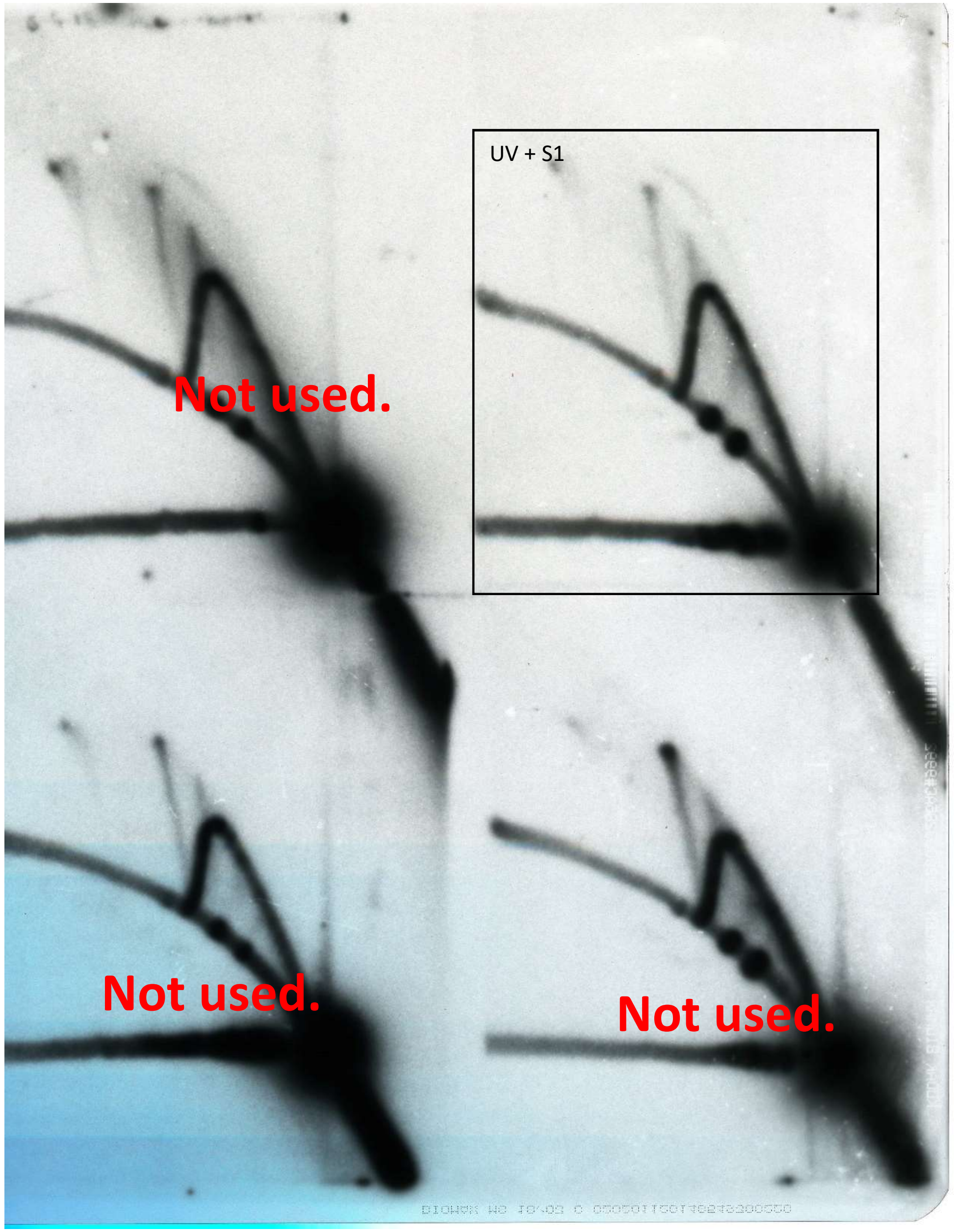
UV + S1
Not used.
Not used.
Not used.

Figure S3

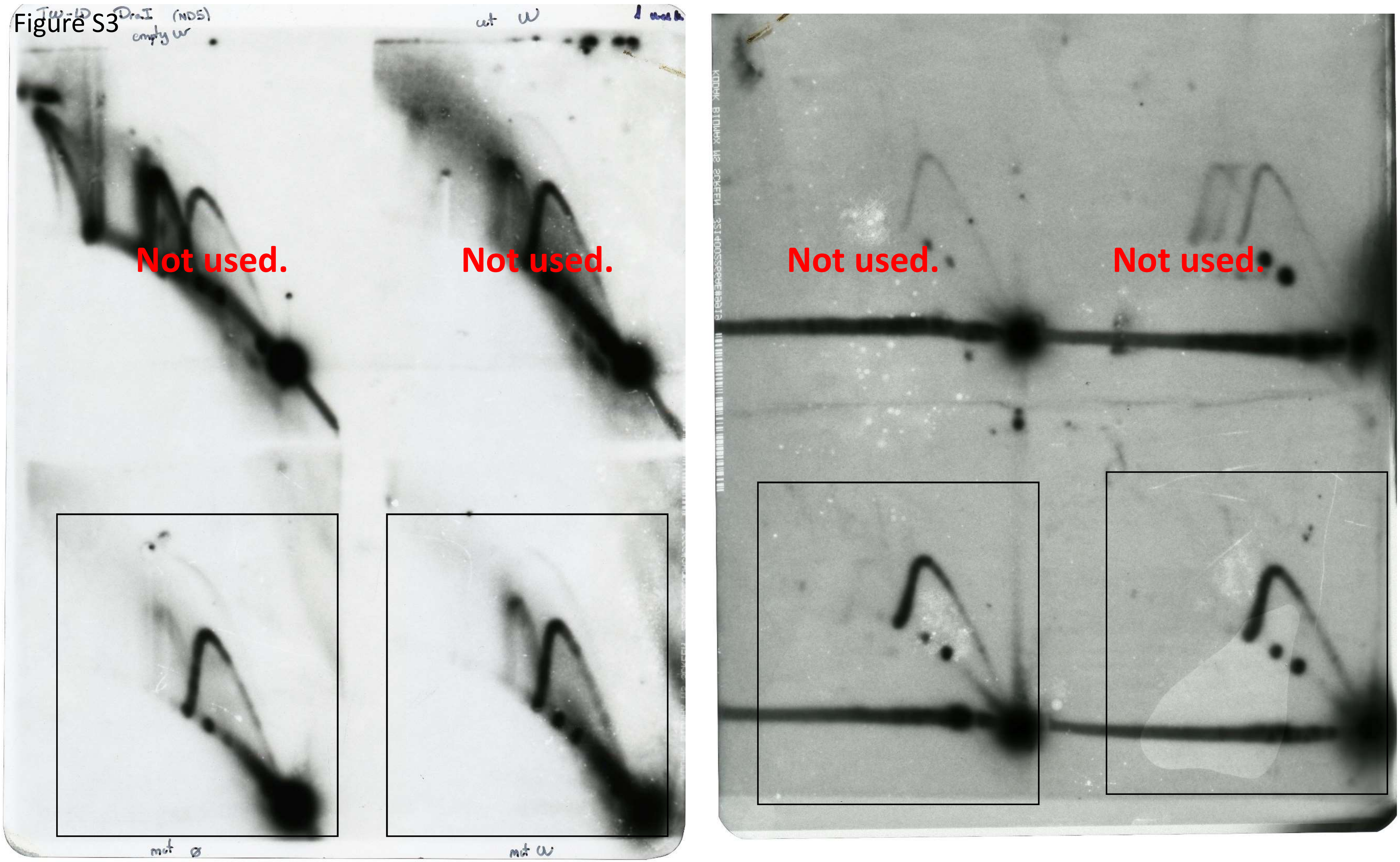

Figure S3

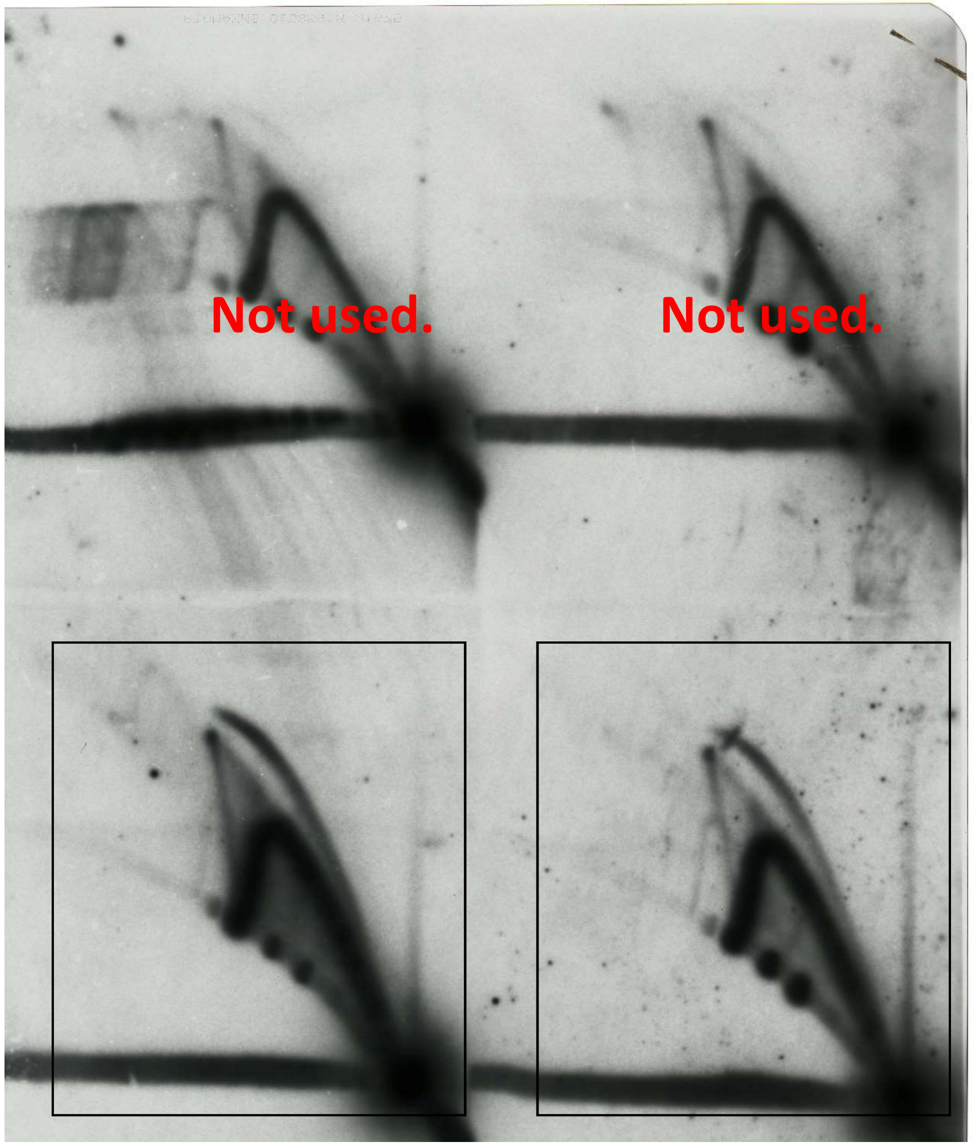

Figure S4

Short exposure

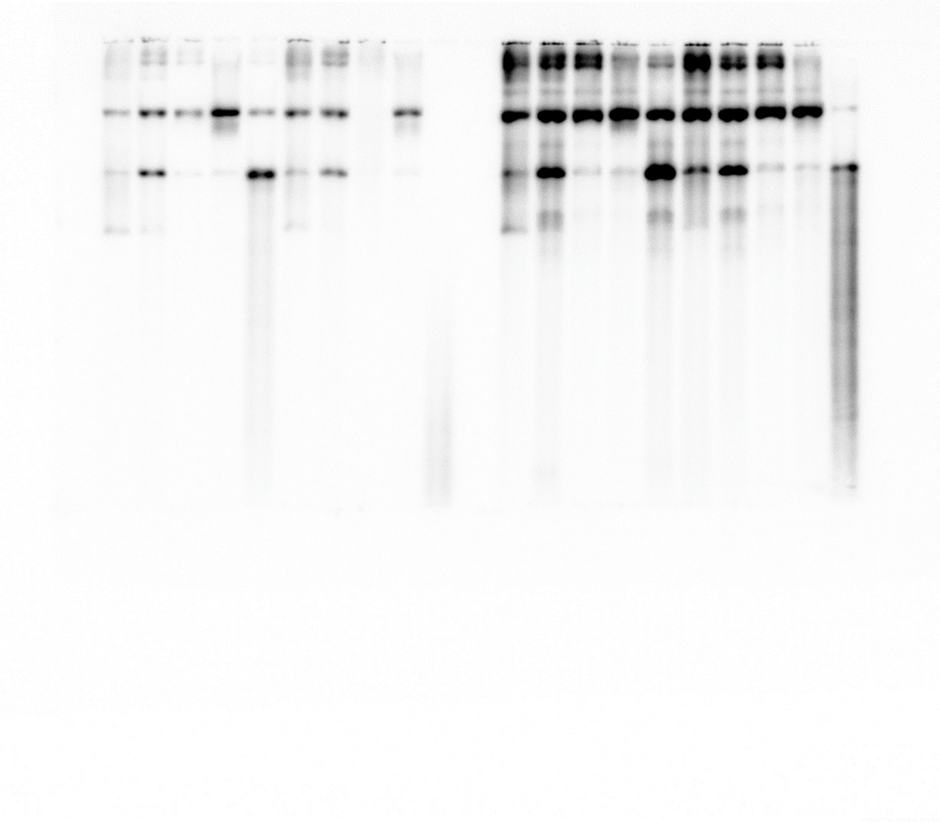

Long exposure

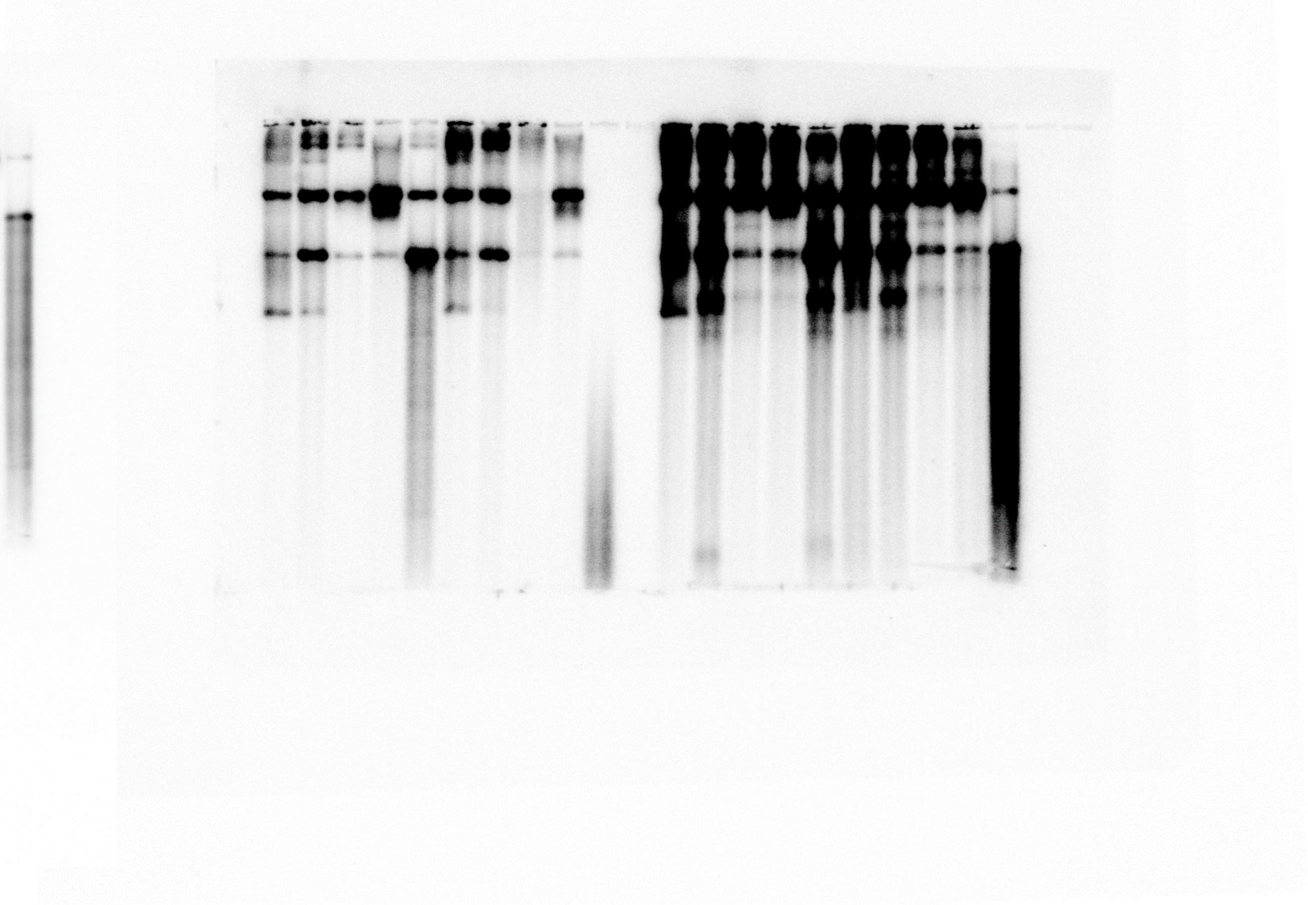

Figure S5

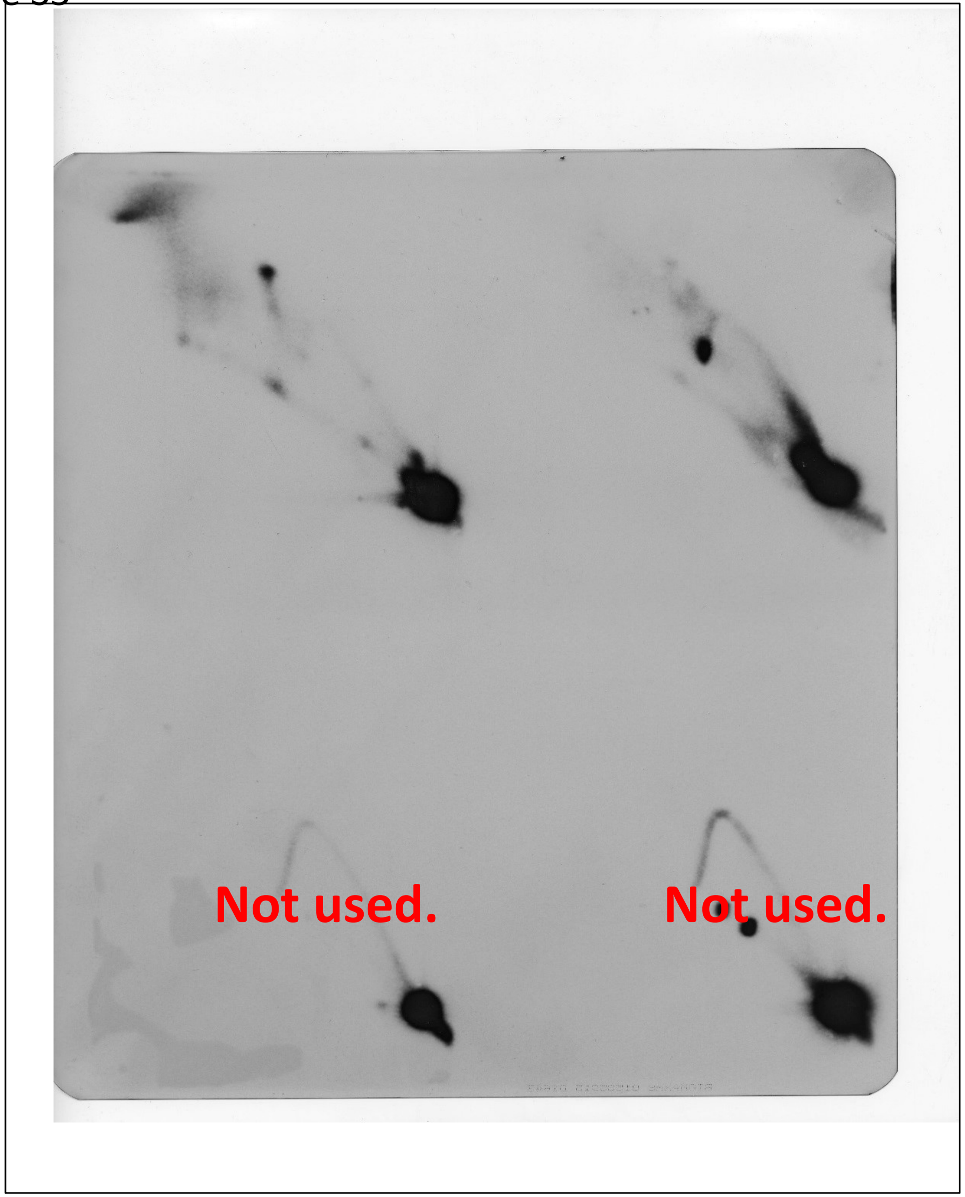

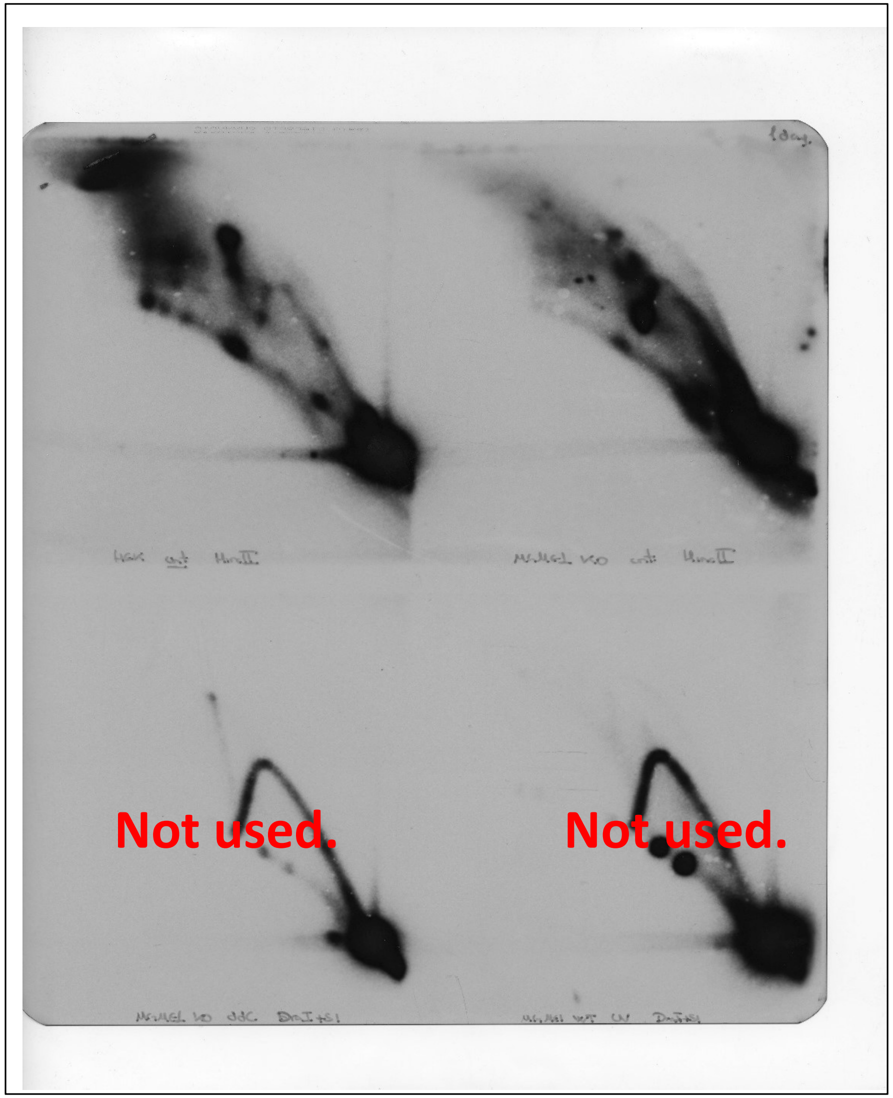

Figure S5

Phosphoimager scan

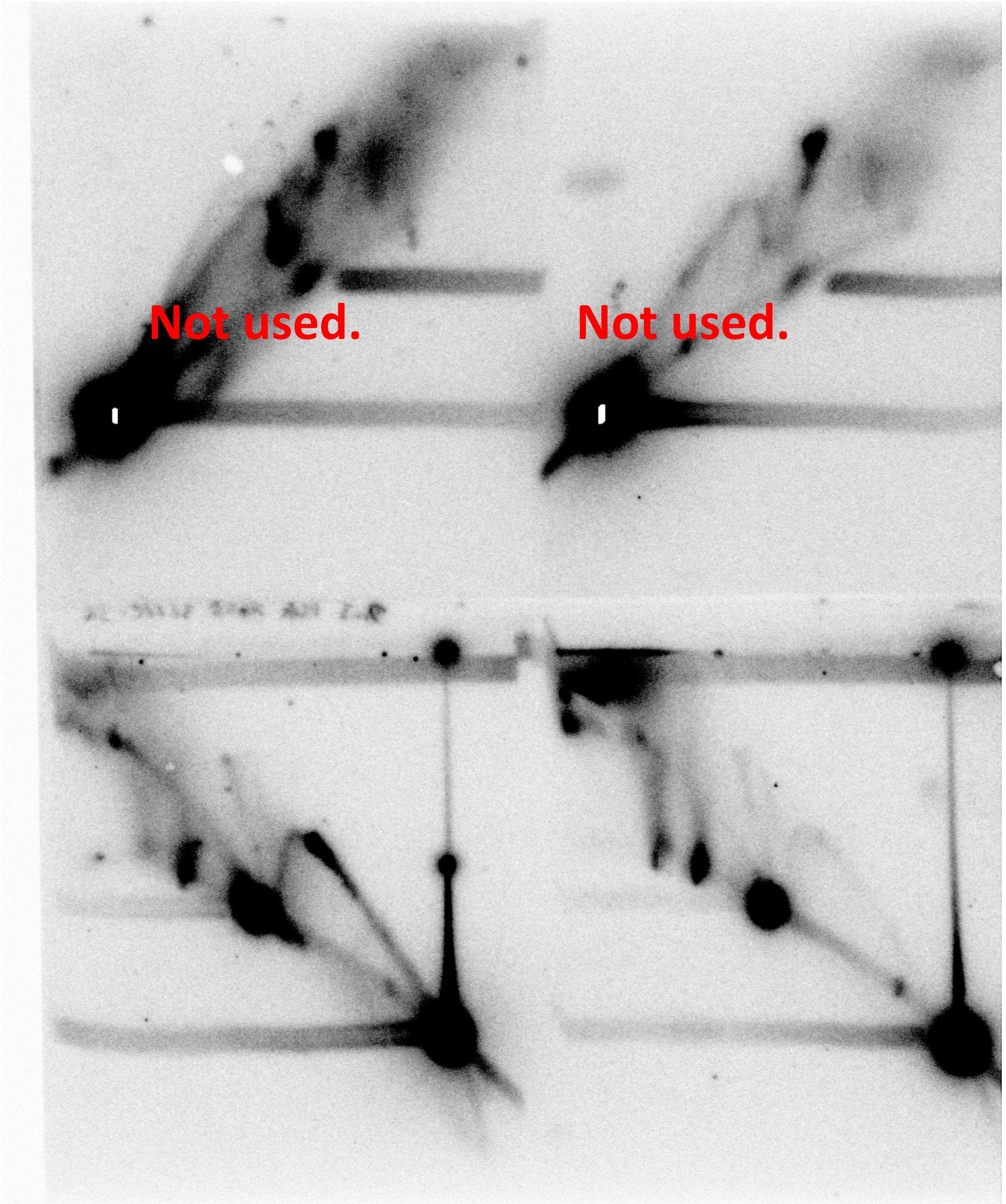

Figure S6

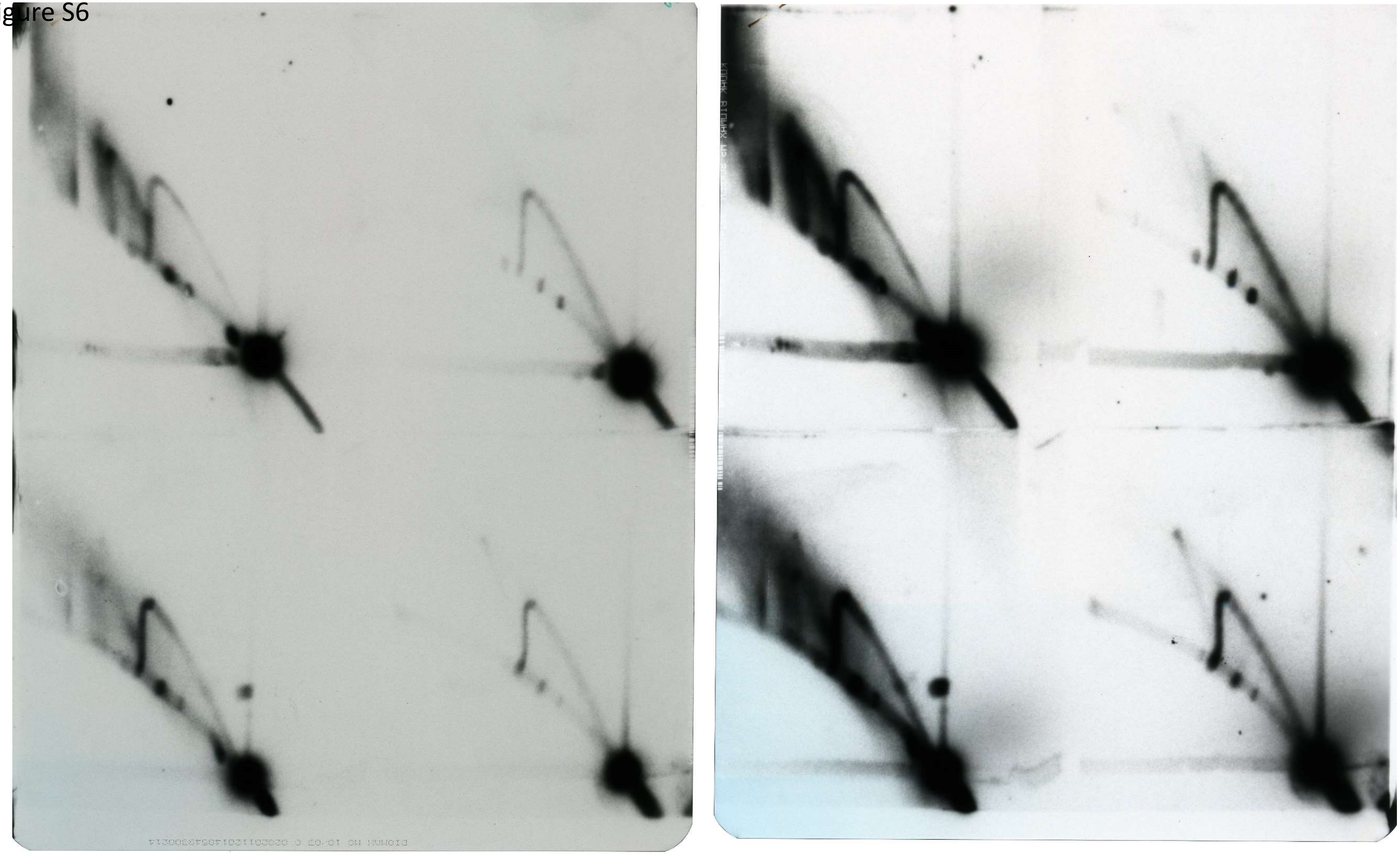

Figure S9

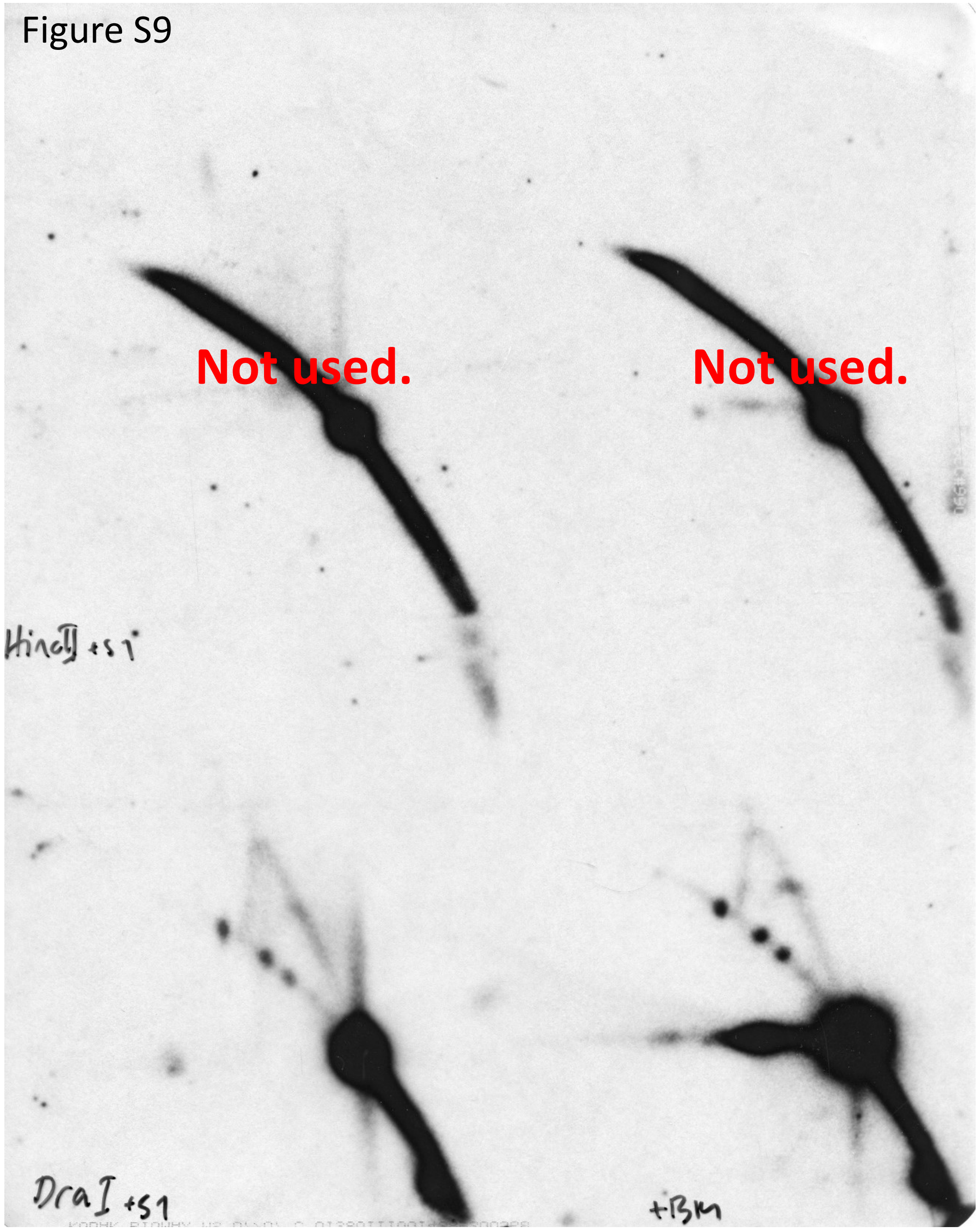

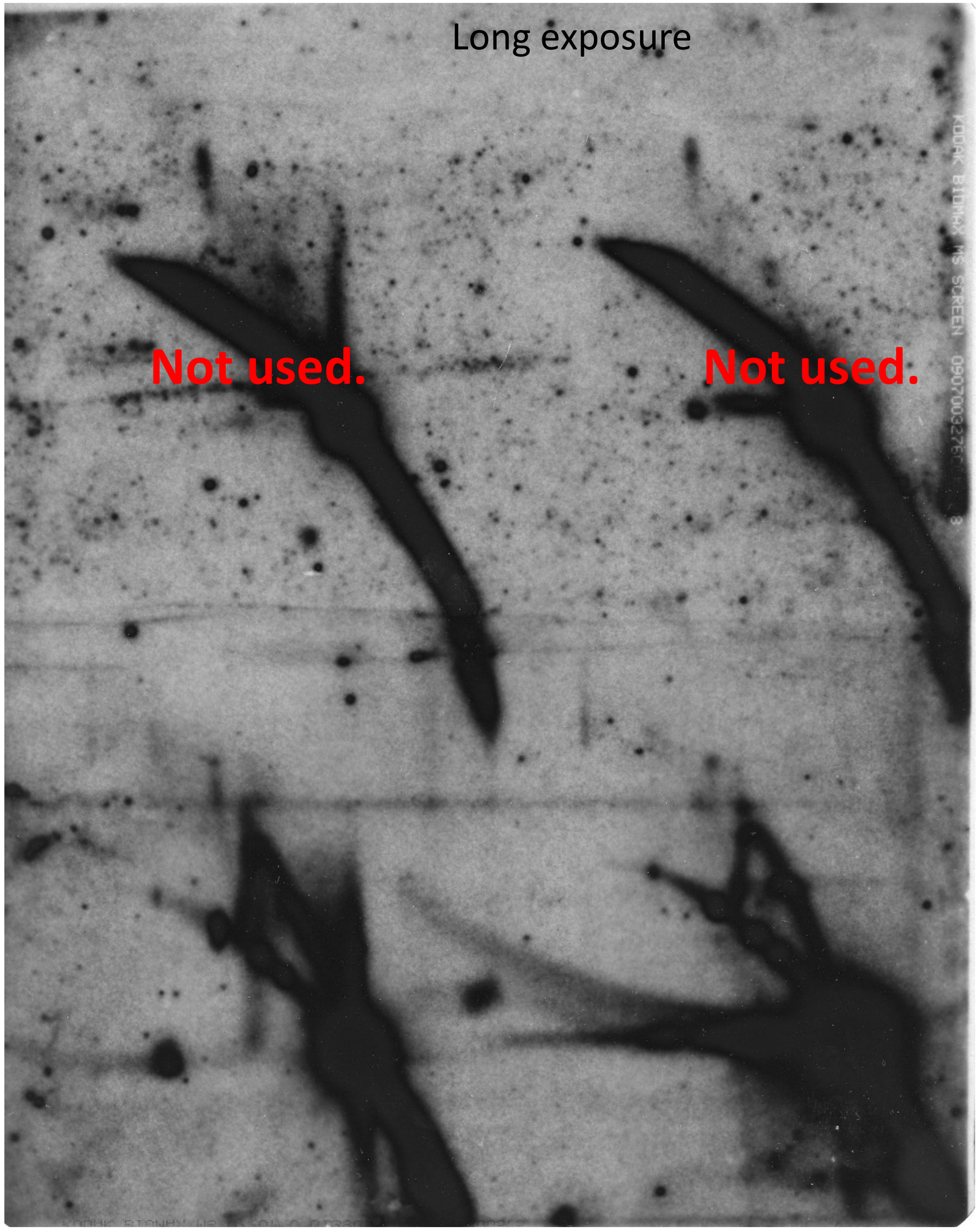

Figure S9

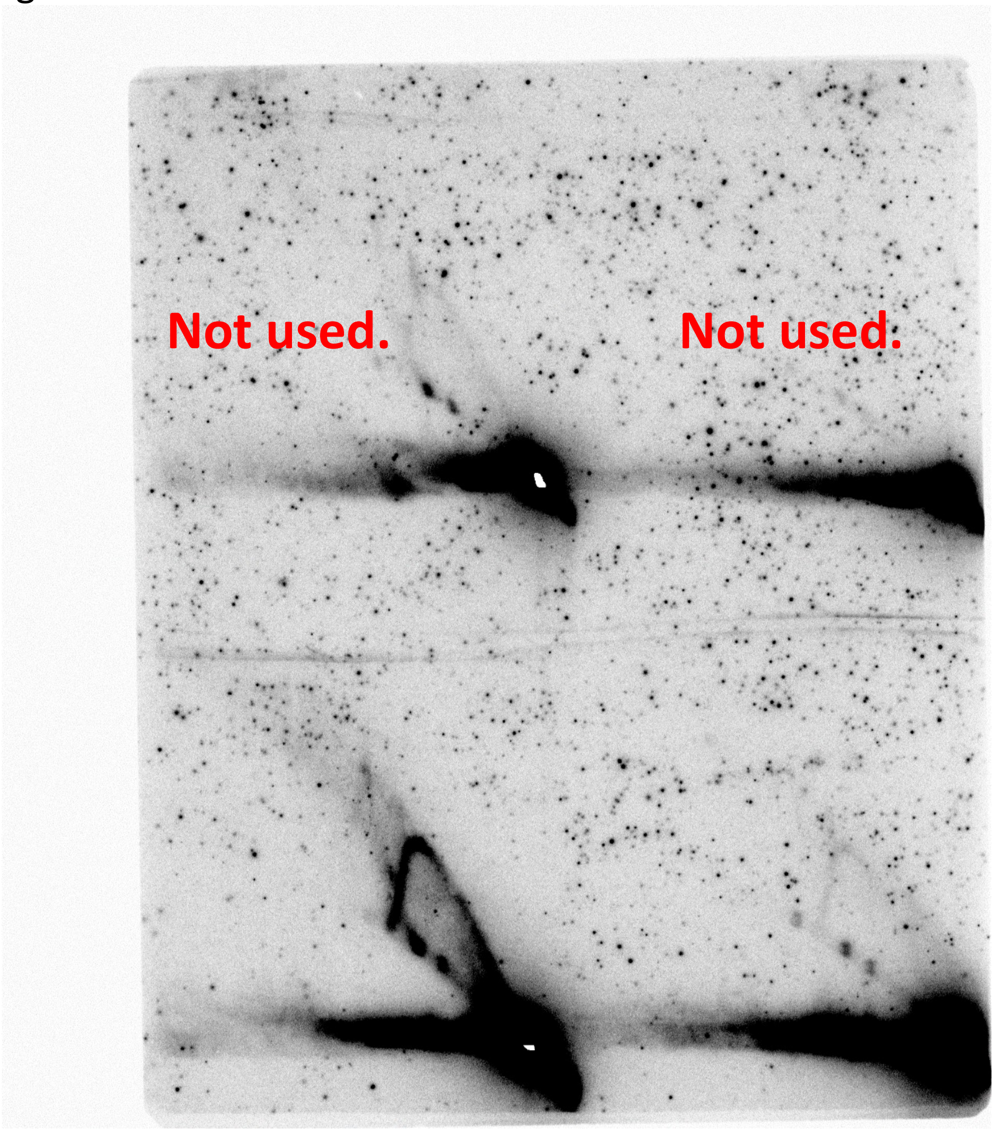

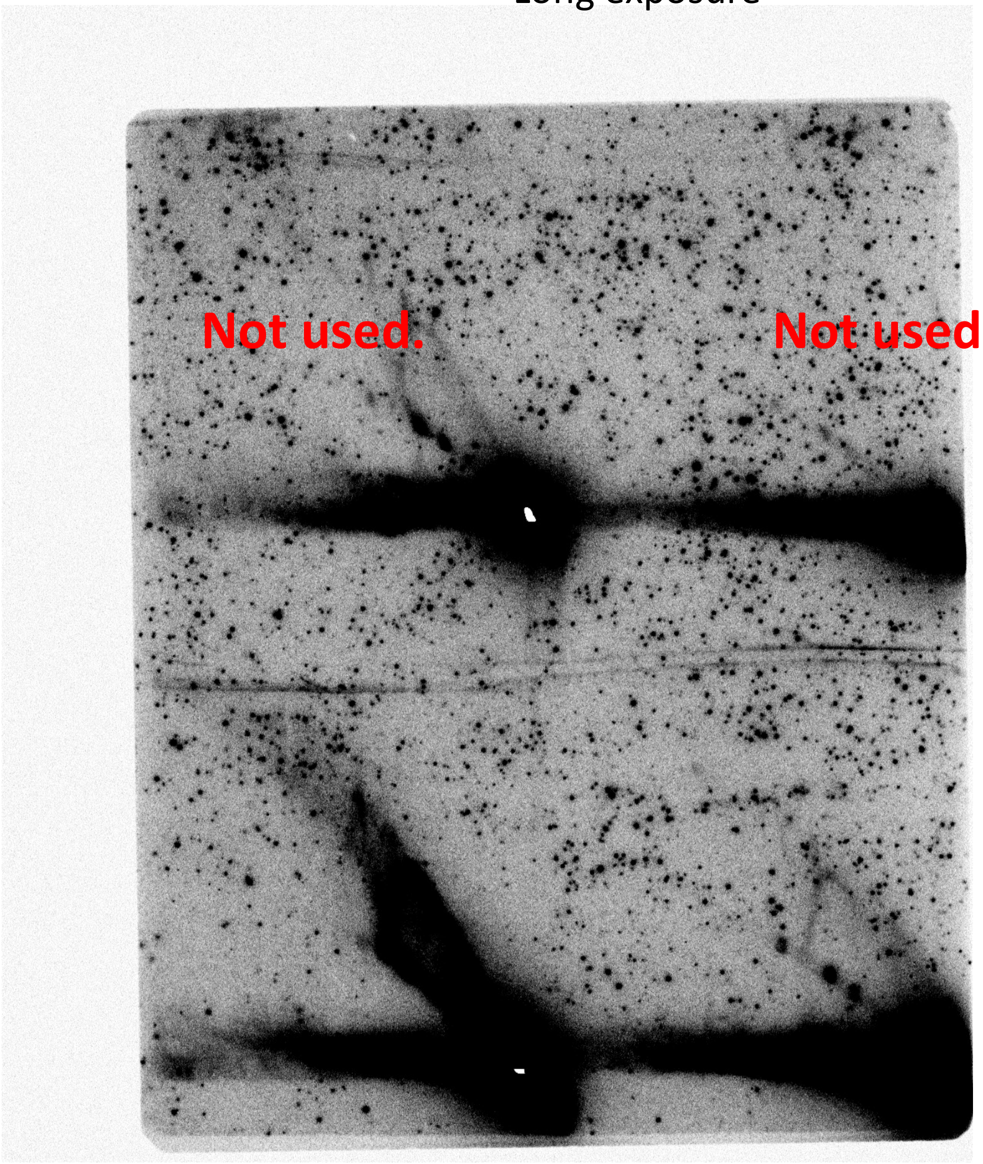

Supplement: Supplementary file 1 — Supplementary data [file 41598_2019_45244_MOESM1_ESM.pdf]
